# Reductions in bacterial viability stimulate the production of Extra-intestinal Pathogenic *Escherichia coli* (ExPEC) cytoplasm-carrying Extracellular Vesicles (EVs)

**DOI:** 10.1371/journal.ppat.1010908

**Published:** 2022-10-19

**Authors:** Min Jiang, Zhongxing Wang, Fufang Xia, Zhe Wen, Rui Chen, Dongyu Zhu, Min Wang, Xiangkai Zhuge, Jianjun Dai

**Affiliations:** 1 MOE Joint International Research Laboratory of Animal Health and Food Safety, College of Veterinary Medicine, Nanjing Agricultural University, Nanjing, China; 2 Department of Nutrition and Food Hygiene, School of Public Health, Nantong University, Nantong, China; 3 College of Pharmacy, China Pharmaceutical University, Nanjing, China; INSERM U1220, FRANCE

## Abstract

Extra-intestinal Pathogenic *Escherichia coli* (ExPEC) is defined as an extra-intestinal foodborne pathogen, and several dominant sequence types (STs) ExPEC isolates are highly virulent, with zoonotic potential. Bacteria extracellular vesicles (EVs) carry specific subsets of molecular cargo, which affect various biological processes in bacteria and host. The mechanisms of EVs formation in ExPEC remains to be elucidated. Here, the purified EVs of ExPEC strains of different STs were isolated with ultracentrifugation processes. A comparative analysis of the strain proteomes showed that cytoplasmic proteins accounted for a relatively high proportion of the proteins among ExPEC EVs. The proportion of cytoplasm-carrying vesicles in ExPEC EVs was calculated with a simple green fluorescent protein (GFP) expression method. The RecA/LexA-dependent SOS response is a critical mediator of generation of cytoplasm-carrying EVs. The SOS response activates the expression of prophage-associated endolysins, Epel1, Epel2.1, and Epel2.2, which triggered cell lysis, increasing the production of ExPEC cytoplasm-carrying EVs. The repressor LexA controlled directly the expression of these endolysins by binding to the SOS boxes in the endolysin promoter regions. Reducing bacterial viability stimulated the production of ExPEC EVs, especially cytoplasm-carrying EVs. The imbalance in cell division caused by exposure to H_2_O_2_, the deletion of *ftsK* genes, or t^6^A synthesis defects activated the RecA/LexA-dependent SOS response, inducing the expression of endolysins, and thus increasing the proportion of cytoplasm-carrying EVs in the total ExPEC EVs. Antibiotics, which decreased bacterial viability, also increase the production of ExPEC cytoplasm-carrying EVs through the SOS response. Changes in the proportion of cytoplasm-carrying EVs affected the total DNA content of ExPEC EVs. When macrophages are exposed to a higher proportion of cytoplasm-carrying vesicles, ExPEC EVs were more cytotoxic to macrophages, accompanied with more-severe mitochondrial disruption and a higher level of induced intrinsic apoptosis. In summary, we offered comprehensive insight into the proteome analysis of ExPEC EVs. This study demonstrated the novel formation mechanisms of *E*. *coli* cytoplasm-carrying EVs.

## Author summary

Bacteria can release extracellular vesicles (EVs) into the extracellular environment. Bacterial EVs are primarily composed of protein, DNA, RNA, lipopolysaccharide (LPS), and diverse metabolite molecules. The molecular cargoes of EVs are critical for the interaction between microbes and their hosts, and affected various host biological processes. However, the mechanisms underlying the biogenesis of bacterial EVs had not been fully clarified in extra-intestinal pathogenic *Escherichia coli* (ExPEC). In this study, we demonstrated ExPEC EVs contained at least three types of vesicles, including outer membrane vesicles (OMVs), outer-inner membrane vesicles (OIMVs), and explosive outer membrane vesicles (EOMVs). Our results systematically identified important factors affecting the production of ExPEC cytoplasm-carrying EVs, especially EOMVs. A reduction in bacterial viability activated the RecA/LexA-dependent SOS response, inducing the expression of endolysins, which increased the production of ExPEC cytoplasm-carrying EVs. This increase in the proportion of cytoplasm-carrying EVs increased the cytotoxicity of EVs. It was noteworthy that antibiotics increased the production of ExPEC EVs, especially the numbers of cytoplasm-carrying EVs, which in turn increased EV cytotoxicity, suggesting that the treatment of infections of multidrug-resistant strains infection with antibiotics might cause greater host damage. Our study should improve the prevention and treatment of ExPEC infections.

## Introduction

Extra-intestinal pathogenic *Escherichia coli* (ExPEC) is responsible for multi-system diseases in humans, other mammals, and birds, including typical urinary tract infections (UTI), neonatal meningitis, bloodstream infections, etc. [1,2]. Based on their different pathogenic subtypes, ExPECs are subdivided into uropathogenic *E*. *coli* (UPEC), neonatal meningitis *E*. *coli* (NMEC), sepsis-associated *E*. *coli* (SEPEC), and avian pathogenic *E*. *coli* (APEC) [3]. ExPECs cause serious public health problems worldwide [4]. The genetic background of ExPEC strains can be determined with modern genotyping methods, including phylogrouping and multilocus sequence typing (MLST). These *E*. *coli* strains can be classified into seven phylogroups (A, B1, B2, C, D, E, F, and cryptic clade I) [5,6]. Using triple PCR method, recent epidemiological research has shown that human and animal ExPEC strains belong to phylogroups B2, D, and F [6–11]. MLST is the method frequently used to define or differentiate commensal *E*. *coli* and ExPEC strains. A limited set of ExPEC-related sequence types (ST) or clonal complexes have been identified in the most clinical ExPEC strains. Manges et al. systematically review the total epidemiological studies (1995–2018) of ExPEC ST lineages [12]. This meta-analysis of ExPEC genotypes showed that 20 common ExPEC STs accounted for 85% of ExPEC strains from diverse extraintestinal infections. In general, pathogenic bacteria that cause foodborne diseases with typical gastrointestinal symptoms, such as *Salmonella* Typhimurium and *Campylobacter jejuni*, are identified as foodborne pathogens. Many studies have suggested that bacterial pathogens with extra-intestinal clinical manifestations may also be foodborne [13]. Recently, Riley systematically reviewed and meta-analyzed the epidemiological observations of ExPEC infections (urinary tract, bloodstream infections, and other colibacillosis) in humans, poultry, livestock animals, and animal food products (meat and dairy) [13]. That review concludes that ExPECs are extra-intestinal foodborne pathogens, and that frequent or point-source exposure to ExPEC-contaminated food is a critical mode of foodborne transmission. Based on the epidemiological observations and opinions reported in these reviews, the dominant foodborne lineages of ExPEC strains contain nine STs (ST10, ST12, ST69, ST73, ST95, ST117, ST127, ST131, and ST405), which are the major avian pathogenic *E*. *coli* (APEC) ST lineages for avian colibacillosis-associated *E*. *coli* isolates [13]. The majority of these ST ExPEC isolates pose a zoonotic risk, especially poultry-to-human transmission [14–16]. Recent studies have shown that ExPEC isolates of ST73, ST95, and ST117 are highly virulent, with zoonotic potential to cause several diseases (sepsis, meningitis, and UTI) in animal models of avian colibacillosis and human infections [17–19].

Bacteria release nano-sized extracellular vesicles (EVs), with sizes ranging from 20 to 400 nm, into the extracellular environment [20]. Bacterial EVs are also called membrane vesicles (MVs) [20,21]. They contain specific subsets of molecular cargo, such as proteins, DNA, RNA, lipopolysaccharide (LPS), and diverse metabolite molecules packaged from the parent bacterium [21,22]. The EVs of Gram-negative bacteria are produced by the blebbing of the bacterial outer membrane (OM) from the envelope, releasing the outer-membrane vesicles (OMVs), which therefore consist of a single-layered membrane. However, despite many years of research, the presence of the bacterial cytoplasmic contents of OMVs has remained obscure, and the mechanism by which OMV cargos (nucleic acids and cytosolic proteins) are acquired is unknown [20]. It is possible that the nucleic acids or cytosolic proteins in some special OMVs do not belong among the bona fide OMVs, which are produced by the blebbing of the bacterial OM, so the cytoplasmic contents have no direct access to OMVs. Recently, scholars have proposed another possibility for EVs production through explosive cell lysis, in which peptidoglycan is degraded by endolysin to induce explosive cell lysis, resulting in the production of cytoplasm-carrying EVs. These are either double-bilayer outer–inner membrane vesicles (OIMVs) or explosive outer-membrane vesicles (EOMVs) with a single-layered membrane [20,23,24]. This novel mechanism for the biogenesis of bacterial EVs has already been demonstrated in *Pseudomonas aeruginosa* and *Bacillus subtilis* [23,24]. However, the general applicability of the mechanism has not been confirmed in other bacteria, such as *E*. *coli*.

Despite the presence of different biogenetic pathways of vesicle formation, these membrane vesicles are collectively referred to as bacterial EVs. In early research, the classic OMVs were considered equivalent to bacterial EVs [25].The secretion of proteins, peptides, nucleotides, lipids, and other signaling factors is a critical process in the short- and long-distance interactions between microbes and their hosts [26]. Bacterial EVs carry specific subsets of proteins, which affect various biological processes in both bacteria–bacteria and bacteria–host interactions, including bacterial virulence, survival, toxin delivery, stress response, export of cellular metabolites, antibiotic resistance, and cell-to-cell communication [20,21,27]. The EVs produced by diarrheagenic *E*. *coli* have been studied in depth [28]. Immunization with *E*. *coli* EVs improved the animal survival after diarrheagenic *E*. *coli* infections [29]. Moreover, *E*. *coli* OMVs release CXCL1/IL-8 from endothelial cells by recruiting neutrophils into the lung, in an NF-κB- and TLR4-dependent manner [30], indicating that OMVs play key roles in inducing the host’s immune response to *E*. *coli* [31,32]. However, fewer studies have addressed the mechanisms of ExPEC EV formation or the associated protein expression profiles [33,34].

In this study, we provide novel insights into the formation mechanism of ExPEC cytoplasm-carrying EVs. The contents of cytoplasm-carrying EVs among ExPEC EVs were accurately quantified. Our results systematically identify important factors that influence the production of ExPEC cytoplasm-carrying EVs, including endolysin and changes in cell stability. An increase in the proportion of the cytoplasm-carrying EVs significantly enhances the cytotoxicity of ExPEC EVs. ExPEC cytoplasm-carrying EVs can be considered as EOMVs or OIMVs, and EOMVs carrying the cytoplasmic proteins were important components of ExPEC EVs. Our study also demonstrates the side effects of antibiotic use in the treatment of bacterial infection from the perspective of EVs.

## Results

### Isolation and purification of ExPEC EVs from different ST strains

We examined the growth curves of three ExPEC strains (FY26, CFT073, and CBE59), FY26 was isolated from chicken [35], CFT073 was isolated from the blood of a patient suffering pyelonephritis [36], and CBE59 was isolated from chicken. Viability counts showed that the three ExPEC strains had similar growth rates (S1A Fig). The EVs produced by the ExPEC strains cultured for 4h (early log of bacterial growth), 8h (log phase), 12h (early stationary phase), or 16h (stationary phase) were collected from the cell-free supernatants by ultracentrifugation. The amount of protein in EVs was estimated with a bicinchoninic acid (BCA) assay (S1B Fig). The EV production of each strain increased as the culture time increased. The protein concentration was clearly higher in the CBE59 EVs than in the CFT073 and FY26 EVs.

**Fig 1:**
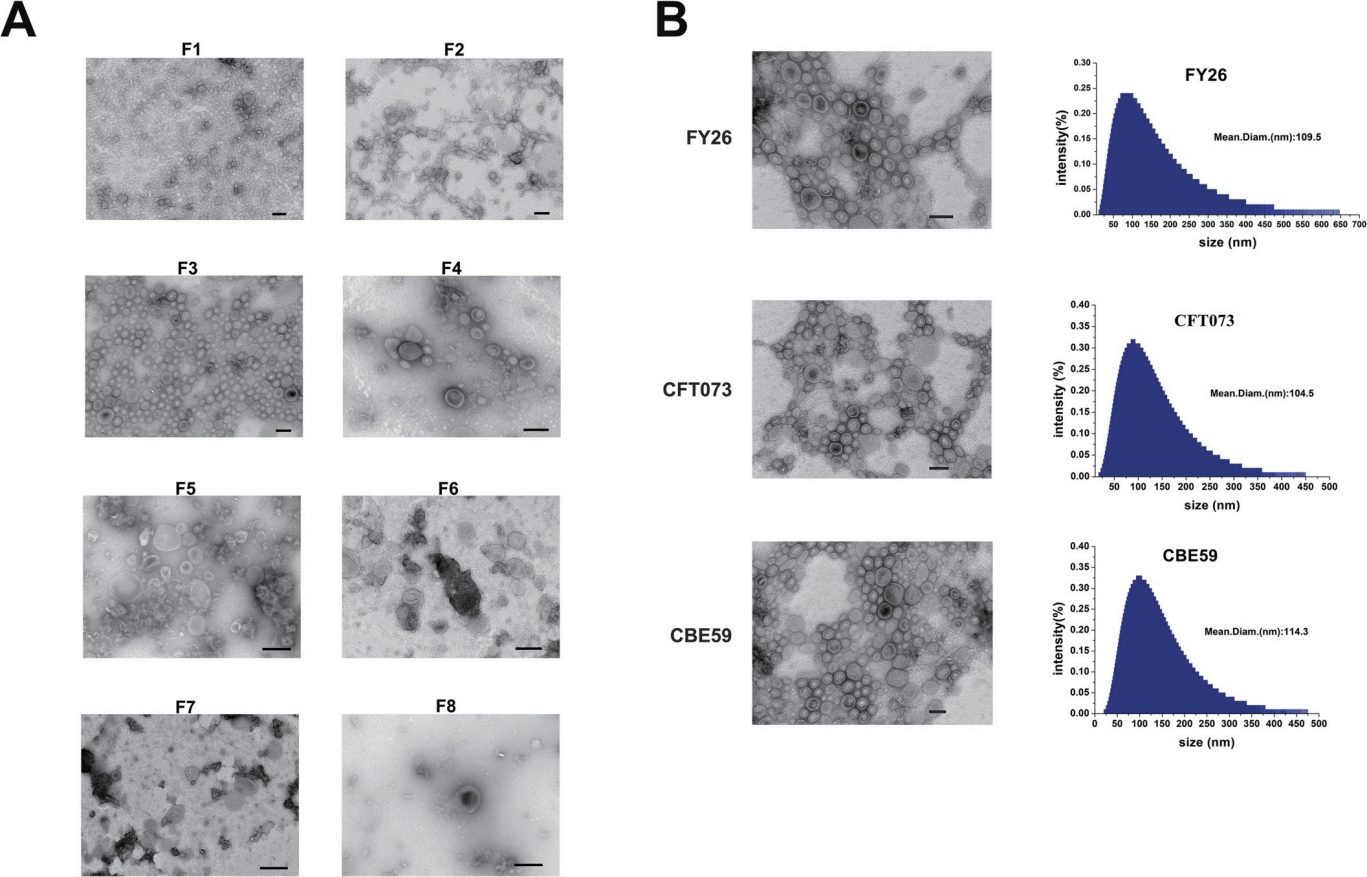
TEM visualization of the EVs produced by ExPEC strain. **(A)** The purified FY26 EVs obtained from fractions F1–F8 were visualized with TEM. Scale bars: 200 nm. **(B)** DGU-purified EVs of different ExPEC strains (FY26, CBE59, and CFT073) were observed with TEM. The size distributions of the three purified EVs were detected with Dynamic light scattering (DLS). Scale bars: 200 nm.

The EVs of the three ExPEC strains (FY26, CBE59 and CFT073) grown for 12h were obtained from liquid cultures by ultracentrifugation. To extract the FY26 EVs, for example, a large vesicle pellet of FY26 was isolated from the culture supernatant by ultracentrifugation (S1C Fig). After re-suspension, the FY26 EVs were observed with transmission electron microscopy (TEM), which revealed that the isolated FY26 EVs contained bacterial extracellular structures (S1D Fig). The vesicle pellet was then purified with OptiPrep density gradient ultracentrifugation (DGU) to minimize the contaminants in the FY26 EVs. After DGU, the particle sample was successfully separated, and the tubes were labeled according to 10 fractions (F1-F10) (S1E Fig). The protein concentration in each fraction was measured with a BCA assay (S1F Fig). The results suggested that bacterial vesicles were present in DGU fractions F2, F3, and F4, which showed relatively high protein concentrations. The purified FY26 EVs obtained from these fractions (F1-F8) were confirmed with TEM (Fig 1A). The FY26 EVs were enriched in fractions F2, F3, and F4 (Fig 1A). There were some FY26 EVs occurred in fractions F5-F8, accompanied by obvious impurities and bacterial contaminants. Finally, fractions F2, F3, and F4 were further purified with a second round of ultracentrifugation. We also extracted the DGU-purified EVs of strains CFT073 and CBE59, and confirmed them with TEM (Fig 1B). The size distributions and concentrations of the purified EVs of strains FY26, CFT073 and CBE59 were quantified with dynamic light scattering (DLS) and a NanoSight nanoparticle tracking analysis (NTA) (Figs 1B and S1G). The EVs of the three ExPEC strains were heterogeneous and displayed multiple spherical vesicles, with diameters in the range of 20–500 nm. Dynamic light scattering (DLS) and TEM results showed that the number of EVs with diameter less than 50nm accounted for about 9% to 20%.

### Comparative analysis of global protein showed that various bacterial proteins were enriched in ExPEC EVs

**Fig 2:**
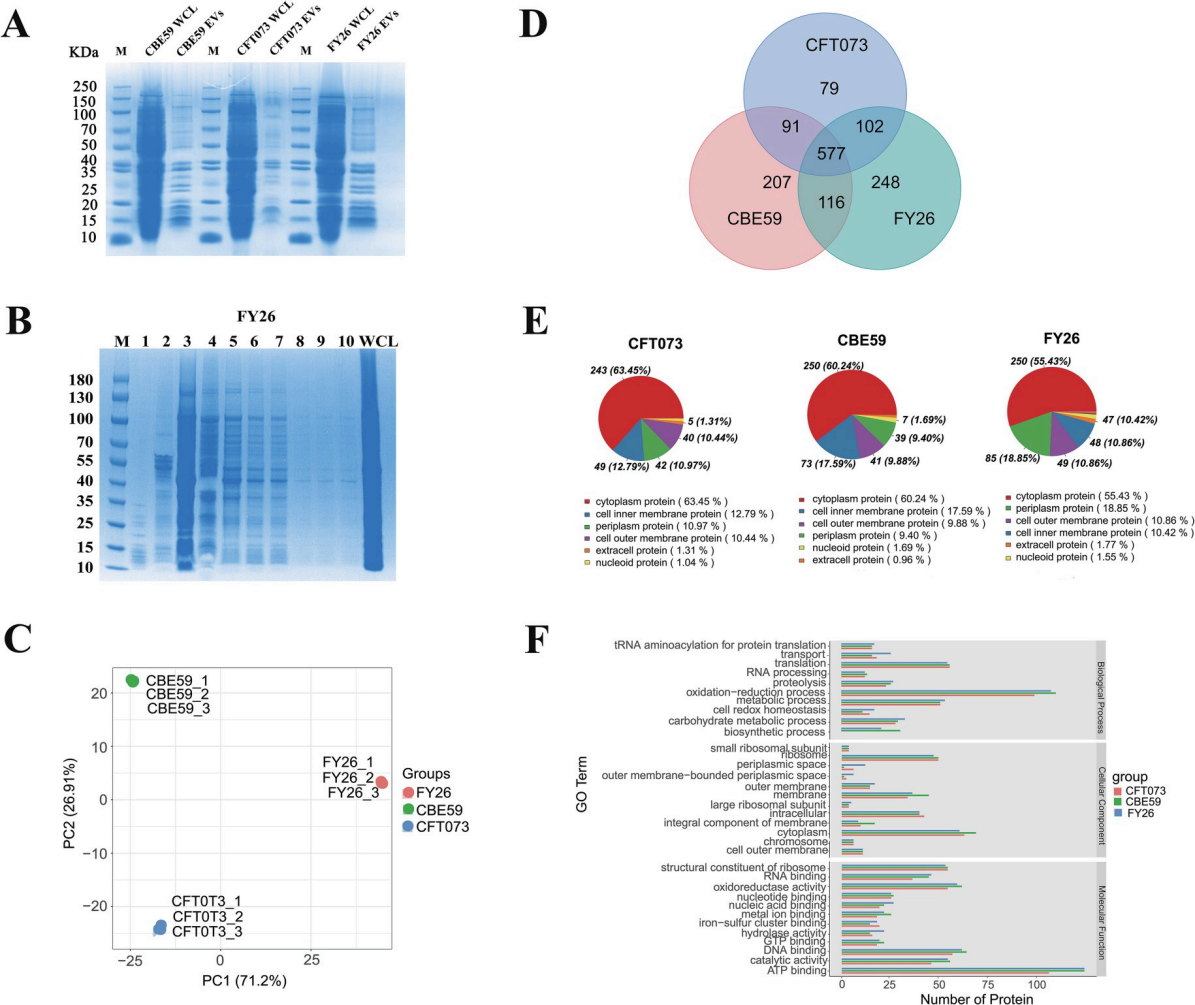
Comparative analysis of total proteins from ExPEC EVs. **(A)** Density-gradient ultracentrifugation (DGU)-purified EVs of ExPEC strains FY26, CBE59, and CFT073 were analyzed with SDS-PAGE. Total protein (5 μg) from EVs or 15 μg of total protein from whole-cell lysates (WCLs) was loaded into the lanes. Lane M: protein marker. **(B)** ExPEC EVs from different density gradient fractions were analyzed with SDS-PAGE. Fractions (15 μL loaded onto the gel) are numbered according to increasing density. The images show one representative experiment. M: Protein marker; WCL: 5 μg loaded into each well of the gel. **(C)** Principal components analysis (PCA) of the EV proteomes of the ExPEC strains. The graph shows three cluster patterns with overlapping features for CFT073 (red cluster), CBE59 (green cluster), and FY26 (blue cluster). **(D)** Comparison of EV protein profiles in different ExPEC strains with a Venn diagram. Venn diagram indicates the numbers of identified proteins in the EVs of the three ExPEC strains. **(E)** Classification of the EV proteins identified in different ST strains by subcellular location. Subcellular localization of the identified EV proteins was predicted with CELLO (http://cello.life.nctu.edu.tw). **(F)** Functional classification of EV unigenes identified in the three ExPEC strains was determined with Gene Ontology (GO). The GO enrichment analysis identified three categories: cellular component, molecular function, and biological process.

To study the EV-associated proteins, the protein profile of the EVs from each strain was analyzed with SDS-PAGE (Fig 2A). Relatively few protein bands from EVs of these ExPEC strains were detected (Fig 2A), and fractions F2-F4 contained more proteins than the other fractions (Figs 2B, S2A, and S2B). Label-free mass spectrometry was used to identify the protein components of the DGU-purified EVs of strains FY26, CFT073, and CBE59, after culture for 12 h. A principal components analysis (PCA) showed that the proteomic profiles of the DGU-purified EVs (with three independent label-free proteomic repetitions for each strain) were highly consistent (Fig 2C). We identified 1,043 proteins in FY26 EVs, 849 proteins in CFT073 EVs, and 991 proteins in CBE59 EVs (S1 Table). The EVs isolated from the different ExPEC strains had clearly dissimilar proteomes (Fig 2D), and shared 577 proteins, whereas 248, 207, and 79 proteins were only detected in FY26 EVs, CBE59 EVs, and CFT073 EVs, respectively (Fig 2D). A subcellular localization prediction program was used to determine the subcellular locations of the identified EV proteins. Of the predicted proteins in the FY26 EVs proteome, cytoplasmic proteins accounted for 55.43%, and 10.86% of proteins were from the outer membrane. Of the predicted proteins in the CBE59 EVs proteome, 60.24% were from the cytoplasm, and 9.88% were from the outer membrane. Of the predicted proteins in the CFT073 EV proteome, cytoplasmic protein accounted for 63.45%, and 10.44% of proteins were from the outer membrane (Fig 2E).

The functional predictions for EV proteins in the ExPEC of different ST types strains were analyzed with the Gene Ontology (GO), Clusters of Orthologous Groups (COG), and Kyoto Encyclopedia of Genes and Genomes (KEGG) databases. The GO analysis indicated that the proteins enriched in the FY26 EVs were involved in oxidation reduction and metabolic processes: ATP binding, DNA binding, and oxidoreductase activity. The proteins from the EVs of the three ExPEC strains (FY26, CFT073 and CBE59) showed analogous functional patterns. ATP binding components were significantly enriched in the EVs from all three ExPEC strains (Fig 2F). Similarly, the COG analysis showed that the proteins enriched in the ExPEC EVs were mainly involved in amino acid transport and metabolism, translation, ribosomal structure and biogenesis, energy production and conversion, and cell wall, membrane, and envelope biogenesis (S2C Fig). Fewer proteins in the CBE59 EVs were involved in posttranslational modification, protein turnover, and chaperones, and fewer proteins in the CFT073 EVs were involved in prophages and transposons (S2C Fig). The KEGG analysis showed that many proteins were involved in carbohydrate metabolism, amino acid metabolism, and translation (S2D Fig).

### Quantification and bioinformatic analysis showed the enrichment of cytoplasmic proteins in ExPEC EVs

**Fig 3:**
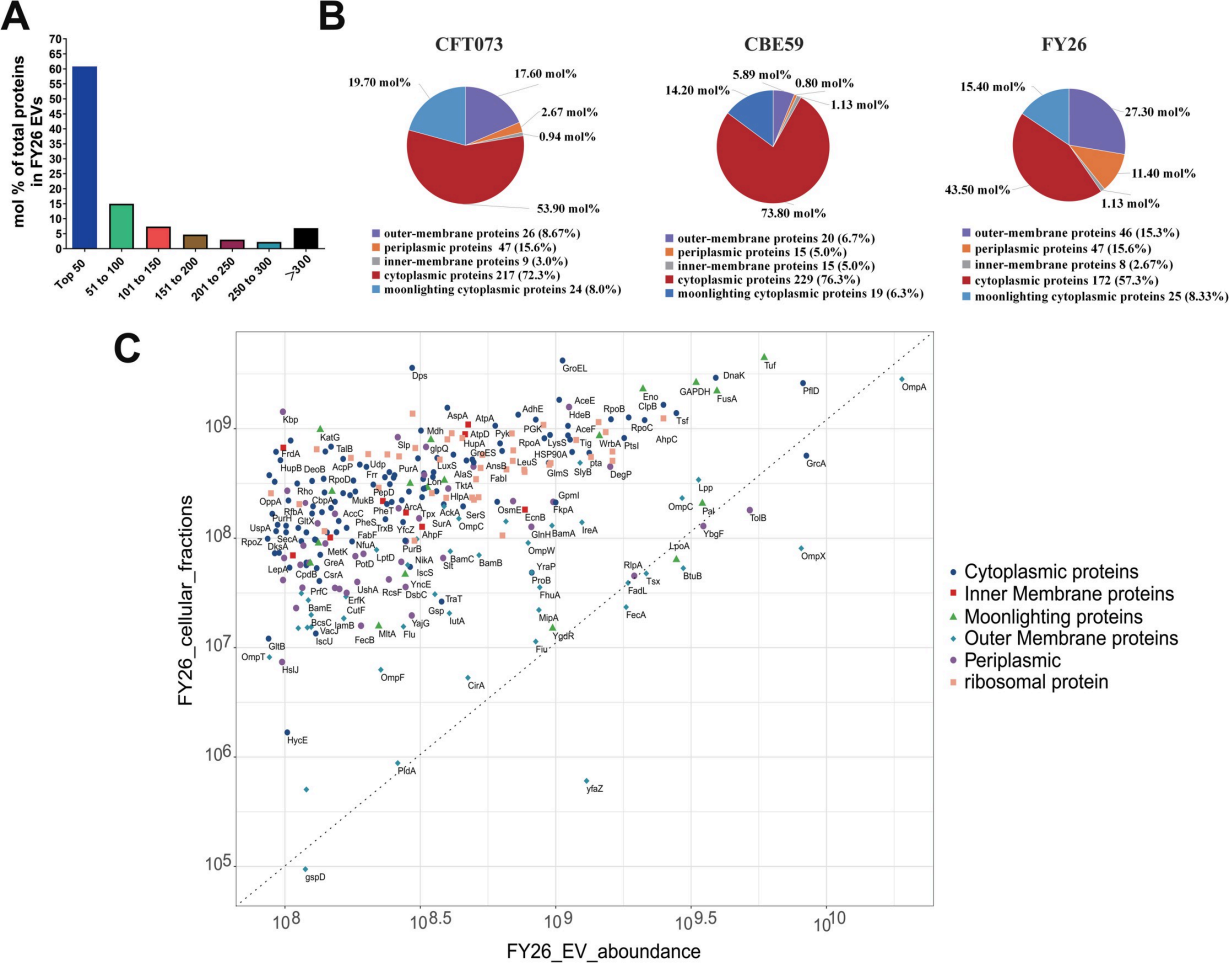
Quantification and bioinformatic analysis of the proteins enriched in ExPEC EVs. **(A)** Abundances of the 300 most-abundant proteins of FY26 EVs relative to the total protein. The horizontal axis indicates 50 proteins sorted by abundance from most to least abundant relative to the total protein abundance, and the vertical axis indicates the protein abundance relative to the total protein. **(B)** Subcellular localization classification of the 300 most-abundant proteins identified in EVs from different ExPEC strains (FY26, CBE59, and CFT073). Subcellular localization of the identified proteins was determined with CELLO (http://cello.life.nctu.edu.tw). The numbers of the proteins are displayed below the pie chart, and the percentage abundances are shown in the pie chart. **(C)** The 300 most-abundant proteins isolated from the FY26 EV fractions were compared with their abundances in the total FY26 cellular fractions. Subcellular localization is shown as follows: outer-membrane proteins (light blue), periplasmic proteins (purple), inner-membrane proteins (red), cytoplasmic proteins (deep blue), moonlighting proteins (green), and ribosomal subunit proteins (orange). Proteins enriched in the FY26 EVs are shown below and to the right of the dashed line, and proteins depleted in the EVs are shown above and to the left of the line. The data for this figure can be found in S1 Table.

Until now, there have been few quantitative proteomic analyses of the composition of EV proteins from different ExPEC strains. To measure the relative abundances of the protein cargoes in the FY26 EVs, the raw data were analyzed with MaxQuant. Based on the intensity-based absolute-protein quantification (iBAQ) metric, the average normalized abundance of the FY26 EVs ranged from 2.1×10^6^ to 1.9×10^10^ (S1 Table). In total, the 300 most-abundant proteins accounted for 93.1 mol% of the proteins in the FY26 EVs (S1 Table). We calculated the abundance ratio per 50 proteins, sorted from the most to the least abundant relative to the total protein abundances of the DGU-purified EVs (Fig 3A). Similarly, the 300 most-abundant proteins accounted for 95.1 mol% of the proteins in the CFT073 EVs, and 96.0 mol% in the CBE59 EVs. An analysis of their subcellular localization showed that the 300 most-abundant proteins of the FY26 EVs contained 172 (57.3%) cytoplasmic proteins, accounting for 43.5 mol% of these proteins (Fig 3B). The subcellular localization of the CBE59 and CFT073 EVs was also shown in Fig 3B. These findings suggested that cytoplasmic proteins were the major protein component in ExPEC EVs. This result is inconsistent with previous reports, which showed that outer-membrane proteins are the major components of ExPEC EVs [37–39].

To identify the specific proteins enriched in the FY26 EVs, we determined the protein composition of the FY26 cellular fractions (whole-cell lysates, WCLs). Overall, we identified a total of 2,742 proteins in the FY26 cellular fractions in the early stationary growth phase at 12 h (S2 Table). This proteome represented 61.5% of the predicted proteins from the FY26 reference genome, highlighting the significance of peptide coverage, protein matching, and abundance quantification. To measure the protein enrichment, enrichment analysis was performed using averaged abundance values from proteomes of the FY26 EVs and WCLs (Fig 3C). No matter from EVs or WCLs, there seemed to be non-discernible abundance difference for these particular membrane proteins, belonging to 300 most-abundant proteins of FY26 EVs. Proteins enriched in the FY26 EVs are shown beneath and to the right of the dashed line, whereas proteins depleted in the EVs appear above and to the left of the line (Fig 3C). The most enriched outer-membrane proteins (such as OmpA, OmpX, BtuB, FecA, FadL, GspD, PidA, Tsx, and YfaZ) and several periplasmic proteins (such as TolB, YbgF, and RipA) in FY26 EVs were also high abundant in FY26 WCLs. However, most cytoplasmic proteins presented a lower enrichment in the FY26 EVs relative to those in FY26 WCLs. Several moonlighting proteins (such as Tuf, FusA, and Pal) were clearly enriched in the FY26 EVs, whereas other proteins (such as KatG and Mdh) showed very low abundances, compared with their abundances in the FY26 WCLs. The relative enrichment of the 300 most-abundant proteins in FY26 and CFT073 EVs was compared (S2E Fig). The most-abundant outer-membrane proteins in the FY26 EVs were also significantly enriched in the CFT073 EVs. However, the common abundant cytoplasmic proteins (including GroEL and GrcA) in FY26 EVs showed vastly different enrichment in the CFT073 EVs. We also compared the relative enrichment of the 300 most-abundant proteins in the FY26 EVs with their enrichment in the CBE59 EVs (S2F Fig). The most-abundant moonlighting proteins in the FY26 EVs were also significantly enriched in the CBE59 EVs. Notably, the FY26 EVs contained more outer-membrane proteins and periplasmic proteins than the CBE59 EVs. However, ribosomal proteins were more enriched in the CBE59 EVs than in the FY26 EVs. The most-abundant cytoplasmic proteins in FY26 EVs were also significantly enriched in the CBE59 EVs. The relative enrichment of the 300 most-abundant proteins in the CBE59 and CFT073 EVs was compared (S2G Fig). The most-abundant cytoplasmic proteins in the CFT073 EVs were also significantly enriched in CBE59 EVs. Outer-membrane proteins were significantly enriched in the CFT073 EVs relative to their abundances in CBE59 EVs. In particular, ribosomal proteins were more enriched in the CBE59 EVs than in the CFT073 EVs. Collectively, although membrane proteins, such as OmpA, OmpX, and Lpp, were enriched in the ExPEC EVs, cytoplasmic proteins were also abundantly packaged into the ExPEC EVs. The subcellular distributions and abundances of the EV proteins in ExPEC strains of different ST types showed obvious similarities.

### Identification of the membrane and cytoplasmic proteins in DGU-purified EVs

**Fig 4:**
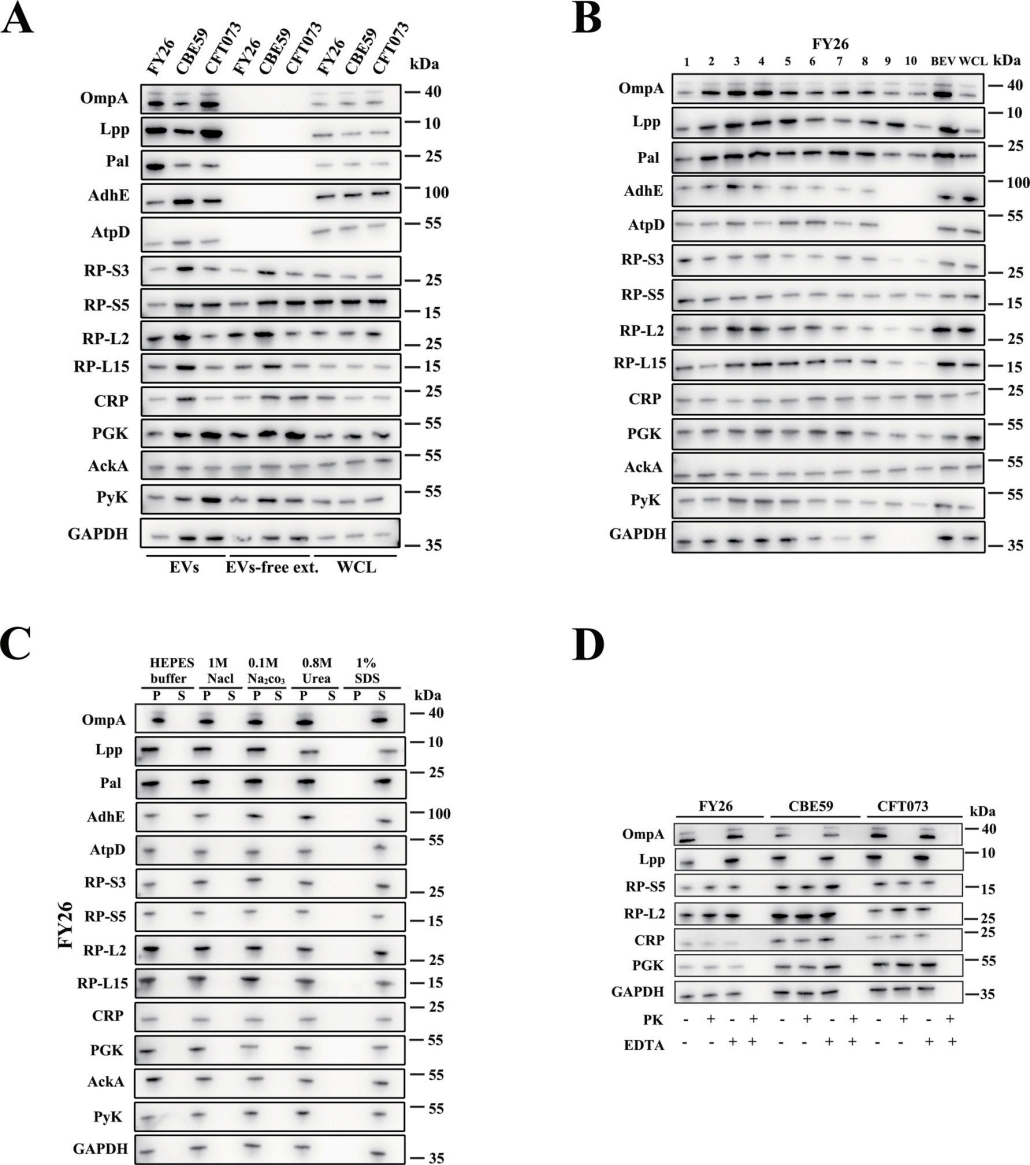
Detection of membrane and cytoplasmic proteins in density gradient ultracentrifugation (DGU)-purified ExPEC EVs. **(A)** Expression of membrane and cytoplasmic proteins in EVs and EV-free extracellular medium of ExPEC strains was determined with western blotting. Total protein (1 μg) was loaded into the OmpA and Gapdh lanes, and 5 μg of total protein was loaded into the other lanes. Molecular weight markers are shown on the right. **(B)** Expression of membrane and cytoplasmic proteins in DGU-purified EVs (F1–F10) was determined with western blotting. Nonfractionated EVs were used as the positive controls, and whole-cell lysates (WCLs) were used as the loading controls. Total protein (1 μg) was loaded into the OmpA and Gapdh lanes, and 5 μg of total protein was loaded into the other lanes. **(C)** Dissociation assays confirmed that the protein cargoes were tightly associated with the EVs of the ExPEC strains. OptiPrep-purified EVs were treated with HEPES buffer containing the indicated chemical agents or with HEPES buffer only. The pellets (P; containing EVs) and extracellular media (S; containing proteins released from EVs) were collected by ultracentrifugation, and the samples were analyzed with western blotting. Total protein (1 μg) was loaded into the OmpA and Gapdh lanes, and 5 μg of total protein was loaded into the other lanes. **(D)** Immunoblots of proteinase K (PK)-untreated (PK-) and PK-treated (PK+) ExPEC EVs either intact (EDTA+) or lysed with 0.1 M EDTA (EDTA+) with the indicated antibodies.

Several reports have shown that outer-membrane proteins are enriched in bacterial OMVs [40,41]. However, according to our findings, cytoplasmic proteins may be the major components of the proteomes of purified ExPEC EVs. This phenomenon has also been observed in several previous studies [20,34,38,42]. The cAMP-activated global transcriptional regulator CRP was abundant in our studied EV proteomes. CRP acts as a bacterial lysis marker when the release of cytoplasmic proteins into extracellular medium is assessed [43]. The isolated EVs and the EV-free extracellular medium were analyzed with immunoblotting to determine whether cytoplasmic proteins were released into the EV-free extracellular medium. As shown in Fig 4A, outer-membrane proteins (OmpA, lipoprotein Lpp, and Pal), inner-membrane proteins (AdhE and ATP synthase subunit beta [AtpD]), cytoplasmic proteins (CRP, acetate kinase AckA, phosphoglycerate kinase PGK, and pyruvate kinase Pyk), a moonlighting cytoplasmic protein (GAPDH), and cytoplasmic ribosomal proteins (RP-L2, RP-L15, RP-S3, RP-S5) were detected. The specificity of the antibodies corresponding to these *E*. *coli* membrane or cytoplasmic proteins was verified by western blotting (S3 Fig). Moreover, the cytoplasmic proteins (CRP, AckA, PGK, and Pyk) and ribosomal proteins were distributed almost evenly between the EVs and EV-free extracellular media of the ExPEC strains (FY26, CFT073, and CBE59) (Fig 4A). However, the membrane proteins (OmpA, Lpp, Pal, AdhE, and AtpD) were mainly detected in the ExPEC EVs.

Density gradient fractionation of the ExPEC EVs and the western blotting analysis of the EV fractions for the proteins mentioned above demonstrated that the different fractions differed in the abundances of their protein cargoes (Figs 4B and S4A). Specifically, these EV-associated proteins were enriched in fractions F2-F4. A dissociation assay was performed to confirm the tight association between the EVs and their respective protein cargoes, and EVs were lysed with 0.1 M EDTA. Sodium dodecylsulfate (SDS), a strong membrane disrupting agent, was the only chemical capable of releasing the detected proteins from the ExPEC EVs (Figs 4C and S4B). Our findings suggested that inner-membrane and cytoplasmic proteins could in fact be packaged and enriched in ExPEC EVs. The mechanisms by which and why inner-membrane and cytoplasmic proteins are incorporated into bacterial vesicles are current research hotspots. One possible mechanism is via the production of bilayered OIMVs, as an important component in ExPEC EVs. Moreover, the 30S- and 50S-ribosome proteins are highly enriched in and tightly associated with ExPEC EVs, suggesting that ExPEC EVs contained many ribosomes. To localize outer-membrane and cytoplasmic proteins, the ExPEC EVs were digested with proteinase K (PK) digestion, whereas the proteins inside the EVs were not degraded by proteinase K. The results showed that the cytoplasmic proteins (RP-L2, RP-S5, GAPDH, CRP and PGK) were not degraded by PK, indicating that these proteins were located inside the EVs (Fig 4D). However, the outer-membrane proteins (OmpA and Lpp) were degraded by PK, indicating that they were located on the outsides of the EVs (Fig 4D).

### ExPEC EVs are a mixture of classical OMVs and cytoplasm-carrying vesicles

**Fig 5:**
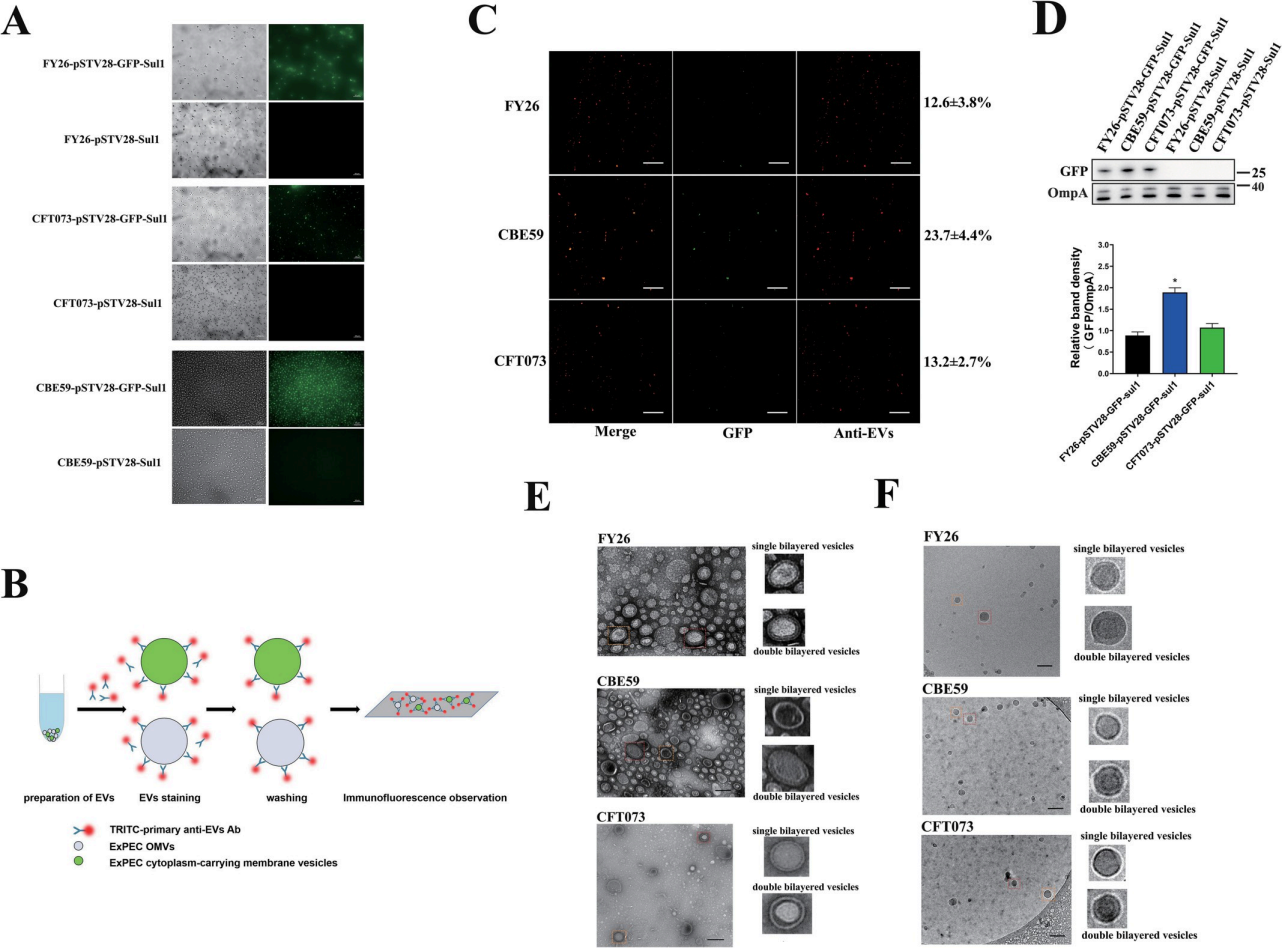
ExPEC EVs contain multiple types of vesicles. **(A)** Green fluorescent protein (GFP) in ExPEC strains was visualized with fluorescence microscopy. Scale bars: 20 μm. **(B)** Illustration of experiment to detect cytoplasm-carrying membrane vesicles in ExPEC EVs. **(C)** Immunofluorescent images of EVs produced by ExPEC strains. The EVs were stained with TRITC-labeled primary mouse anti-EV antibody. The coexistence ratio of GFP-carrying vesicles (green fluorescence) among the total EVs (red fluorescence) represents the percentage of cytoplasm-carrying membrane vesicles, which is indicated on the right. Data were obtained from at least three independent experiments. Graphs show a representative image. Scale bars: 10 μm. **(D)** GFP and OmpA proteins in EVs were determined with western blotting. **(E)** The outer–inner-membrane vesicles (OIMVs) of ExPEC were observed with transmission electron microscopy (TEM). ExPEC EVs displayed a double bilayered structure (red square), or single bilayered structure (orange square). Scale bars: 200 nm. **(F)** The outer–inner-membrane vesicles (OIMVs) of ExPEC were observed with Cryo-TEM. ExPEC EVs displayed a double bilayered structure (red square), or single bilayered structure (orange square). Scale bars: 200 nm.

Because ExPEC EVs carried a large number of cytoplasmic proteins, we inferred that these EVs included another kind of vesicles carrying cytoplasm, such as EOMVs and OIMVs. Therefore, we designed an experiment in which bacteria were transformed with plasmid pSTV28-GFP-*sul1*, which carried GFP-encoded gene and resistance gene *sul1*. Immunofluorescence assays showed that these ExPEC strains (FY26, CBE59 and CFT073) expressed higher levels of GFP than the control cells (Fig 5A). DGU-purified ExPEC EVs were then collected and stained with tetramethylrhodamine (TRITC)-labeled primary antibody. The co-existence of GFP and fluorescent antibody (red fluorescence) signals in the stained EVs indicated the presence of membrane vesicles carrying cytoplasmic proteins in the ExPEC EVs. The design of our experiment is shown in Fig 5B. The number of fluorescently labeled vesicles in each image was counted directly with the Image J software and visual proofreading. The ratio of GFP-carrying vesicles to total red-fluorescence-stained EVs indicated the percentage of cytoplasm-carrying membrane vesicles. The remaining proportion might be the proportion of classical OMVs, formed by the blebbing of the bacterial outer membrane from the envelope. Cytoplasm-carrying vesicles was detected among the CBE59 EVs (Fig 5C). At 12 h (early stationary phase), the proportion of cytoplasm-carrying membrane vesicles in the CBE59 EVs was about 23.7±4.4%, whereas the proportion of cytoplasm-carrying vesicles was about 12.6±3.8% in the FY26 EVs, and 13.2±2.7% in the CFT073 EVs (Fig 5C). Moreover, the expression level of GFP protein in ExPEC EVs was determined with western blot. The result indicated that the proportion of cytoplasm-carrying EVs produced by ExPEC was consistent with the expression level of GFP protein (Fig 5D). These results indicated that ExPEC EVs consisted of two mainly kinds of vesicles (classical OMVs and cytoplasm-carrying vesicles).

To determine whether there are multiple types of extracellular vesicles, TEM was used to analyze the membrane structures of the ExPEC EVs. As shown in Fig 5E, TEM revealed that the FY26 EVs contained specific vesicles with a double bilayered structure, so-called OIMVs, or single bilayered structure. Cryo-TEM images also revealed the presence of OIMVs in the FY26 EVs (Fig 5F). Similarly, the OIMVs were observed among the CBE59 and CFT073 EVs by TEM, respectively (Fig 5E). As shown in Fig 5F, Cryo-TEM images confirmed the presence of double bilayered vesicles (specifically OIMVs) among CBE59 and CFT073 EVs. However, electron microscope observation showed that the number of the OIMVs in these ExPEC EVs was very few.

### Defect in crosslinking between peptidoglycan and outer membrane increases the formation of ExPEC OMVs

**Fig 6:**
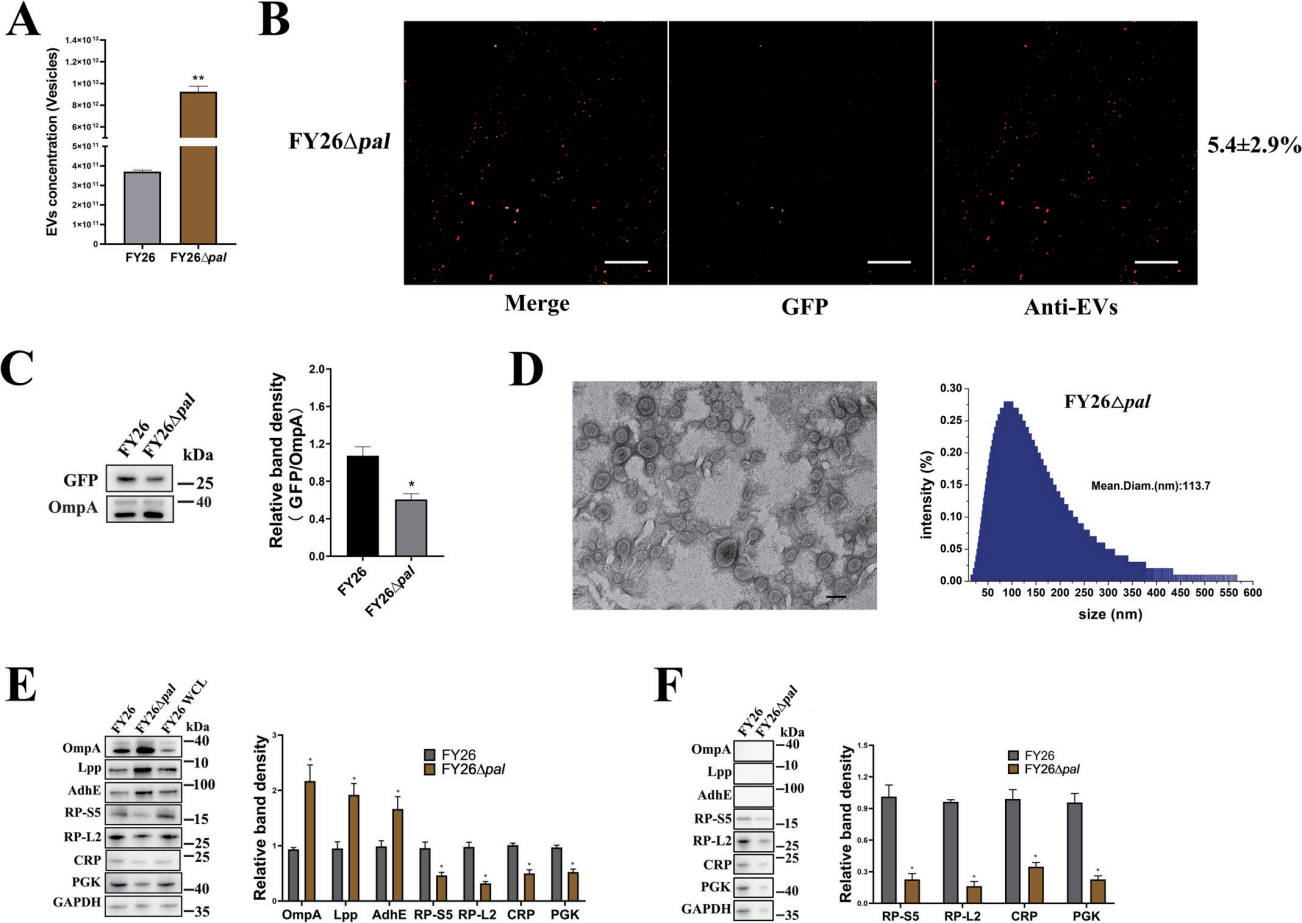
Deletion of *pal* gene affects the production of ExPEC outer-membrane vesicles (OMVs). **(A)** The concentrations of purified EVs produced by FY26 and FY26Δ*Pal* were determined with a nanoparticle tracking analysis (NTA). **(B)** Immunofluorescent detection of EVs produced by FY26Δ*pal*. The percentage of GFP-carrying vesicles (green fluorescence) is indicated on the right. Data were obtained from at least three independent experiments, with three replicates. Graph shows a representative image. Scale bars: 10 μm. **(C)** GFP and OmpA proteins in EVs were determined with western blotting. **(D)** The EVs produced by FY26Δ*Pal* were observed with transmission electron microscopy (TEM), and the size distributions of the purified EVs were detected with Dynamic light scattering (DLS). Scale bars: 200 nm. **(E)** Levels of membrane and cytoplasmic proteins in EVs from FY26 and FY26Δ*pal* were determined with western blotting. **(F)** Levels of membrane and cytoplasmic proteins in EV-free extracellular medium of FY26 and FY26Δ*pal* were determined with western blotting.

OMVs protrude during membrane blebbing, which results from the unbalanced biosynthesis of the cell envelope. In common blebbing model, the crosslinking between the outer membrane and peptidoglycan is disrupted, and the separation of the outer membrane from the peptidoglycan layer promotes the formation of membrane blebbing [21,25,44]. Previous reports have shown that more than 10 bacterial membrane or periplasmic proteins (such as the Tol-Pal complex, murein lipoproteins Lpp, and NlpI, etc.) function in peptidoglycan crosslinking and the maintenance of cell envelope integrity, and are involved in the production of OMVs [45]. Mutants of these proteins in *E*. *coli* showed defects in crosslinking and the increased production of OMVs relative to those in wild-type (WT) strains [33,46,47]. Reimer et al. also report that deletion of *tolA* reduces the crosslinking between the outer and inner membranes, leading to the formation of OMVs, OIMVs, or vesicles with even more membranes [48]. We confirmed that the peptidoglycan-associated outer-membrane protein Pal could predominantly affect the production of ExPEC OMVs. NTA results showed that the deletion of *pal* led to an increase (24.9-fold) in vesicle production of the mutant FY26Δ*pal* relative to that in WT FY26 (*P*≤0.01) (Figs 6A, S5A, and S5B). Similarly, a BCA protein assay revealed that the concentration of proteins was higher (5.1-fold) in FY26Δ*pal* EVs than in WT FY26 EVs (*P* ≤ 0.01) (S5C Fig). Moreover, immunofluorescence assays showed that the proportion of cytoplasm-carrying vesicles in the mutant strain FY26Δ*pal* was 5.4±2.9% (Fig 6B). And the expression level of GFP protein in the EVs produced by mutant strain FY26Δ*pal* was determined with western blot. The level of GFP protein was reduced in the EVs of FY26Δ*pal* relative to that in the WT FY26 (Fig 6C). TEM images showed predominantly single-layered vesicles (OMVs) and a few vesicles with double-bilayer membrane (OIMVs) (Fig 6D). DLS result showed that the diameter of FY26Δ*pal* EVs was 20–500 nm (Fig 6D). To further investigate the level of EV-associated proteins in strain FY26Δ*pal*, we performed western blot analysis of isolated EVs and EV-free extracellular medium generated under the same conditions. The levels of membrane proteins (OmpA, Lpp, and AdhE) were significantly higher in FY26Δ*pal* EVs than in WT FY26 EVs (Fig 6E). Cytoplasmic proteins (CRP and PGK) and ribosomal proteins (RP-S5 and RP-L2) were clearly reduced in the EVs. Similarly, the levels of cytoplasmic proteins (CRP and PGK) and ribosomal proteins (RP-S5 and RP-L2) were clearly reduced in the EV-free extracellular medium relative to their levels in the WT FY26, whereas the membrane proteins (OmpA, Lpp and AdhE) were only detected in ExPEC EVs (Fig 6F). These results suggested that the defect in peptidoglycan crosslinking might not lead to bacterial cell lysis, but predominantly promote the formation of classic OMVs.

### Prophage endolysins induce the formation of ExPEC cytoplasm-carrying EVs

**Fig 7:**
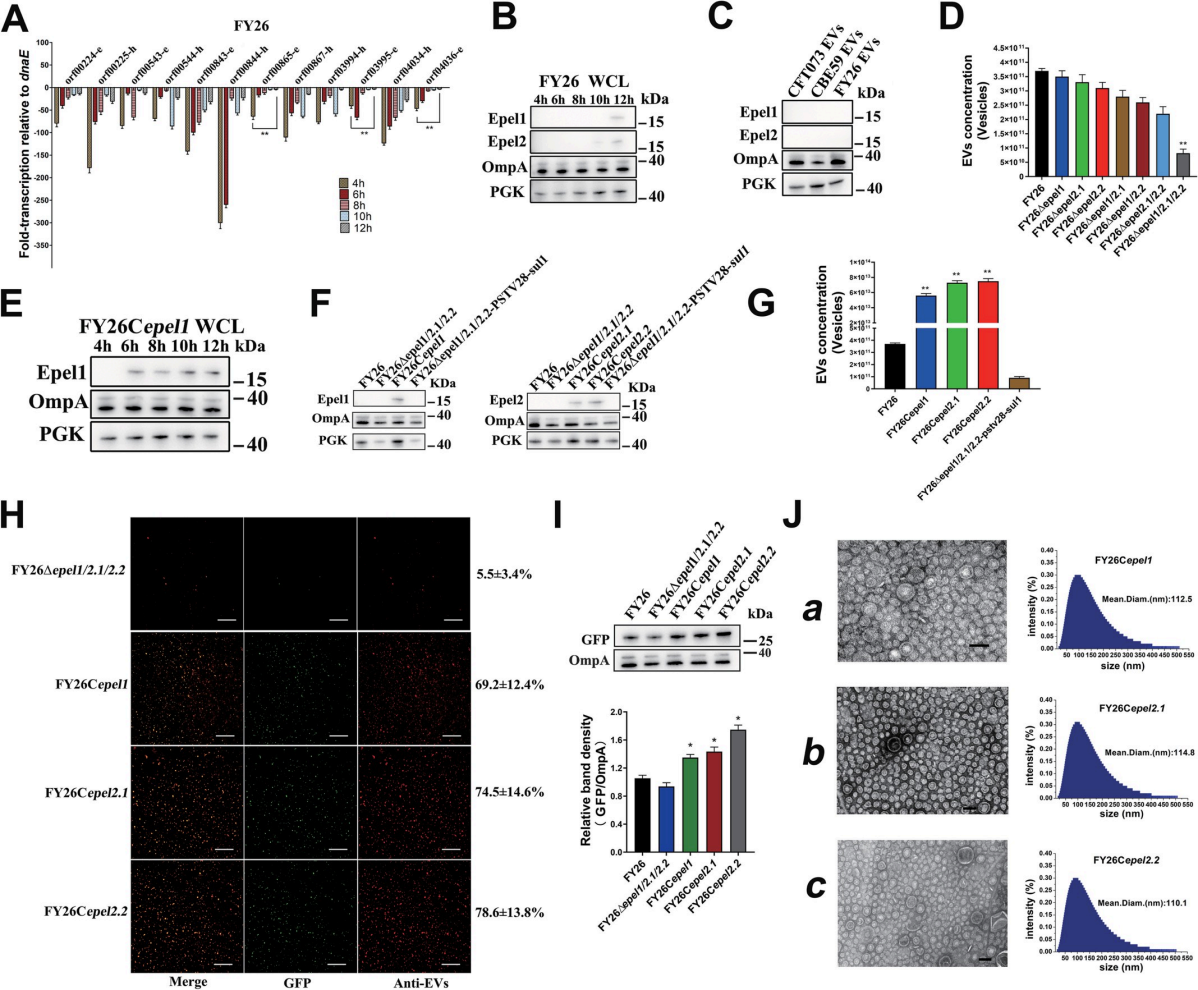
Prophage endolysins induce the formation of ExPEC cytoplasm-carrying vesicles. **(A)** Transcription levels of endolysin genes in ExPEC strains in different growth phases (4, 6, 8, 10, and 12 h) were determined with RT–qPCR.–h, putative holin genes; -e, putative endolysin genes. Data shown are the means ± SEM of three independent experiments relative to the housekeeping gene *dnaE*. Statistical significance was evaluated with one-way ANOVA (**P < 0.01). **(B)** Protein levels of endolysins (Epel1 and Epel2 variants) in whole-cell lysates of ExPEC strains were determined with western blotting. **(C)** Protein levels of endolysins (Epel1 and Epel2 variants) in EVs of ExPEC strains (CBE59, FY26 and CFT073) at 12 h were determined with western blotting. **(D)** The concentrations of purified EVs produced by several mutants were determined with a nanoparticle tracking analysis (NTA). **(E)** Protein levels of Epel1 in complemented strains were determined with western blotting. **(F)** Protein levels of Epel1 and the Epel2 variants in the EVs produced by the complemented strains at 12 h were determined with western blotting. **(G)** The concentrations of purified EVs produced by FY26C*epel1*, FY26C*epel2*.*1*, and FY26C*epel2*.*2* were determined with NTA. **(H)** Immunofluorescent detection of EVs produced by several mutants and complemented strains. **(I)** GFP and OmpA proteins in EVs were determined with western blotting. **(J)** The EVs produced by FY26C*epel1*, FY26C*epel2*.*1*, and FY26C*epel2*.*2* were observed with transmission electron microscopy (TEM). The size distributions of the three purified EVs were detected with Dynamic light scattering (DLS). Scale bars: 200 nm.

In this study, we observed that cytoplasm-carrying vesicles accounted for a specific proportion of the total EVs in various ExPEC strains. Turnbull et al. report that peptidoglycan is degraded by a phage endolysin to induce explosive cell lysis, which is the critical process in the formation of EOMVs and OIMVs in *Pseudomonas aeruginosa* [24]. More than 95% of known bacterial viruses are double-stranded DNA phages, which are widely distributed in the environment [49,50]. Phage-related endolysins degrade the peptidoglycan within the bacterial wall to cause cell lysis [51,52]. Putative prophage genes in the ExPEC genome were predicted with PHAST [53]. The genomic sequences of ExPEC prophages containing putative endolysin genes were aligned (S6A Fig), and the detailed sequence information was shown in S7 Fig. Reverse transcription (RT)-quantitative PCR (qPCR) was used to identify the transcription levels of the endolysin genes and their adjacent holin genes in ExPEC strains at the different stages of culture (S3 Table). Our qPCR primers significantly distinguished the putative endolysin genes. The transcription levels of these genes (orf03995, orf00865, and orf04036) in strain FY26 were clearly enhanced about 12.1-fold, 16.4-fold and 14.7-fold, respectively, in the stationary phase (12h) relative to those in the early log phase of bacterial growth (4h), respectively (*P*≤0.01) (Fig 7A). The transcription levels of the putative endolysin genes in strain CBE59 (orf01633 and orf03205) were clearly enhanced about 19.6-fold and 34.1-fold, respectively, at 12 h relative to those at 4h (*P*≤0.01) (S6Ba Fig). Similarly, the transcription levels of genes AAN80031.1 and AAN81632.1 in strain CFT073 were obviously enhanced about 13.2-fold, and 15.3-fold, respectively, at 12 h relative to those at 4h (*P*≤0.01) (S6Bb Fig). The qPCR results showed that these putative endolysin genes were actively transcribed in the ExPECs, whereas other genes were not. Genes orf00865 and orf04036 in FY26 shared about 89.7% nucleotide homology and 93.8% amino acid homology, suggesting that these genes encoded two endolysin variants. Endolysin gene orf03995 shared low homology with orf00865 and orf04036 in FY26. The genomic alignment showed that the actively transcribed endolysin gene orf00865 in FY26 shared over 90% nucleotide and amino acid homology with AAN81632.1 in CFT073, and that their promoter regions were 100% similar. Similarly, the active endolysin gene orf04036 in FY26 shared over 90% nucleotide and amino acid homology with orf03205 in CBE59, and their promoter regions was 100% similar. The other active endolysin gene in FY26, orf03995, shared strong homology with orf01633 in CBE59 and ANN80031.1 in CFT073, and their promoter regions were 100% similar (S8 Fig). Therefore, the putative endolysin protein encoded by FY26 orf03995 was designated as ExPEC prophage endolysin 1 (Epel1) (accession number JX402062). The endolysin variants encoded by orf00865 and orf04036 in FY26 were designated as Epel2.1 (accession number JX402062) and Epel2.2 (accession number JX402062), respectively. Three putative endolysin genes (*epel1*, *epel2*.*1*, and *epel2*.*2*) were conserved in ExPEC strains CBE59 and CFT073. We had conducted the sequence comparison on the NCBI and Enterobase databases using *epel* genes (*epel1*, *epel2*.*1*, and *epel2*.*2*) sequences with ≥ 95% identity and ≥95% length coverage. The *epel* like genes were widespread among *E*. *coli* strains. Of particular note was that 51.54% sequenced *E*. *coli* strains carried the endolysin gene *epel1*. The *epel2*.*1* was detected in 5.12% *E*. *coli* strains, and about 5.53% *E*. *coli* carried the variant *epel2*.*2*. Although the production of bacteriophages in strains FY26, CBE59, and CFT073 could not be induced, the expression of prophage-related endolysins was detected. The protein levels of endolysins Epel1 and Epel2 were determined in the ExPEC WCLs in the stationary phase (12h) (Figs 7B and S6C). Because the protein levels of endolysins in ExPEC strains and ExPEC EVs were low, western blotting was insufficiently sensitive to detect the expression levels of endolysins in the ExPEC EVs in the stationary phase (12h) (Fig 7C).

To investigate whether endolysins affected the formation of ExPEC EVs, we constructed mutants containing single deletions of the *epel1*, *epel2*.*1*, or *epel2*.*2* gene in strain FY26. The single deletion of these endolysin genes had no effect on production of FY26 EVs (Figs 7D and S6D). We then constructed the double or triple deletion mutants of *epel1*, *epel2*.*1*, and *epel2*.*2* in FY26 with the scarless deletion method [54]. Double deletion of *epel1*/*epel2*.*1*, *epel1*/*epel2*.*2*, or *epel2*.*1*/*epel2*.*2* caused a slight reduction in vesicle production for these mutants relative to that in WT FY26 (Figs 7D and S6D). The triple deletion of *epel1*, *epel2*.*1*, and *epel2*.*2* in the mutant FY26Δ*epel1/2*.*1*/*2*.*2* caused an obvious reduction in vesicle production relative to that in WT FY26 (Figs 7D and S6D). Complementary plasmids containing the endolysin genes (*epel1*, *epel2*.*1*, and *epel2*.*2*) were introduced separately into the triple-deleted mutant FY26Δ*epel1/2*.*1*/*2*.*2* to individually overexpress the endolysin proteins. The levels of the Epel1, Epel2.1, and Epel2.2 proteins in the overexpressing complemented strains at different culture times (4h, 6h, 8h, 10h, and 12h) were determined with western blotting. The protein levels of Epel1, Epel2.1, and Epel2.2 were detected in logarithmic phase (Figs 7E and S6E). Importantly, the endolysin proteins Epel1, Epel2.1, and Epel2.2 were also detected in the EVs produced in complemented strains FY26C*epel1*, FY26C*epel2*.*1*, and FY26C*epel2*.*2* at 12h (Fig 7F). NTA indicated that vesicle production was clearly increased in the complemented strain FY26C*epel1* (151.4-fold) relative to that in WT FY26 (*P*≤0.01) (Figs 7G and S6F). Vesicle production in FY26C*epel2*.*1* and FY26C*epel2*.*2* showed similar trends (197.3-fold and 202.7-fold increases, respectively) (*P* ≤ 0.01) (Figs 7G and S6F). Similarly, a BCA protein assay showed that the concentrations of proteins in the EVs of FY26C*epel1*, FY26C*epel2*.*1*, and FY26C*epel2*.*2* were higher (14.9-fold, 16.4-fold and 17.1-fold, respectively) than that in the EVs of WT FY26 (*P* ≤ 0.01) (S6G Fig). Immunofluorescence assays also showed that the proportions of cytoplasm-carrying vesicles in the complemented strains FY26C*epel1*, FY26C*epel2*.*1*, and FY26C*epel2*.*2* were significantly increased by 69.2±12.4%, 74.5±14.6%, and 78.6±13.8%, respectively (Fig 7H). And the expression levels of GFP protein in the EVs produced by complemented strains FY26C*epel1*, FY26C*epel2*.*1*, and FY26C*epel2*.*2* were determined with western blot. The expression levels of GFP protein were significantly increased in the EVs of complemented strains FY26C*epel1*, FY26C*epel2*.*1*, and FY26C*epel2*.*2* relative to those in the WT FY26 (Fig 7I). TEM images showed that the vesicles produced by the endolysin-complemented strains predominantly consisted of single-layered vesicles, with a few double-bilayer vesicles (OIMVs) (Fig 7J). DLS results showed that the diameter of the EVs produced by FY26C*epel1*, FY26C*epel2*.*1*, and FY26C*epel2*.*2* was about 20–500 nm (Fig 7J). Moreover, the total protein in EVs-free extracellular medium was estimated by BCA in complementary strains. The overexpression of the endolysins in the complemented strains clearly enhanced the amount of total protein in the EV-free extracellular medium. The total protein per liter of bacterial supernatant increased from 3 mg in WT FY26 to > 25mg in the complemented strains (S6H Fig). Cytoplasmic proteins (CRP and PGK) and ribosomal proteins (RP-S5 and RP-L2) were obviously detected in the EV-free extracellular medium (S6I Fig).

These results showed that the overexpression of endolysins in the complemented strains caused significant cell lysis and endolysin-triggered cell death. Moreover, the increased proportion of cytoplasm-carrying vesicles in the EVs of the endolysin-overexpressing strains suggests that bacterial cell lysis promoted the production of ExPEC EVs. The cytoplasm-carrying vesicles observed with immunofluorescence can be considered as EOMVs and OIMVs. ExPEC EVs could be a mixture of OMVs, EOMVs, and OIMVs. TEM images showed that single-layered vesicles were still predominant in the EVs produced by endolysin-overexpressing strains, which means that the numbers of EOMVs and OIMVs in the ExPEC EVs was increased, resulting in endolysin-triggered cell death. Because our electron microscopic detection is limited, we could not accurately calculate the proportions of single-layered and double-layered vesicles among ExPEC EVs.

### SOS response promotes the formation of ExPEC cytoplasm-carrying EVs by activating the expression of prophage endolysins

**Fig 8:**
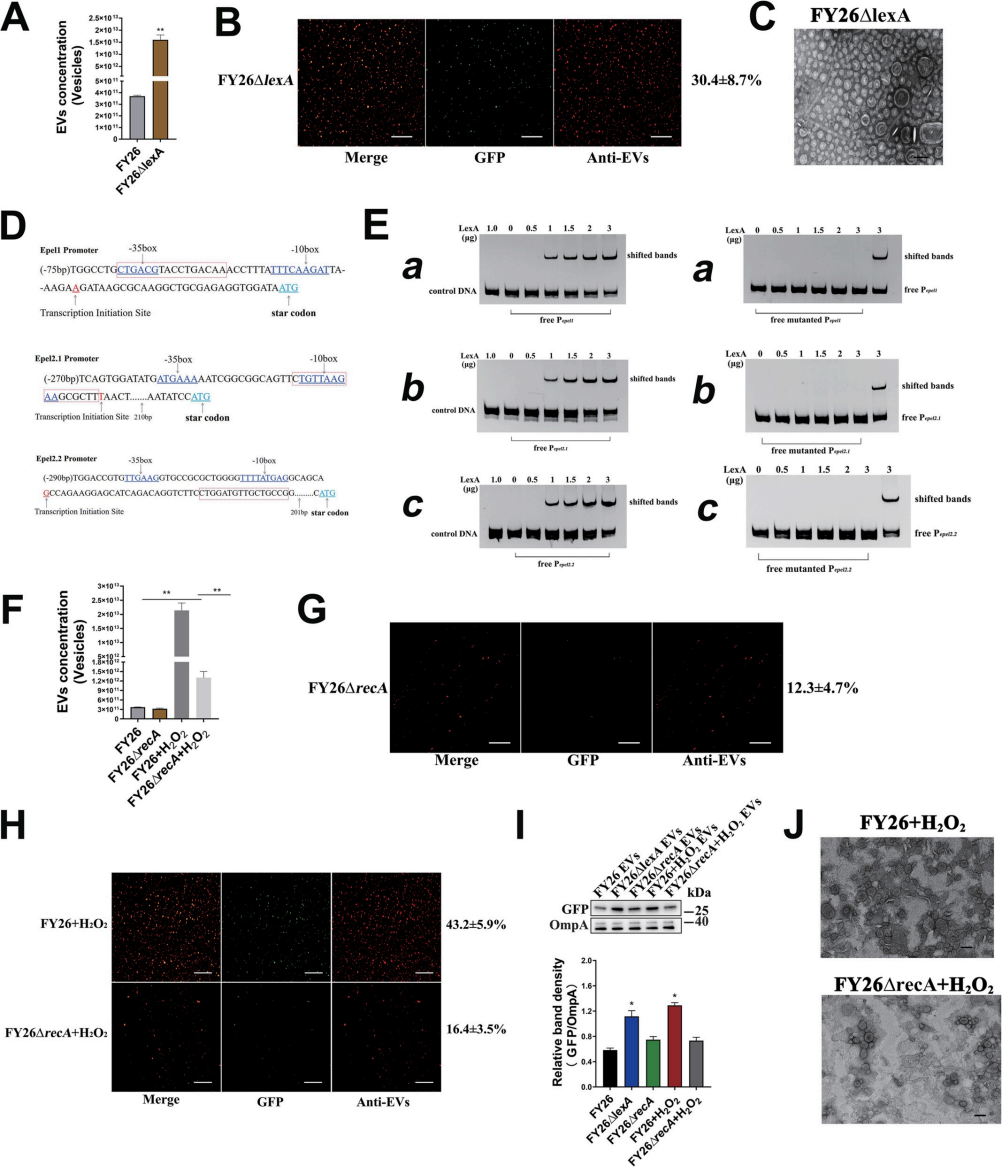
SOS response promotes the formation of ExPEC cytoplasm-carrying vesicles. **(A)** The concentration of purified EVs produced by FY26Δ*lexA* were determined with a nanoparticle tracking analysis (NTA). **(B)** Immunofluorescent staining of EVs produced by FY26Δ*lexA*. **(C)** The EVs produced by FY26Δ*lexA* were observed with transmission electron microscopy (TEM). Scale bars: 200 nm. **(D)** Schematic diagram of transcriptional promoter regions of *epel1*, *epel2*.*1*, and *epel2*.*2* operons. The LexA-binding box is marked in red frame; the corresponding −10 and −35 boxes, the transcription initiation sites, and the start codons of *epel1*, *epel2*.*1*, and *epel2*.*2* are underlined. **(E)** Direct binding of LexA to the promoters of *epel1* **(a)**, *epel2*.*1* **(b)**, and *epel2*.*2* **(c)** was detected with EMSA. DNA fragment (200 bp) of each endolysin gene promoter (*epel1*, *epel2*.*1*, and *epel2*.*2*) containing the LexA-binding site, and the negative control (200 bp) containing the *pal* gene were amplified. Each DNA probe was mixed with an increasing amount of LexA protein for the EMSA analysis. **(F)** The concentrations of purified EVs produced by wild-type (WT) FY26 and mutant FY26Δ*recA* were determined with NTA. The strains were exposed to sublethal concentrations of H_2_O_2_ or cultured under routine conditions. **(G)** Immunofluorescent staining of the EVs produced by FY26Δ*recA*. **(H)** Immunofluorescent detection of EVs produced by FY26 and FY26Δ*recA*. The strains were exposed to sublethal concentrations of H_2_O_2_. **(I)** GFP and OmpA proteins in EVs were determined with western blotting. **(J)** The EVs produced by FY26 and FY26Δ*recA* were observed with transmission electron microscopy (TEM). The strains were exposed to sublethal concentrations of H_2_O_2_. Scale bars: 200 nm.

Two regulatory proteins, RecA and LexA, control the expression of genes associated with the SOS response in *E*. *coli* [55]. LexA acts as a transcriptional repressor, inhibiting the expression of DNA-damage-inducible genes by binding to specific DNA motifs (SOS boxes) located within their promoter regions [56]. In response to DNA damage, RecA binds to single-stranded DNA (ssDNA), which is generated by DNA-damaging agents or other factors that interfere with DNA replication. The binding of RecA to ssDNA activates RecA to stimulate the autocatalytic cleavage of the LexA repressor [57,58]. The cleaved LexA abolishes its binding to SOS boxes, and the expression of the genes involved in the SOS response and DNA repair pathways are derepressed. The LexA regulon not only includes genes related to DNA repair pathways. To determine whether the RecA/LexA-dependant SOS response mediated the formation of ExPEC EVs, mutants in which *lexA* or *recA* were deleted. NTA indicated that the deletion of gene *lexA* led to an obvious increase (43.2-fold) in FY26Δ*lexA* vesicles, compared with the vesicles in WT FY26 at 12h (*P* ≤ 0.01) (Figs 8A and S9A). Similarly, a BCA protein assay showed that the concentration of proteins was higher in FY26Δ*lexA* EVs (9.8-fold) than in WT FY26 (*P*≤0.01) (S9B Fig). Immunofluorescent images showed the increased production (30.4±8.7%) of cytoplasm-carrying vesicles in FY26Δ*lexA* (Fig 8B). And the expression level of GFP protein in the EVs produced by FY26Δ*lexA* was determined with western blot. The levels of GFP protein were significantly increased in the EVs of FY26Δ*lexA* relative to that in the WT FY26 (Fig 8I). Similarly, TEM images showed that the vesicles produced by mutant FY26Δ*lexA* predominantly included single-layered vesicles with a few double-bilayer vesicles (OIMVs) (Fig 8C). The transcription of *epel1*, *epel2*.*1*, and *epel2*.*2* in the mutant FY26Δ*lexA* was determined by qRT-PCR. The transcription levels of *epel1*, *epel2*.*1*, and *epel2*.*2* were clearly increased in FY26Δ*lexA*, about 8.6-fold, 9.4-fold, and 9.7-fold, in the stationary phase (12h) relative to those of WT FY26, respectively (*P*≤0.01) (S9C Fig). The protein levels of the endolysin variants Epel1 and Epel2 were determined in the FY26Δ*lexA* WCL and FY26Δ*lexA* EVs. The Epel1 and Epel2 proteins levels were obviously up-regulated in the FY26Δ*lexA* WCL relative to those in the WT FY26 WCL (S9D Fig). Epel1 and the Epel2.1/Epel2.2 variants were also detected in the EVs of FY26Δ*lexA* in stationary phase (12h) (S9D Fig). These results indicated that the repressor LexA could regulate the protein levels of endolysins, thus influencing the production of ExPEC cytoplasm-carrying EVs.

To block the expression of SOS genes, the LexA protein binds directly to an imperfect palindromic sequence, designated as “LexA box” (an SOS box), located in the promoter regions of SOS genes [59]. The consensus sequence of the SOS box (LexA box) was revealed by analysis of the promoter/operator regions of these *din* genes and other SOS genes known to be regulated by LexA [60]. And outer base pairs (5’-CTG……CAG-3’) was the most consensus bases for the binding sites of *E*. *coli* LexA, which has been identified by a systematic search for operator mutations [61,62]. The base pairs of middle region for LexA box were high variable. And the nearly invariant CTG motif was the canonical LexA recognition sequence [60,63,64]. Furthermore, a large number of LexA boxes have been identified [56,63,65]. To further clarify whether LexA directly regulated the transcription of prophage-related endolysins, the putative *epel* promoter regions were predicted in the FY26 sequence by the online bacteria promoter prediction program BPROM (SoftBerry) [66], and the LexA-binding boxes in the promoter regions of *epel1*, *epel2*.*1*, and *epel2*.*2* in FY26 were predicted. The predicted transcription initiation site of the *epel1* gene was an adenosine (A) at nucleotide (nt) 30 upstream from the start codon (Fig 8D). Inspection of the promoter region of the *epel1* gene revealed a putative LexA-binding box with the sequence 5′- CTGACGTACCTGACAA -3′ between nts −68 and −53 upstream from the start codon of the *epel1* gene. Similarly, the predicted transcription initiation site of *epel2*.*1* was a thymine (T) at nts -222 upstream from the start codon, and a putative LexA-binding box with the sequence 5′- CTGTTAAGAAGCGCTT-3′ occurred between nts −238 and −223 upstream from the *epel2*.*1* start codon (Fig 8D). A putative LexA-binding sequence was also identified in the promoter of the *epel2*.*2* gene. The putative transcription initiation site of *epel2*.*2* was a guanine (G) at nts -246 upstream from the start codon, and a putative LexA-binding box with the sequence 5′- CTGGATGTTGCTGCCG-3′ between nts −219 and −204 upstream from the *epel2*.*2* start codon (Fig 8D). Therefore, we confirmed the direct binding sites for LexA in the promoter DNA of the *epel1*, *epel2*.*1*, and *epel2*.*2* operons with an electromobility shift assay (EMSA). A DNA fragment containing *pal* gene was used as the negative control. LexA protein was successfully expressed and purified (S9E Fig). The EMSA results showed that purified LexA bound to and shifted the promoter DNA of *epel1*, *epel2*.*1*, and *epel2*.*2*. However, no shift in the *pal* fragment was observed (Fig 8E). A DNA fragment with nucleotide mutation in LexA-binding box was also used in an EMSA analysis. No binding or shifted bands was observed for *epel1*, *epel2*.*1*, or *epel2*.*2* without the putative LexA-binding site (Fig 8E). These results confirmed that LexA could bind directly to the promoter regions (SOS boxes) of *epel1*, *epel2*.*1*, and *epel2*.*2* to block the expression of endolysins.

Under routine culture conditions, the deletion of *recA* had no obvious effect on the vesicle production of FY26Δ*recA* relative to that of WT FY26 (Figs 8F and S9A). Similarly, BCA protein assay revealed that deletion of *recA* had no effect on vesicle concentration in FY26Δ*recA* relative to that of WT FY26 (S9F Fig). Immunofluorescent images showed that the deletion of *recA* had no effect on the abundance ratio of cytoplasm-carrying EVs in the total EVs of FY26 (Fig 8G). And the expression level of GFP protein in the EVs produced by FY26Δ*recA* was determined with western blot. The level of GFP protein in the EVs of FY26Δ*recA* was similar to that in the WT FY26 (Fig 8I). The transcription of *epel1*, *epel2*.*1*, and *epel2*.*2* was not significantly different in FY26Δ*recA* from that in WT FY26 in the stationary phase (12h) (S9G Fig). Hydrogen peroxide (H_2_O_2_) causes oxidative stress in microorganisms and damages the bacterial structure by generating reactive oxygen species (ROS). H_2_O_2_ acts as a debriding agent with an outstanding antibacterial effect. As H_2_O_2_-induced oxidative damage worsens, apoptosis-like death occurs in *E*. *coli* [67]. The SOS response in *E*. *coli* can be triggered by H_2_O_2_ exposure, accelerating the oxidation of bacterial DNA [68]. Similar to the calculation of the minimum inhibitory concentration (MIC) of an antibiotic, we calculated the sublethal concentration of H_2_O_2_ when WT FY26 was cultured with H_2_O_2_. We also compared the number of vesicles in WT FY26 and mutant FY26Δ*recA* after exposure to H_2_O_2_. The sublethal concentration of H_2_O_2_ (1.0 mmol/L) was added to each culture at time zero, and H_2_O_2_ was added to the culture when the OD_600_ value was 0.3. Then H_2_O_2_ was added to the medium at 3 h intervals to protect the cells from degradation. After incubation for 12h, the EVs were isolated and the numbers of vesicles released by these two strains were determined. NTA showed that the deletion of *recA* significantly reduced the number of vesicles produced by FY26Δ*recA* relative to the number in WT FY26 (*P* ≤ 0.01) (Figs 8F and S9H). Similarly, BCA protein assay revealed that the concentration of proteins in FY26Δ*recA* EVs was decreased (2.7-fold) compared to that of WT FY26 (*P*≤0.01) (S9F Fig). The protein levels of the endolysin variants Epel1 and Epel2 were detected in the WCLs and EVs. The protein levels of the endolysins were increased when WT FY26 was exposed to the sublethal concentration of H_2_O_2_ (S9I Fig). The protein levels of the endolysins were also determined in the EVs of WT FY26 (S9I Fig). However, the protein levels of endolysins were not obviously upregulated in the mutant FY26Δ*recA* (S9I Fig). Immunofluorescence assays showed that the proportion of cytoplasm-carrying EVs in the total EVs of FY26 increased significant by 43.2±5.9% when FY26 was exposed to H_2_O_2_ (Fig 8H). And the expression level of GFP protein in the EVs of FY26 cultured with sublethal concentration of H_2_O_2_ was determined with western blot. The level of GFP protein was increased in the EVs when WT FY26 was exposed to the sublethal concentration of H_2_O_2_ (Fig 8I). However, the deletion of *recA* reduced the production of cytoplasm-carrying EVs in FY26Δ*recA* exposed to H_2_O_2_ relative to those in H_2_O_2_-exposed WT FY26 (*P*≤0.01) (Fig 8H). And the level of GFP protein was decreased in the EVs when WT FY26Δ*recA* was exposed to the sublethal concentration of H_2_O_2_ (Fig 8I). TEM images also revealed that single-layered vesicles (EOMVs) were present in relatively large numbers when WT FY26 was exposed to H_2_O_2_ (Fig 8J). Taken together, these results showed that the RecA/LexA-dependent SOS response mediated the formation of ExPEC cytoplasm-carrying EVs by inducing the expression of endolysins. The H_2_O_2_-induced SOS response activated endolysin-triggered cell lysis, producing the cytoplasm-carrying EVs.

To investigate whether the SOS response only acted through the expression of endolysin, stimulating the production of cytoplasm-carrying EVs, the mutant FY26Δ*epel1*/*2*.*1*/*2*.*2* was cultured in medium supplemented with H_2_O_2_. After incubation for 12h, the EVs were isolated and the number of vesicles produced by the mutant FY26Δ*epel1*/*2*.*1*/*2*.*2* was determined. The results showed that the EVs production by the mutant FY26Δ*epel1*/*2*.*1*/*2*.*2* incubated with H_2_O_2_ was close to that of the same mutant cultured under routine conditions (S9J Fig). BCA protein assay revealed that the EVs production by the mutant FY26Δ*epel1*/*2*.*1*/*2*.*2* incubated with H_2_O_2_ was close to that of the same mutant cultured under routine conditions (S9K Fig). The increase in endolysin expression was clearly dependent upon the activation of the SOS response.

### Deletion of *ftsK* gene induces the expression of endolysins, increasing the production of ExPEC cytoplasm-carrying EVs

**Fig 9:**
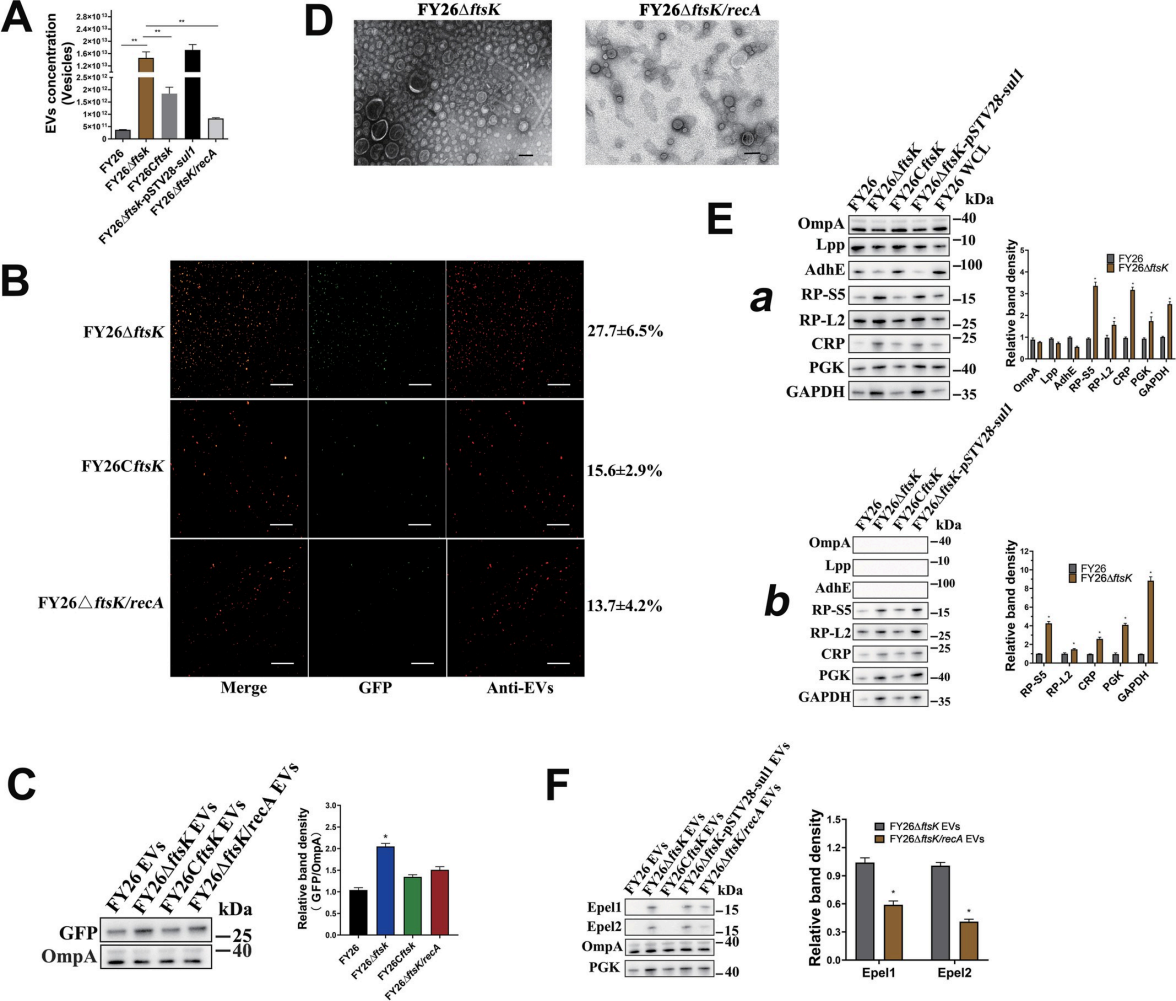
Deletion of *ftsK* gene increased the production of ExPEC cytoplasm-carrying vesicles. **(A)** The concentrations of EVs purified from FY26Δ*ftsK*, FY26C*ftsK* and FY26Δ*ftsK*/*recA* were determined with a nanoparticle tracking analysis (NTA). **(B)** Immunofluorescent staining of EVs produced by FY26Δ*ftsK*, FY26C*ftsK* and FY26Δ*ftsK*/*recA*. Percentage of cytoplasm-carrying vesicles (green fluorescence) among the total EVs (red fluorescence) is indicated on the right. Graph shows a representative image. Scale bars: 10 μm. **(C)** GFP and OmpA proteins in EVs were determined with western blotting. **(D)** The EVs produced by FY26Δ*ftsK* and FY26Δ*ftsK*/*recA* were observed with transmission electron microscopy (TEM). Scale bars: 200 nm. **(E)** Levels of membrane and cytoplasmic proteins in EVs **(a)** and EV-free extracellular medium **(b)** from FY26Δ*ftsK* and FY26C*ftsK* were determined with western blotting. **(F)** Protein levels of endolysins Epel1 and Epel2 variants in EVs of FY26Δ*ftsK* and FY26Δ*ftsK*/*recA* were determined with western blotting.

The multi-spanning membrane protein FtsK plays critical roles in *E*. *coli* cell division. The N-terminal membrane domain of FtsK mediates cell division, whereas the C-terminal membrane domain is involved in the subsequent chromosomal segregation process [69]. The deletion of the *ftsK* gene in *E*. *coli* suppresses the division phenotype and causes a temperature-sensitive phenotype with clearly slow growth at low (30°C) and high (42°C) temperatures [69]. To determine the impact of *ftsK* deletion on the production of ExPEC EVs, the mutant FY26Δ*ftsK* and complemented strain FY26C*ftsK* were generated. NTA showed that the vesicle production of FY26Δ*ftsK* increased 39.5-fold relative to that of WT FY26 cultured at 37°C for 12h (*P*≤0.01) (Figs 9A and S10A), and that the vesicle production of the complemented strain FY26C*ftsK* was clearly reduced and approached to the original level of WT FY26. BCA protein assay revealed that the concentration of proteins in FY26Δ*ftsK* EVs was increased (8.7-fold) compared to that of WT FY26 (*P*≤0.01) (S10B Fig). Notably, immunofluorescence assays showed a significant increase (27.7±6.5%) in the proportion of cytoplasm-carrying vesicles in the mutant FY26Δ*ftsK* (Fig 9B). And the expression level of GFP protein in the EVs produced by FY26Δ *ftsK* was determined with western blot. The level of GFP protein was significantly increased in the EVs of FY26Δ *ftsK* relative to that in the WT FY26 (Fig 9C). The vesicle types in the EVs produced by the mutant FY26Δ*ftsK* were identified with TEM (Fig 9D). Western blotting was used to investigate the protein levels in the EVs and EV-free extracellular medium when the same amount of total protein was loaded (5.0 μg). Compared with WT FY26, the levels of membrane proteins (OmpA, Lpp and AdhE) in the EVs of FY26Δ*ftsK* were reduced, whereas those of cytoplasmic proteins (CRP and PGK) and ribosomal proteins (RP-S5 and RP-L2) were clearly increased in the EVs (Fig 9Ea). Similarly, the levels of cytoplasmic proteins (CRP and PGK) and ribosomal proteins (RP-S5 and RP-L2) were clearly higher in the EVe-free extracellular medium of FY26Δ*ftsK*, compared with same loading amount of extracellular medium protein of WT FY26 (*P* ≤ 0.01) (Fig 9Eb). It has been reported that the repression of the FtsK function in *E*. *coli* induces the induction of the SOS response [69]. The double deletion of the *ftsK* and *recA* genes significantly reduced (17.6-fold) the number of vesicles produced in the mutant FY26Δ*ftsK*/*recA* relative to the number produced in FY26Δ*ftsK* (*P*≤0.01) (Fig 9A). BCA protein assay revealed that the concentration of proteins in FY26Δ*ftsK/recA* EVs was decreased (2.1-fold) compared to that of FY26Δ*ftsK* (*P*≤0.01) (S10B Fig). The immunofluorescence assays revealed the proportion of cytoplasm-carrying vesicles in FY26Δ*ftsK*/*recA* was 13.7±4.2%, approaching the original level in WT FY26 (Fig 9B). The expression level of GFP protein in the EVs of FY26Δ*ftsK*/*recA* was similar to that in the WT FY26 (Fig 9C). TEM images showed that the vesicles produced by mutant FY26Δ*ftsK*/*recA* predominantly included single-layered vesicles (Fig 9D). The transcription of *epel1*, *epel2*.*1*, and *epel2*.*2* in the mutant FY26Δ*ftsK* was determined with qRT-PCR. The results indicated that the transcription levels of *epel1*, *epel2*.*1*, and *epel2*.*2* in FY26Δ*ftsK* were clearly increased about 7.9-fold, 8.6-fold, and 8.9-fold, respectively, at 12 h (*P*≤0.01) (S10C Fig). However, the transcription levels of these endolysin genes were significantly decreased in FY26Δ*ftsK*/*recA* (S10C Fig). The protein levels of the endolysins were also determined in the FY26Δ*ftsK* WCLs and EVs. The levels of Epel1 and Epel2 proteins in the FY26Δ*ftsK* WCLs were higher than those in the WT FY26 WCLs (S10D Fig). The protein levels of the endolysins in the FY26Δ*ftsK*/*recA* approached the original levels in WT FY26 (S10D Fig). Epel1 and Epel2.1/Epel2.2 variants were also detected in the EVs of FY26Δ*ftsK* at 12h, but their protein levels in the EVs of FY26Δ*ftsK*/*recA* were significantly lower than those in FY26Δ*ftsK* (*P* ≤ 0.05) (Fig 9F). These results indicated that the deletion of *ftsK* induced endolysin-triggered cell lysis, increasing the production of ExPEC cytoplasm-carrying EVs through the RecA/LexA-dependent SOS response.

### Defect in t^6^A synthesis in ExPEC enhanced the production of cytoplasm-carrying EVs through the RecA/LexA-dependent SOS response

**Fig 10:**
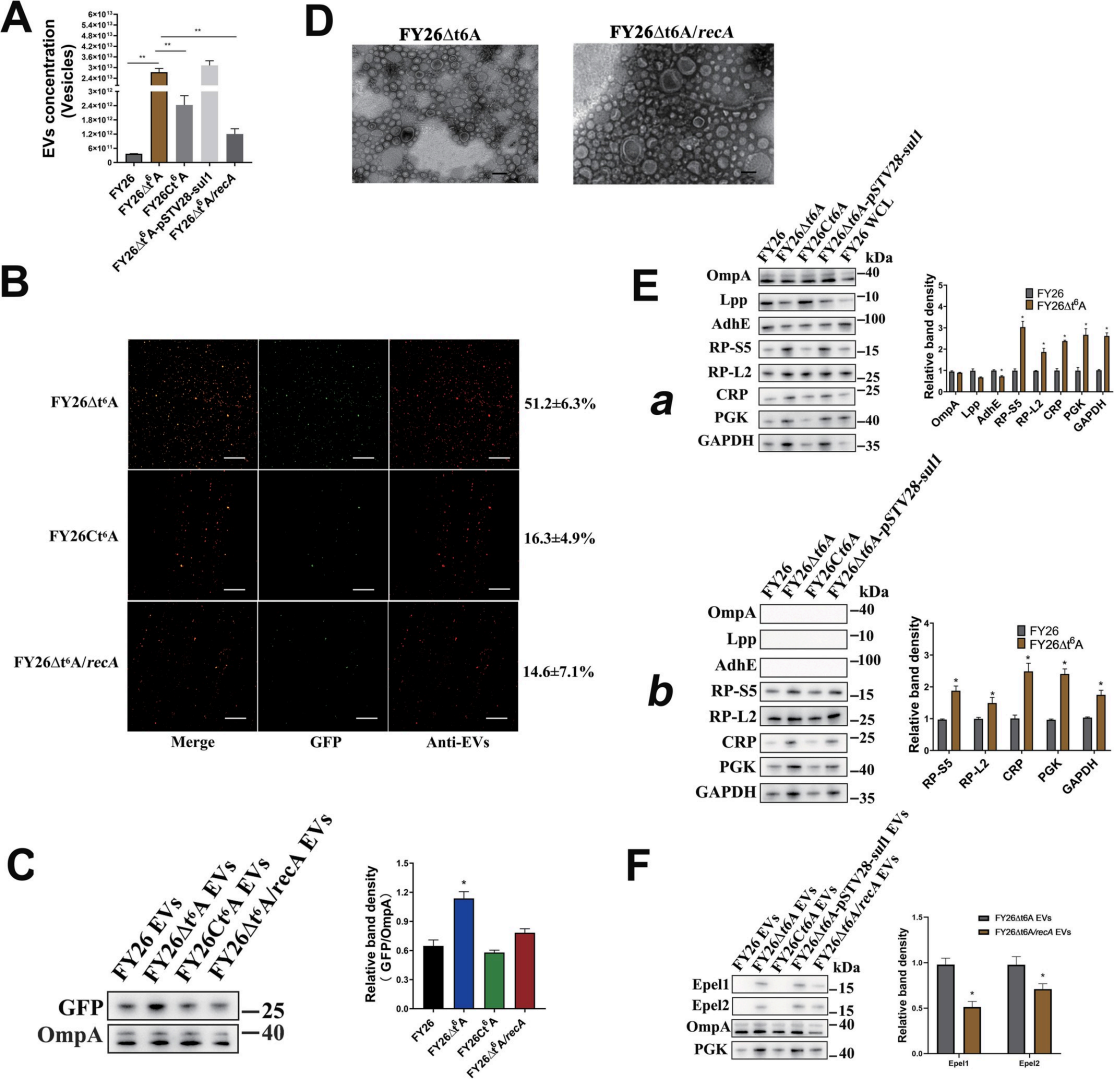
Absence of t^6^A synthesis genes in ExPEC enhanced the production of cytoplasm-carrying vesicles. **(A)** The concentrations of EVs purified from FY26Δt6A, FY26Ct6A and FY26Δt6A/*recA* were determined with a nanoparticle tracking analysis (NTA). **(B)** Immunofluorescent staining of EVs produced by FY26Δt6A, FY26Ct6A and FY26Δt6A/*recA*. Percentage of cytoplasm-carrying vesicles (green fluorescence) among the total EVs (red fluorescence) is indicated on the right. Graph shows a representative image. Scale bars: 10 μm. **(C)** GFP and OmpA proteins in EVs were determined with western blotting. **(D)** The EVs produced by FY26Δt6A and FY26Δt6A/*recA* were observed with transmission electron microscopy (TEM). Scale bars: 200 nm. **(E)** Levels of membrane and cytoplasmic proteins in EVs **(a)** and EV-free extracellular medium **(b)** from FY26Δt6A and FY26Ct6A were determined with western blotting. **(F)** Protein levels of endolysins Epel1 and Epel2 variants in EVs of FY26Δt6A and FY26Δt6A/*recA* were determined with western blotting.

The maturation of tRNA usually involves numerous post-transcriptional modifications, which are necessary for the function of tRNAs, especially ensuring the accuracy of translation [70]. N^6^-threonyl-carbamoyl adenosine (t^6^A) is a universal modification in most tRNAs decoding ANN codons, and is conserved at position 37 of the anticodon loop [71]. The YgjD, YjeE, YeaZ, and YrdC proteins are essential for the biosynthesis of t^6^A molecules in *E*. *coli* [72]. To investigate the effects of the disruption of t6A synthesis on the production of ExPEC EVs, the mutant FY26Δt6A was generated by the deletion of the essential gene *ygjD*. NTA showed that the number of vesicles produced by FY26Δt6A clearly increased 74.1-fold relative to that in WT FY26 after 12 h in culture (*P* ≤ 0.01) (Figs 10A and S11A), and that the vesicle production of the complemented strain FY26Ct6A was significantly lower than that of FY26Δt6A. The concentration of proteins in FY26Δt6A EVs was increased (11.7-fold) compared to that of WT FY26 (*P*≤0.01) (S11B Fig). Notably, immunofluorescence indicated a significant increase (51.2±6.3%) in the production of cytoplasm-carrying vesicles in the mutant FY26Δt6A relative to that in WT FY26 (Fig 10B). And the expression level of GFP protein in the EVs produced by FY26Δt6A was determined with western blot. The level of GFP protein was significantly increased in the EVs of FY26Δt6A relative to that in the WT FY26 (Fig 10C). TEM images of the EVs produced by the mutant FY26Δt6A revealed that single-layered vesicles (EOMVs) still accounted for a relatively large number of EVs (Fig 10D). The levels of EV-associated proteins in the EVs and EV-free supernatant of the mutant FY26Δt6A were determined by western blotting. Similar to the results of *ftsK* deletion, the levels of membrane proteins (OmpA, Lpp, and AdhE) were reduced in the EVs of the mutant FY26Δt6A, whereas the levels of cytoplasmic and ribosomal proteins were clearly increased compared with those in the WT FY26 EVs (Fig 10Ea). The levels of cytoplasmic and ribosomal proteins were clearly higher in EV-free supernatant of the mutant FY26Δt6A than in that of WT FY26, whereas the membrane proteins were mainly detected in the ExPEC EVs (Fig 10Eb).

The double deletion of the *ygjD* and *recA* genes in the mutant FY26Δt6A/*recA* caused an obvious reduction (22.8-fold) in the number of the vesicles relative to the number in FY26Δt6A after 12 h in culture (*P* ≤ 0.01) (Figs 10A and S11A). BCA showed the concentration of proteins in FY26Δt6A*/recA* EVs was decreased (2.4-fold) compared to that of WT FY26 (*P*≤0.01) (S11B Fig). The immunofluorescence assays revealed the proportion of cytoplasm-carrying vesicles in FY26Δt6A/*recA* was 14.6±7.1%, approaching the original level in WT FY26 (Fig 10B). The expression level of GFP protein in the EVs of FY26Δt6A/*recA* was similar to those in the WT FY26 (Fig 10C). TEM images showed that the vesicles produced by mutant FY26Δt6A/*recA* predominantly included single-layered vesicles (Fig 10D). The transcription levels of *epel1*, *epel2*.*1*, and *epel2*.*2* in FY26Δt6A were clearly increased (about 13.7-fold, 15.8-fold, and 16.2-fold, respectively) at 12h relative to those in WT FY26 (*P* ≤ 0.01) (S11C Fig). On the contrary, the transcription levels of these endolysin genes in FY26Δt6A/*recA* were significantly lower than those in FY26Δt6A (S11C Fig). Epel1 and Epel2 proteins were up-regulated in FY26Δt6A WCLs compared with their expression in WT FY26 (S11D Fig). The protein levels of endolysins in FY26Δt6A/*recA* were approached the original levels in WT FY26 (S11D Fig). Epel1 and the Epel2.1/Epel2.2 variants were detected in the EVs of FY26Δt6A at 12 h, but their protein levels in the EVs of FY26Δt6A/*recA* were significantly reduced (*P* ≤ 0.01) relative to those in FY26Δt6A (Fig 10F). A previous study demonstrated that the deletion of t6A synthesis genes caused unbalanced cell division, resulting in cells with elongated phenotypes and abnormal DNA distributions [73]. Similar to the induction of the SOS response after *ftsK* deletion, we showed that the deletion of *ygjD* gene, essential for t6A synthesis, also induced the RecA/LexA-dependent SOS response, regulating the expression of endolysins. The prophage endolysins were essential for the formation of cytoplasm-carrying vesicles in the ExPEC *ftsK* or t^6^A mutants. The absence of t^6^A significantly affects the growth rate of *E*. *coli* and weakens its ability to tolerate stress. Although the growth rate of FY26Δt6A was significantly reduced, its EV production was unaffected, and its content of cytoplasm-carrying vesicles increased significantly.

### Proportion of cytoplasm-carrying EVs affects the DNA content carried by ExPEC EVs

**Fig 11:**
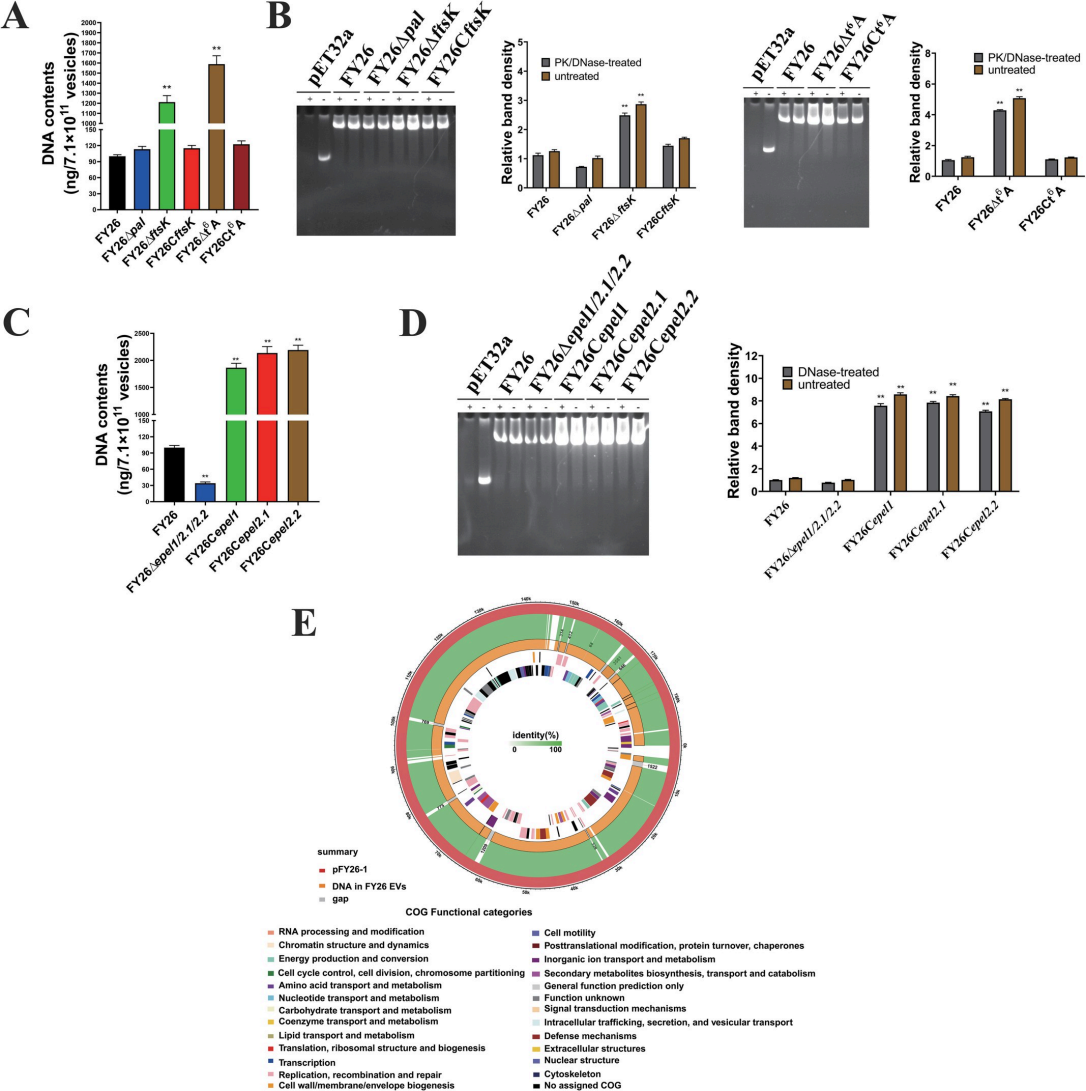
Proportion of cytoplasm-carrying vesicles affects the DNA content carried by ExPEC EVs. **(A)** Total DNA in EVs was measured with microplate reader. Total DNA was extracted from equivalent numbers of EVs (7.1 × 10^11^ vesicles) from FY26, FY26Δ*pal*, FY26Δ*ftsK*, FY26C*ftsK*, FY26Δt6A, and FY26Ct6A were measured with microplate reader. Data were obtained from at least three independent experiments, with three replicates. Statistical significance was evaluated with one-way ANOVA (**P < 0.01). **(B)** Total DNA in the EVs (FY26, FY26Δ*pal*, FY26Δ*ftsK*, FY26C*ftsK*, FY26Δt6A, and FY26Ct6A) was visualized with nondenaturing polyacrylamide gel electrophoresis. Total DNA was isolated from equivalent numbers (7.1 × 10^11^) of PK/DNase-treated and untreated EVs from wild-type (WT) strain FY26 and several mutants. ‘+’ indicates that samples were treated with PK and DNaseI, and ‘−’ indicates that samples were not treated with PK or DNaseI. Naked DNA (pET-32a) was used as a control. **(C)** Total DNA in EVs was measured with microplate reader. Total DNA was extracted from equivalent numbers of EVs (7.1 × 10^11^ vesicles) from WT FY26, triple-deleted mutant FY26Δ*epel1*/*2*.*1*/*2*.*2*, and endolysin-overexpressing complemented strains FY26C*epel1*, FY26C*epel2*.*1*, and FY26C*epel2*.*2*, and measured with microplate reader. **(D)** Total DNA in EVs was visualized with nondenaturing polyacrylamide gel electrophoresis. Total DNA was extracted from equivalent numbers of EVs (7.1 × 10^11^ vesicles) from WT FY26, triple-deleted mutant FY26Δ*epel1*/*2*.*1*/*2*.*2*, and endolysin-overexpressing complemented strains FY26C*epel1*, FY26C*epel2*.*1*, and FY26C*epel2*.*2*, and analyzed with nondenaturing polyacrylamide gel electrophoresis. Naked DNA (pET-32a) was used as a control. **(E)** Circular maps of the complete plasmid sequence carried by FY26 EVs. FY26 contained one large plasmid pFY26-1. Circles from outer to inner show: pFY26-1 plasmid sequence (ring 1, red), homologous alignment (ring 2, green), DNA in FY26 EV (ring 3, orange), genes on the forward strand, colored according to COG classification (ring 4), and genes on the reverse strand, colored according to COG classification (ring 5).

The total DNA in PK and DNase-treated EVs was isolated from WT FY26 and several mutants to measure the DNA content in the EV cargoes. When WT FY26 was cultured for 12h, about 7.1×10^11^ EV, contained 100 ng of DNA. There was no obvious difference in the DNA content of an equivalent number of FY26Δ*pal* EVs (Fig 11A). However, the deletion of *ftsK* led to an increase in the DNA content of the EVs. The DNA content in FY26Δ*ftsK* EVs was 1,212 ng, which was 12.1-fold more than in the EVs of WT FY26 (*P* ≤ 0.01) (Fig 11A). Like the FY26Δ*ftsK* EVs, the FY26Δt6A EVs contained significantly more DNA cargo than the EVs of WT FY26 (Fig 11A). We also determined the total DNA in the EVs of these mutants with non-denaturing polyacrylamide gel electrophoresis. At an equivalent concentration of vesicles (7.1×10^11^), more DNA was detected in the EVs from mutants FY26Δ*ftsK* and FY26Δt6A than in the WT FY26 EVs (Fig 11B).

To determine whether the abundance ratio of cytoplasm-carrying vesicles affects the DNA content of the ExPEC EVs, we measured the DNA content in EVs from WT FY26, triple-deleted mutant FY26Δ*epel1*/*2*.*1*/*2*.*2*, and endolysin-overexpressing complemented strains FY26C*epel1*, FY26C*epel2*.*1*, and FY26C*epel2*.*2* cultured for 12 h. As result, at an equivalent concentration (7.1×10^11^ vesicles), the complementary strains FY26C*epel1* carried 1,864.4 ng of DNA and held 18.6-fold higher than that of WT FY26 (*P*≤0.01) (Fig 11C). The DNA content in the EVs of complemented strains FY26C*epel2*.*1* and FY26C*epel2*.*2* showed similar trends relative to that in WT FY26 EVs (both *P* ≤ 0.01) (Fig 11C). However, 7.1×10^11^ EVs from the triple-deleted mutant FY26Δ*epel1*/*2*.*1*/*2*.*2* contained only 34ng DNA. Moreover, we determined the total DNA in the EVs of these strains with non-denaturing polyacrylamide gel electrophoresis. At an equivalent EV concentration (7.1×10^11^ vesicles/mL), more DNA was also detected in the EVs of endolysin-overexpressing complemented strains than in WT FY26 EVs (Fig 11D). We also analyzed the DNA in the FY26 EVs with whole-genome sequencing. The circular map showed that the FY26 EVs carried their DNA in a large plasmid pFY26-1 (GenBank: CP101742, Fig 11E). These results demonstrated that the DNA cargo carried by ExPEC EVs was consistent with the abundance ratio of cytoplasm-carrying vesicles.

### Antibiotics enhance the production of ExPEC cytoplasm-carrying EVs

**Fig 12:**
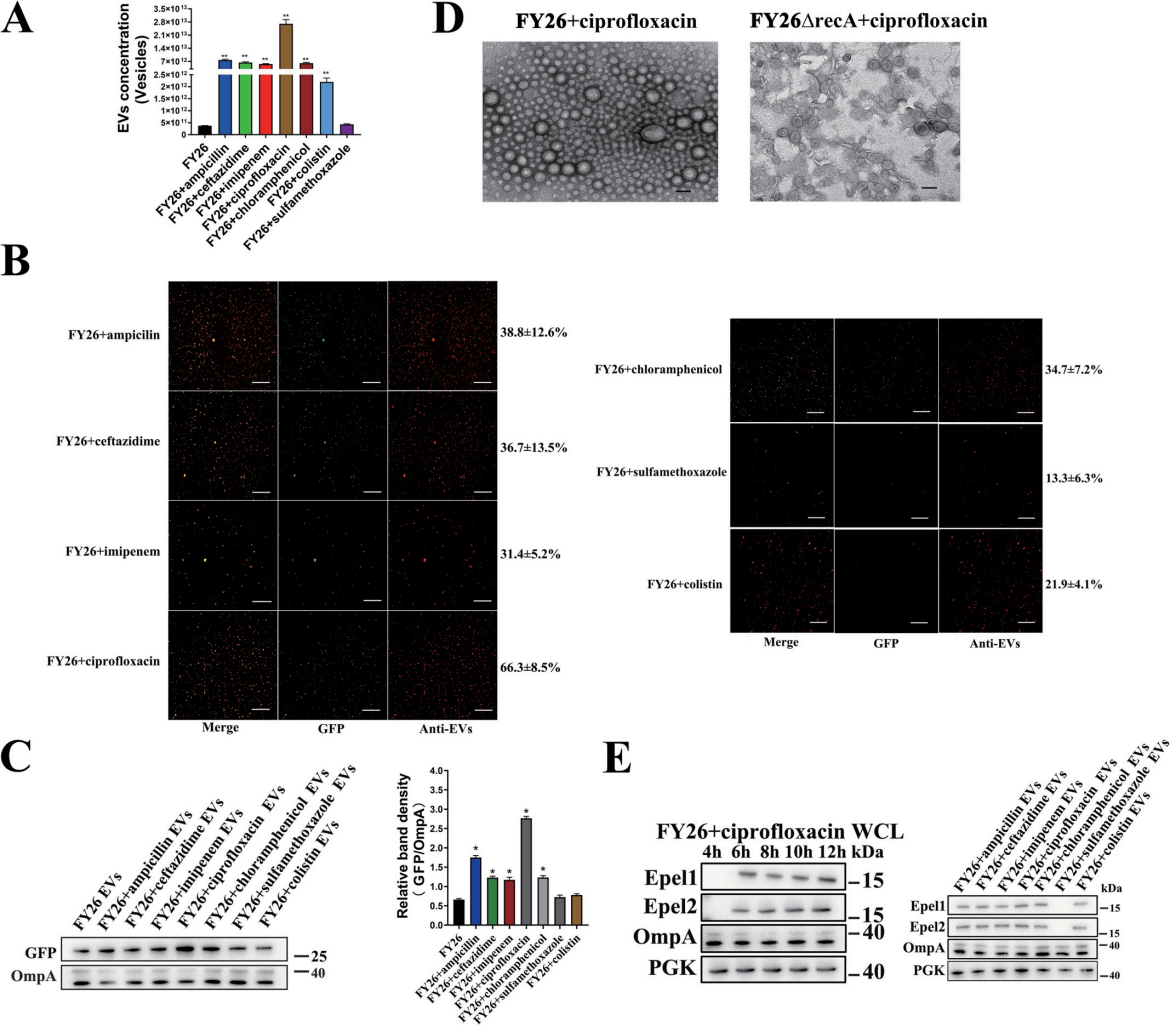
Antibiotics enhance the production of ExPEC EVs. **(A)** The concentrations of purified EVs in FY26 cultured with sublethal concentrations of antibiotics were determined with a nanoparticle tracking analysis (NTA). FY26 strain was treated with sublethal doses of seven antibiotics (ampicillin, 2 μg/mL; ceftazidime, 0.3 μg/mL; imipenem, 2.5 μg/mL; ciprofloxacin, 0.3 μg/mL; chloramphenicol, 2 μg/mL; colistin, 0.3 μg/mL, sulfamethoxazole, 2 μg/mL). **(B)** Immunofluorescent staining of EVs produced by FY26 strains treated with sublethal concentrations of antibiotics. Proportion of cytoplasm-carrying vesicles is indicated on the right. **(C)** GFP and OmpA proteins in EVs were determined with western blotting. **(D)** The purified EVs in FY26 cultured with sublethal concentrations of antibiotics were observed with transmission electron microscopy (TEM). Scale bars: 200 nm. **(E)** Protein levels of endolysins Epel1 and the Epel2 variants in whole-cell lysates (WCLs) and EVs of FY26 strain cultured with sublethal concentrations of antibiotics were determined with western blotting.

To determine the effects of antibiotics on the production of ExPEC EVs, the medium was supplemented with sublethal doses of seven antibiotics (ampicillin, ceftazidime, imipenem, ciprofloxacin, chloramphenicol, sulfamethoxazole, or colistin) that have different antibacterial and bactericidal mechanisms. NTA showed that the incubation of WT FY26 with a sublethal dose of specific antibiotics caused an increase in vesicle production after 12 h (ampicillin, 21.1-fold; ceftazidime, 17.3-fold; imipenem, 15.1-fold; ciprofloxacin, 72.9-fold; chloramphenicol, 16.5-fold; colistin, 5.9-fold), compared with the EV production in FY26 cultured without antibiotics (*P* ≤ 0.01) (Figs 12A and S12A). However, there was no significant change in FY26 cultured with a sublethal concentration of sulfamethoxazole (Figs 12A and S12A). Similarly, a BCA protein assay showed that the concentration of EVs proteins when FY26 was exposed to sublethal level of ciprofloxacin was increased (13.2-fold) compared to the concentration in FY26 cultured without antibiotics (*P* ≤ 0.01) (S13A Fig). Immunofluorescence microscopy also revealed that the abundance ratio of cytoplasm-carrying vesicles increased in FY26 cultured with sublethal concentrations of antibiotics (ampicillin, 38.8±12.6%; ceftazidime, 36.7±13.5%; imipenem, 31.4±5.2%; ciprofloxacin, 66.3±8.5%; chloramphenicol, 34.7±7.2%; colistin, 21.9±4.1%) compared with that in FY26 cultured without antibiotics (Fig 12B), suggesting that endolysin-triggered cell lysis had occurred. And the expression level of GFP protein in the EVs when FY26 was exposed to sublethal level of antibiotics was determined with western blot. The expression levels of GFP proteins were increased in the EVs when WT FY26 was exposed to the sublethal concentration of antibiotics (Fig 12C). TEM images showed that the increased vesicles were predominantly single-layered vesicles (EOMVs) with a few double-bilayer vesicles (OIMVs) when FY26 was exposed to sublethal level of ciprofloxacin (Fig 12D). To investigate whether the expression of endolysins was affected by antibiotics, the transcriptional levels of *epel1*, *epel2*.*1*, and *epel2*.*2* when FY26 was incubated with sublethal concentration of ciprofloxacin or sulfamethoxazole were detected with qRT-PCR. The transcription levels of *epel1*, *epel2*.*1*, and *epel2*.*2* were clearly increased (15.16-fold, 16.69-fold, and 17.32-fold) cultured at 12h compared with their levels in FY26 cultured without antibiotics (S13B Fig). However, there was no significant change in FY26 cultured with sulfamethoxazole (S13B Fig). The protein levels of Epel1 and Epel2 in WT FY26 cultured with sub-lethal concentrations of antibiotics were measured, and the results were consistent with qRT-PCR results (Figs 12E and S13C). The levels of Epel1 and Epel2 proteins were increased in the EVs of WT FY26 cultured with various antibiotics, except sulfamethoxazole (Fig 12E).

To evaluate whether antibiotics mediated the formation of cytoplasm-carrying vesicles by inducing the SOS response, the FY26Δ*recA* strains was cultured in medium with sub-lethal doses of various antibiotics, and several antibiotics reduced vesicle production (ampicillin, 2.8-fold; ceftazidime, 2.2-fold; imipenem, 2.4-fold; ciprofloxacin, 51.9-fold; chloramphenicol, 9.7-fold; colistin, 2.1-fold), compared to that of WT FY26 cultured with the same antibiotics (*P*≤0.05) (S13D and S12B Figs). The EV production of FY26Δ*recA* incubated with ciprofloxacin was clearly reduced and was similar to the production of EVs by WT FY26 under routine culture conditions. However, the EV production of FY26Δ*recA* incubated with *β*-lactam antibiotics (penicillins, ceftazidime, and carbapenems), chloramphenicol or colistin was higher than of WT FY26 under routine culture conditions, although immunofluorescence microscopy revealed that the abundance ratio of cytoplasm-carrying vesicles was significantly reduced relative to that of WT FY26 incubated with antibiotics (S13E Fig). Similarly, a BCA protein assay showed that the concentration of EVs proteins when FY26Δ*recA* was exposed to sublethal level of ciprofloxacin was decreased (7.4-fold) compared to the concentration in FY26 cultured with ciprofloxacin antibiotics (*P* ≤ 0.01) (S13A Fig). And the expression levels of GFP proteins in the EVs when FY26Δ*recA* was exposed to sublethal level of antibiotics were determined with western blot. The result indicated that the proportion of cytoplasm-carrying EVs produced by FY26Δ*recA* incubated with antibiotics was consistent with the expression level of GFP protein (S13F Fig). TEM images showed that the vesicles were predominantly single-layered vesicles (EOMVs) with a few double-bilayer vesicles (OIMVs) when FY26Δ*recA* was exposed to sublethal level of ciprofloxacin (Fig 12D). qRT-PCR showed that the transcription levels of *epel1*, *epel2*.*1*, and *epel2*.*2* were clearly lower in FY26Δ*recA* cultured with ciprofloxacin than in of WT FY26 incubated with ciprofloxacin (*P*≤0.01) (S13B Fig). It was noteworthy that antibiotics stimulated the production of ExPEC EVs by affecting DNA damage and the dynamic balance of cell envelope synthesis.

Moreover, to identify whether incubation of *epel* mutants with antibiotic caused an increase in cytoplasm-carrying EVs production, the triple-deleted mutant FY26Δepel*1*/*2*.*1*/*2*.*2* were cultured in medium with sublethal level of ciprofloxacin. After 12h incubation, EVs were isolated, and the number of vesicles production from FY26Δepel*1*/*2*.*1*/*2*.*2* was determined. NTA analysis showed that the EVs production of FY26Δepel*1*/*2*.*1*/*2*.*2* incubated with ciprofloxacin was close to that of FY26Δepel*1*/*2*.*1*/*2*.*2* cultured under routine condition (S13G and S12B Figs). Moreover, immunofluorescent images showed that the proportion of cytoplasm-carrying EVs of FY26Δepel*1*/*2*.*1*/*2*.*2* incubated with ciprofloxacin was close to that of FY26Δ*epel1*/*2*.*1*/*2*.*2* cultured under routine condition (S13H Fig). The DNA damage induced by incubation with ciprofloxacin activated RecA/LexA-dependent SOS response, increasing the production of ExPEC cytoplasm-carrying EVs. The formation of cytoplasm-carrying EVs when ExPEC was exposed under ciprofloxacin stress was dependent upon the expression of endolysin, induced by the SOS response.

To determine whether the antibiotics affected the DNA content of the ExPEC EVs, the total DNA in PK and DNase-treated EVs from routinely cultured FY26 or FY26 cultured with a sublethal concentration of antibiotics was used to measure the DNA content in the EV cargoes. When FY26 was cultured for 12h, about 7.1×10^11^ EV, contained 110 ng of DNA. At an equivalent concentration of vesicles (7.1×10^11^), more DNA was detected in the EVs from FY26 cultured with a sublethal dose of specific antibiotics (ampicillin, 13.2-fold; ceftazidime, 12.9-fold; imipenem, 12.4-fold; ciprofloxacin, 17.3-fold; chloramphenicol, 12.7-fold; colistin, 11.4-fold), compared with the EV production in FY26 cultured without antibiotics (*P* ≤ 0.01) (S14A Fig). However, there was no obvious difference in the DNA content of an equivalent number of the EVs from FY26 cultured with a sublethal concentration of sulfamethoxazole (S14A Fig). Moreover, we used polyacrylamide gel electrophoresis to analyze the DNA cargoes in EVs from routinely cultured FY26 or FY26 cultured with antibiotics. Similarly, more DNA was detected in the EVs from FY26 cultured with a sublethal dose of antibiotics than in EVs form routinely cultured FY26 (S14B–S14C Fig).

We also used a multi-drug resistant extended spectrum beta-lactamase (ESBL)-producing ExPEC ST95 isolate, ST95-32 (BioSample accession: SAMN12757101), to evaluate whether drug-resistant strains incubated with the antibiotics to which they were resistant promoted the production of EVs. Strain ST95-32 was resistant to more than a dozen antibiotics, including ampicillin, ceftazidime, levofloxacin, and chloramphenicol. The number of EVs produced by strain ST95-32 was about 1.9×10^10^ when it was cultured in 1.0 L of LB medium without antibiotics for 12 h. However, when it was incubated with lethal doses of antibiotics, vesicle production was enhanced (ampicillin, 17.9-fold; ceftazidime, 15.3-fold; levofloxacin, 4.1-fold; chloramphenicol, 13.2-fold) relative to the vesicle production by the routinely cultured strain (*P* ≤ 0.01) (S14D and S12C Figs). Obviously, the treatment of resistant ExPEC with antibiotics continually enhanced the production of EVs.

### Cytotoxicity of ExPEC EVs

**Fig 13:**
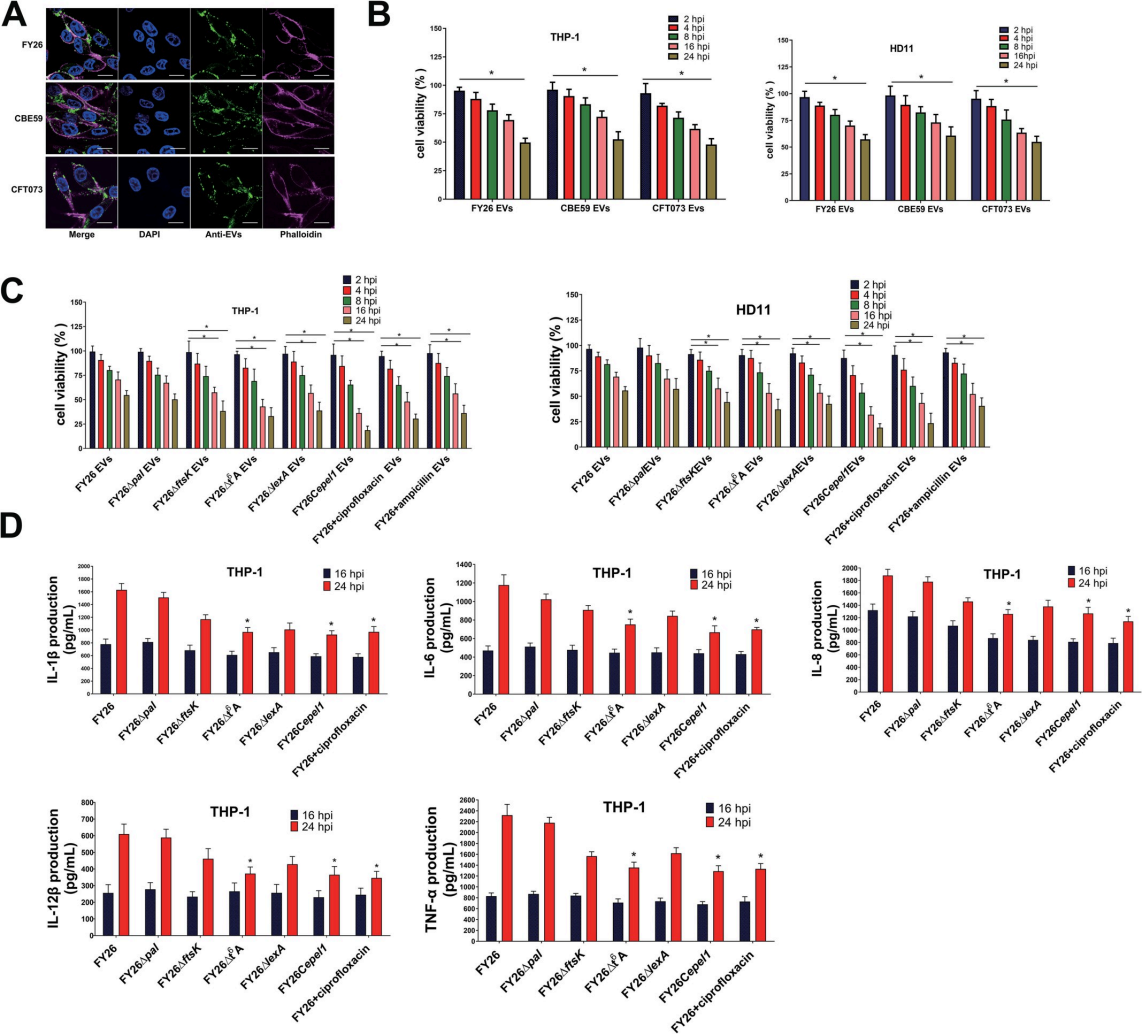
Cytotoxicity of ExPEC EVs. **(A)** Confocal microscopic visualization of the internalization of ExPEC EVs into HD11 macrophages. Infected HD11 cells were incubated with anti-ExPEC antibody, and stained with FITC-conjugated goat anti-mouse IgG antibody (green), phalloidin (red), and DAPI (blue). Images show a representative of at least three independent experiments. Scale bar: 10 μm. **(B)** To investigate the cytotoxic effects of ExPEC EVs on THP-1 and HD11 macrophages, THP-1 or HD11 cells were incubated with EVs (50 μg/mL) for different periods (2, 4, 8, 16, or 24 h). Cell viability was measured with a CCK-8 kit. The results are presented as means ± SEM of at least three independent experiments. One-way ANOVA was used to evaluate statistical significance (*P < 0.01). **(C)** To investigate the cytotoxic effects of ExPEC EVs on THP-1 and HD11 cells, EVs were isolated from mutant strains FY26Δ*ftsK*, FY26Δt6A, and FY26Δ*lexA*, the endolysin-overexpressing strain FY26Cepel1, and antibiotic-treated strain FY26. **(D)** The cytokines (IL-1β, IL-6, IL-8, IL-12β, and TNF-α) from the cell-free supernatant of THP-1 macrophages was measured using the commercial cytokine ELISA kits. The results are presented as means ± SEM of at least three independent experiments. One-way ANOVA was used to evaluate statistical significance (*P < 0.01).

It has been documented that EHEC O157 OMVs carry virulence factors and deliver toxins to the eukaryotic host cells, which can result in cell death [28]. Immunofluorescent labeling showed that ExPEC EVs were internalized by macrophages (Fig 13A). To determine the effect of their interaction with macrophages (HD11 and THP-1), the cytotoxic effect on macrophages of incubation with ExPEC EVs was determined with a CCK-8 assay at several time points. The cytotoxic effect of WT FY26 EVs on THP-1 macrophages was observed at 8 h post-infection (hpi), and macrophage viability was markedly reduced at 24hpi (Fig 13B). A similar cytotoxic phenomenon was also observed when THP-1 macrophages were treated with CBE59 or CFT073 EVs (Fig 13B). Similarly, a cytotoxic effect on HD11 macrophages incubated with ExPEC EVs was also observed (Fig 13B). The EVs produced by the mutant strains FY26Δ*ftsK*, FY26Δt6A, and FY26Δ*lexA*, the endolysin-overexpressing strain FY26Cepel1, and antibiotic-treated FY26 were more seriously cytotoxic to THP-1 and HD11 macrophages at 16hpi and 24hpi than the EVs obtained from WT FY26 cultured under routine conditions (Fig 13C). The cytotoxicity of the EVs of *pal*-deficient strain FY26Δ*pal* was similar to that of the WT FY26 EVs. Therefore, the content ratio of cytoplasm-carrying vesicles among the ExPEC EVs influenced their cytotoxicity. ExPEC EVs with a higher proportion of cytoplasm-carrying vesicles was more toxic to macrophages than EVs with a smaller proportion of cytoplasm-carrying vesicles. Remarkably, a proteomic analysis revealed that virulence-related proteins were less abundant or completely undetected in the ExPEC EVs. However, cytosolic/periplasmic proteins were significantly enriched in the ExPEC EVs. Because ExPEC strain FY26 EVs lacked toxins (HlyA, CdtV, Sat, and Pic), which were also not abundant in CFT073 EVs, we speculated that some specific cytoplasmic and periplasmic proteins enriched in cytoplasm-carrying vesicles might be cytotoxic.

Furthermore, we identified the effect of ExPEC EVs on the production of pro-inflammatory cytokines in THP-1 macrophages. PMA-differentiated THP-1 cells were incubated with EVs (50 μg/mL) for 16h and 48 h, respectively. To determine the effect of ExPEC EVs on the release of pro-inflammatory cytokines, ELISA assays were performed to measure the production of pro-inflammatory cytokines, including IL-1β, IL-6, IL-8, IL-12β, and TNF-α, in THP-1 macrophages. As shown in Fig 13D, we observed a time-dependent release of pro-inflammatory cytokines in THP-1 macrophages. THP-1 cells were incubated with EVs from FY26, several mutants, and endolysin complementary strain, leading to the release of pro-inflammatory cytokines. Similar to other bacterial OMVs, ExPEC EVs acted as powerful pro-inflammatory stimulators of macrophages. However, the release levels of pro-inflammatory cytokines (IL-1β, IL-6, IL-8, IL-12β, and TNF-α) for THP-1 macrophages incubated by EVs produced by the mutant FY26Δt6A and complementary strain FY26Cepel1, and isolated from WT FY26 treated with Ciprofloxacin were obviously lower than that of WT FY26 at 24 h (*P* < 0.01). Compared to EVs of WT FY26, EVs with higher proportion of cytoplasm-carrying vesicles reduced the production of pro-inflammatory cytokines in THP-1 cells. One obvious reason for this phenomenon was that ExPEC EVs with higher proportion of cytoplasm-carrying vesicles led to an obvious reduction in viable macrophages by cytotoxicity assays.

### Increased proportion of cytoplasmic vesicles in ExPEC EVs causes more-severe mitochondrial dysfunction and higher apoptosis rate in macrophages

**Fig 14:**
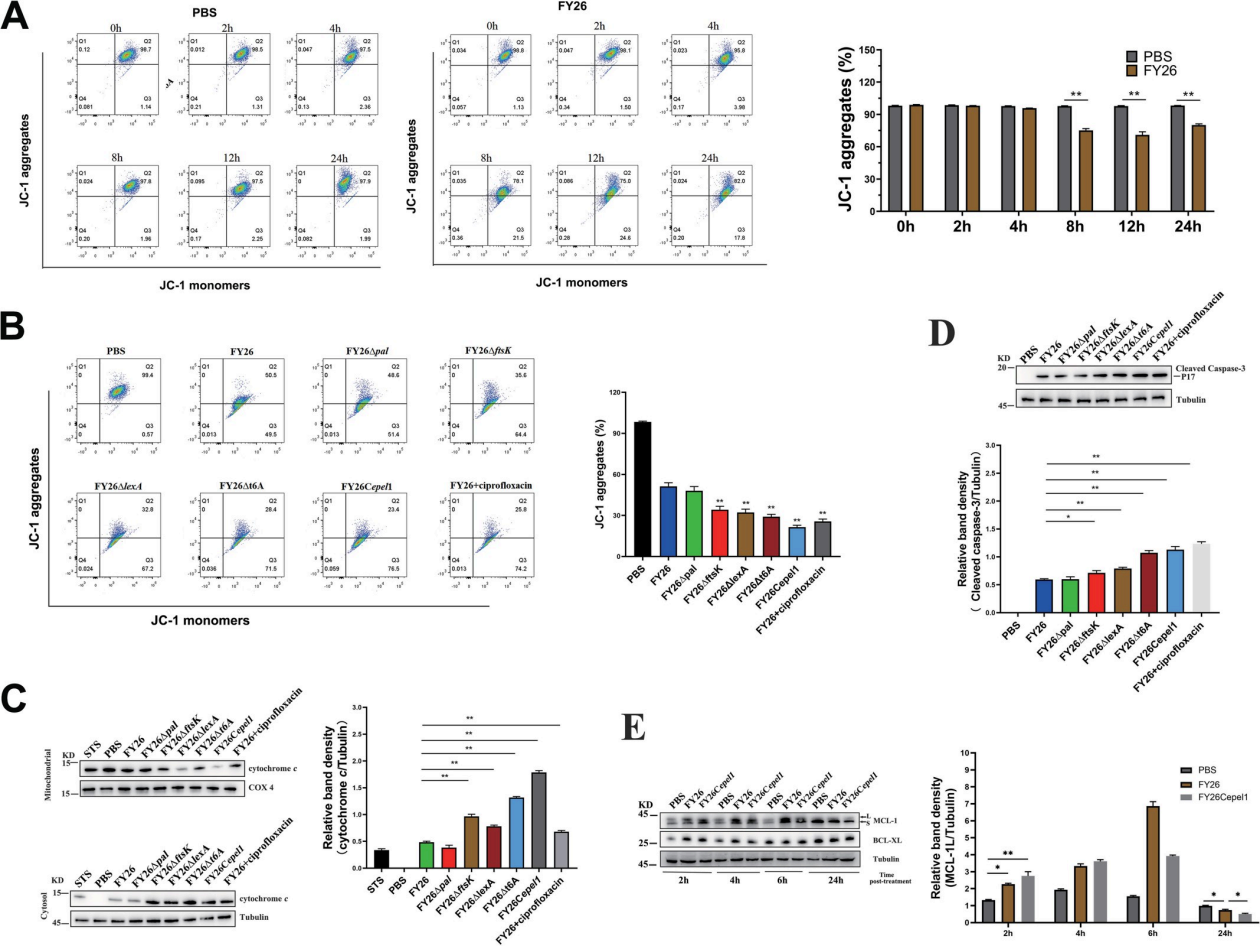
Increased proportion of cytoplasmic vesicles in ExPEC EVs caused more-severe mitochondrial dysfunction and apoptosis in macrophages. **(A)** Mitochondrial membrane potential (ΔΨm) was determined with JC-1 dye and flow cytometry. Bone-marrow-derived macrophages (BMDMs) were treated with FY26 EVs (50 μg/mL) or PBS for 0, 2, 4, 8, 12 or 24 h and labeled with JC-1. The percentage of normal cells (JC-1 aggregates) in BMDMs is indicated on the right. **(B)** Mitochondrial membrane potential (ΔΨm) was determined with JC-1 dye and flow cytometry. Bone-marrow-derived macrophages (BMDMs) were treated with purified ExPEC EVs (50 μg/mL) or PBS for 12 h and labeled with JC-1. The percentage of normal cells (JC-1 aggregates) in BMDMs is indicated on the right. **(C)** Cytosolic and mitochondrial fractions of BMDMs were analyzed for cytochrome *c* with western blotting. BMDMs were treated with purified ExPEC EVs (50 μg/mL), staurosporine (STS), or PBS for 24 h, anti-tubulin and anti-COX4 were used as the loading control. Molecular weight markers are shown on the left. **(D)** Levels of caspase 3 (17 kDa) in the BMDMs were determined with western blotting. BMDMs were treated with purified ExPEC EVs (50 μg/mL) or PBS for 24 h; anti-tubulin was used as the loading control. **(E)** Levels of MCL-1 and BCL-XL in the BMDMs were determined with western blotting. BMDMs were treated with purified ExPEC EVs (50 μg/mL) or PBS for 2, 4, 6, or 24h; anti-tubulin was used as the loading control.

Previous studies have shown that macrophages exposed to EVs from Gram-negative bacteria display mitochondrial dysfunction, activated intrinsic apoptosis, and inflammation [74]. To investigate whether the enhanced cytotoxicity of ExPEC EVs is associated with mitochondrial dysfunction and apoptosis, the mitochondrial membrane potential was detected with JC-1 dye at several time points. The exposure of bone-marrow-derived macrophages (BMDMs) to EVs from WT FY26 reduced their mitochondrial membrane potential at 8 hpi, and the mitochondrial membrane potential was significantly reduced at 12 hpi (*P* < 0.01; Fig 14A). Similarly, the mitochondrial membrane potential of BMDMs decreased after exposure to EVs produced by the mutant strains FY26Δ*ftsK*, FY26Δt6A, and FY26Δ*lexA*, the endolysin-overexpressing strain FY26Cepel1, and the antibiotic-treated FY26 compared with that of WT FY26 at 12 hpi (*P* < 0.01; Fig 14B). We then examined whether exposure to ExPEC EVs led to cytochrome *c* release from mitochondria and the activation of intrinsic apoptosis. Cytochrome *c* was released into the macrophage cytosol from the mitochondria at 24 hpi when BMDMs were treated with FY26 EVs (Fig 14C), and the exposure of BMDMs to EVs produced by the mutant strains FY26Δ*ftsK*, FY26Δt6A, and FY26Δ*lexA*, the endolysin-overexpressing strain FY26Cepel1, and antibiotic-treated FY26 led to an increase in cytosolic cytochrome *c* at 24 hpi compared with that in BMDMs treated with WT FY26 EVs (*P* < 0.01; Fig 14C). Cytochrome *c* helps to activate the cell death killer proteases, the caspases. Caspase 3 plays a crucial role in apoptosis. Cleaved caspase 3 was only detectable in BMDM cells after their treatment with ExPEC EVs, but not in the control phosphate-buffered saline (PBS)-treated cells (Fig 14D). BMDMs treated with EVs produced by mutant strains FY26Δ*ftsK*, FY26Δt6A, and FY26Δ*lexA*, the endolysin-overexpressing strain FY26Cepel1, or antibiotic-treated FY26 displayed significantly increased levels of cleaved caspase 3 at 24 hpi compared with those treated in BMDMs with EVs produced by WT FY26 (all *P* < 0.01; Fig 14D). It has been documented that the mitochondrial dysfunction and intrinsic apoptosis caused by the treatment of macrophages with OMVs is dependent on B-cell lymphoma 2 (BCL-2) family members [74]. To explore the role of BCL-2 family members in EV-induced intrinsic apoptosis, the levels of anti-apoptotic MCL-1 and BCL-XL in EV-treated BMDMs were determined with western blotting. Compared with PBS-treated cells, the level of MCL-1 but not BCL-XL in FY26 EV-treated BMDMs were initially increased (*P* < 0.05), and the level of the long isoform of MCL-1 were decreased at 24 hpi (*P* < 0.05; Fig 14E). The reduction in the long isoform of MCL-1 was most pronounced in the FY26C*epel1*-EV-treated BMDMs (*P* < 0.01), suggesting that the mitochondrial dysfunction and intrinsic apoptosis induced by EVs depleted pro-survival MCL-1L. Taken together, these data indicate that when macrophages are exposed to a higher proportion of cytoplasm-carrying vesicles, more-severe mitochondrial disruption and a higher level of intrinsic apoptosis were induced because the cytotoxicity of ExPEC cytoplasm-carrying vesicles was increased.

## Discussion

**Fig 15:**
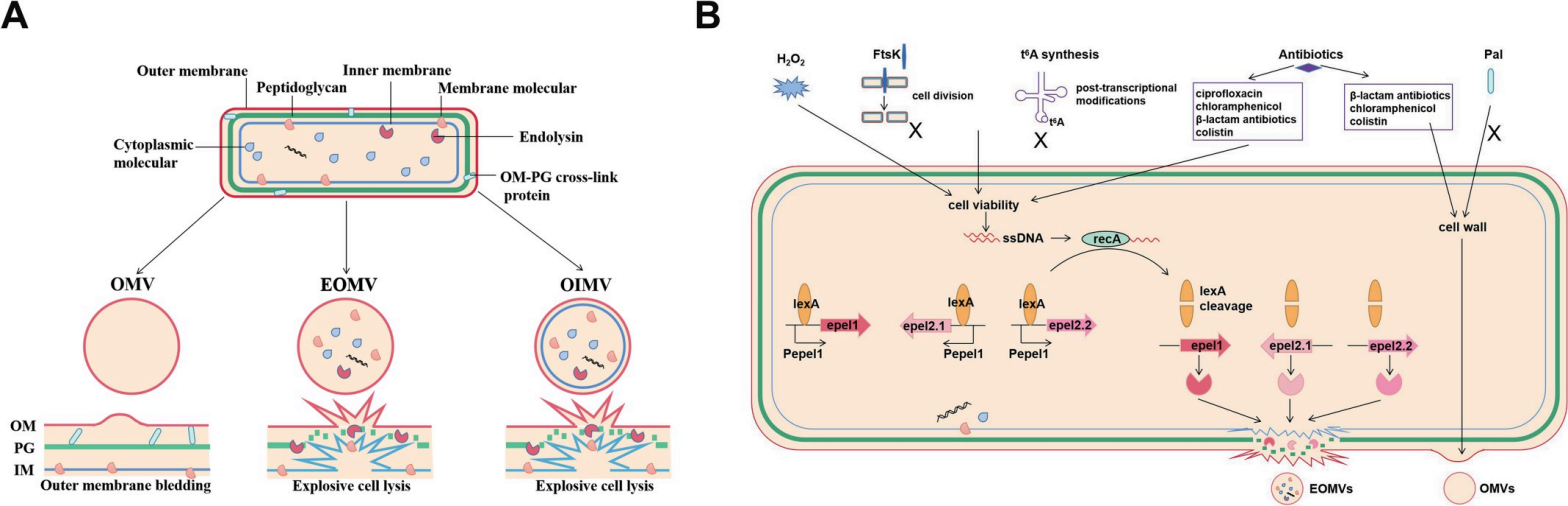
Illustration of the generation mechanisms of EVs in ExPEC. **(A)** The biogenesis mechanisms for different types ExPEC EVs. ExPEC EVs can be considered a mixture of classical outer-membrane vesicles (OMVs) and cytoplasm-carrying vesicles: explosive outer-membrane vesicles (EOMVs) and outer–inner-membrane vesicles (OIMVs). Therefore, typical OMVs, formed through the blebbing of the bacterial outer membrane from the envelope, have a single-layered membrane, which originated from the unbalanced biosynthesis of the cell envelope. A defect in the crosslinking between peptidoglycan and the outer membrane leads to the production of OMVs. The “explosive cell lysis” model is a possible mechanism for the formation of ExPEC EVs, in which the bacterial peptidoglycan is degraded by phage-derived endolysins to induce explosive cell lysis, and the broken membrane fragments gather and self-assemble to form EOMVs (single-layered membrane) or OIMVs (bilayer membrane). **(B)** Model of the generation mechanism of ExPEC cytoplasm-carrying vesicles. The unbalanced cell division caused by the deletion of the *ftsK* gene and t^6^A synthesis defects or by the toxicity caused by exposure to H_2_O_2_ reduces ExPEC viability, thus increasing the production of cytoplasm-carrying vesicles through the RecA/LexA-dependent SOS response. Two regulatory proteins (RecA and LexA) control the expression of the SOS response genes in *E*. *coli*. This study demonstrated that the repressor LexA directly suppresses the expression of endolysins (Epel1, Epel2.1, and Epel2.2) by binding to the SOS boxes in the endolysin promoter regions. In response to DNA damage, the binding of RecA to single-stranded RNA activates RecA to stimulate the autocatalytic cleavage of the LexA repressor. The expression of prophage-associated endolysins is then activated, which triggers cell lysis and increases the production of ExPEC cytoplasm-carrying vesicles. Antibiotic treatment also reduces bacterial viability, thus increasing the production of ExPEC cytoplasm-carrying vesicles through the RecA/LexA-dependent SOS response. In contrast, the deletion of *pal* only causes a peptidoglycan crosslinking defect, which promotes the formation of classic OMVs.

Even after many years of research, the molecular mechanisms underlying the presence of cytosolic contents and nucleic acids (DNA and RNA, etc.) in bacterial OMVs remained largely unexplained [20,34,38,75]. There are very few studies on how nucleic acids and cytoplasmic proteins pass through the bacterial inner membrane and are packaged into OMVs [76] (Fig 15A). It is possible that cytoplasm-carrying vesicles are not bona fide OMVs. Typical OMVs may be unable to pack cytoplasmic contents directly [20]. Several studies have shown that a defect in the interconnection between the outer membrane and the peptidoglycan layer increases the production of OMVs [46,77]. A defect in the Tol-Pal system also caused a leaky phenotype, in which outer-membrane and periplasmic proteins are released into the extracellular medium [78]. In the present study, we have shown that the deletion of *pal* damaged the integrity of the bacterial membrane, leading to the increased production of ExPEC EVs, notably OMVs. Previous studies have shown that the deletion of components of the Tol-Pal complex increases both OMVs and OIMVs production, and the Tol-Pal complex is considered to be the master regulator of OMVs [48,77,79,80]. However, the proportion of cytoplasm-carrying EVs (including OIMVs) in the total vesicles of the *pal* mutant decreased relative that in the vesicles of WT ExPEC. The expression levels of cytoplasmic and ribosomal proteins were not significantly increased in the EV-free extracellular medium of the *pal* mutant. No obvious cell lysis was detected after the deletion of the *pal* gene.

The model of the formation of OIMVs explains the important mechanism by which cytoplasmic contents are packaged into vesicles [20,81]. The peptidoglycan layer between the bacterial double membranes is weakened and the inner membrane is pushed out and enters the periplasm. The cytoplasmic contents consequently enter into the vesicles (Fig 15A). The OIMVs are then pinched off from the bacterial surface and are defined by a surrounding outer membrane. Therefore, OIMVs contain both the outer and inner membranes, and the cytoplasmic contents are specifically packed into OIMVs [81,82]. The proportion of OIMVs ranges from 0.1% of the total EVs in *Shewanella vesiculosa* M7 to about 49% in *Pseudoalteromonas marina* [81,83]. OIMVs with a double-bilayer structure have also been identified in ExPEC EVs with TEM.

A newer view, the “explosive cell lysis” model, offers another possible mechanism for the formation of ExPEC EVs [24]. Turnbull et al. demonstrated for the first time that the production of EOMVs in *P*. *aeruginosa* is the result of explosive cell lysis, which induces the expression of a prophage endolysin [20,24]. Once the peptidoglycan cell wall is degraded by phage-derived endolysins, the bacterial cells aggregate and explode, and the broken membrane fragments gather and self-assemble into EOMVs, engulfing the cytoplasmic components within the vesicles (Fig 15A). Our proteomic analysis showed that cytoplasmic proteins are packed into ExPEC EVs. The proportion of EOMVs produced in ExPEC strains was calculated with a simple GFP expression method that identified cytoplasm-carrying vesicles. Our findings showed that three ExPEC endolysins (Epel1, Epel2.1, and Epel2.2) caused serious cell lysis. Cytoplasmic proteins and ribosomal proteins were identified in the EV-free culture medium when the ExPEC strains were routinely cultured. Endolysin-triggered cell death in the ExPEC strains promoted the production of ExPEC cytoplasm-carrying EVs (OIMVs and EOMVs) (Fig 15B). The cytoplasmic-carrying vesicles observed with immunofluorescence were considered to be EOMVs. ExPEC EVs can be a mixture of OMVs, EOMVs, and OIMVs, and the proportion of cytoplasm-carrying EVs produced by ExPEC was consistent with the abundance of cytoplasmic proteins.

In *P*.*aeruginosa*, the overexpression of endolysin causes explosive cell lysis, increasing EV production through a vesicularization process, with the self-annealing and curling of outer-membrane fragments. The shattered outer-membrane fragments are likely to capture the cellular components released into the extracellular milieu [24,84]. In *S*.*vesiculosa* M7, most re-annealed EVs generated by explosive cell lysis display the same staining profile as the outer membrane of *S*.*vesiculosa* [85]. Moreover, some IOMVs have only a partial inner membrane. The inner membrane of OIMVs can be partly separated from the peptidoglycan layer, whereas the outer membrane is still tightly attached to the peptidoglycan layer, suggesting that EOMVs are derived from OIMVs [86]. These findings seem to demonstrate that the formation of EOMVs, which are naturally secreted from Gram-negative bacteria, is attributable to the recircularization of outer membrane. Our results also showed that the level of cytoplasmic proteins increased with the increasing production of cytoplasm-carrying membrane vesicles, whereas the level of outer-membrane proteins did not change appreciably. This suggests that the cytoplasm-carrying membrane vesicles originate from the outer membrane. Moreover, even if the inner-membrane vesicles are formed by the recircularization of the bacterial inner membrane, after the peptidoglycan layer is degraded by endolysins, these vesicles should be less stable and may last for a shorter period of time. Because the inner-membrane vesicles contain neither the outer-membrane-related lipopolysaccharide layer nor the inner-membrane peptidoglycan layer, the inner-membrane vesicles are more easily degraded than OMVs or EOMVs.

The SOS response is a general bacterial stress response that is induced by bacterial DNA damage, triggered by various stressors, such as antibiotics [87]. It was based that the SOS-dependent expression of endolysins not only leads to cell death but also significantly enhances EV production. Earlier research identified explosive cell lysis as a novel mechanism of vesicle formation in *P*. *aeruginosa* [24,88]. In this study, we have shown that the RecA/LexA-dependent SOS response induces the expression of endolysins, promoting the formation of ExPEC cytoplasm-carrying EVs. Moreover, under the SOS response, many LexA-binding sites have been identified in the promoter regions of genes that are not involved in DNA repair or synthesis [59,63,89]. Several putative endolysin genes were predicted in strains FY26, CBE59, and CFT073, but LexA-binding sites were only present in the promoter regions of our named endolysin genes. Our EMSA results showed that LexA bound directly to the promoter DNA of *epel1*, *epel2*.*1*, and *epel2*.*2* to block the expression of ExPEC endolysins. Prophage endolysins are expressed during the induction of lysogenic phages. It has been reported that the induction of lysogenic phages that carry endolysin genes increases the production of bacterial EVs [90]. Although ExPEC strains generally carry prophage genomes, only some strains can induce lysogenic phages. The phage genomes are recognized as the exogenous DNA by the bacterial defense system, and this is followed by DNA recombination and DNA loss in the prophage gene region, rendering the bacterium unable to induce the production of phages. Importantly, the expression of ExPEC endolysins was directly controlled by the LexA/RecA system. Although ExPEC prophages cannot be induced, the expression of endolysins can still be activated.

The H_2_O_2_-induced SOS response activated endolysin expression, triggering cell lysis and the production of ExPEC cytoplasm-carrying EVs. The H_2_O_2_-induced SOS response activated endolysin expression to trigger cell lysis, as a result of the production of ExPEC cytoplasm-carrying EVs. Orench-Rivera et al. reported that treatment of H_2_O_2_ did not significantly alter OMV production in enterotoxigenic *E*. *coli* (ETEC) [91]. Our study showed that the oxidative treatment led to an increase in production of ExPEC EVs. This discrepancy was likely due to the difference for bacterial culture method. After ETEC culture for 16 hours, Orench-Rivera et al. collected the bacteria by centrifugation and resuspended bacteria in fresh LB, and then the cultures were incubated for another 3 h with H_2_O_2_ (32mM). H_2_O_2_ with high concentration significantly reduced bacteria growth, and even caused bacteria death. In addition, the culture time for another 3 h might be too shorter to produce more EVs. Macdonald et al. reported that OMVs production increases after treatment with H_2_O_2_ in *P*. *aeruginosa* [92]. OMVs production in *P*. *aeruginosa* was not dependent on activation of quinolone signal (PQS) and the periplasmic protease MucD, which are critical factors in production of bona fide OMVs [92]. *P*. *aeruginosa* B-band lipopolysaccharide, but not A-band, is involved in H_2_O_2_-induced OMVs production. This study does not further classify H_2_O_2_-induced membrane vesicles [92]. It is possible that the so-called OMVs in the Macdonald & Kuehn research are not only considered as the bona fide OMVs. The classic OMVs in early research are usually considered equivalent to bacterial EVs [25]. The membrane vesicles induced by H_2_O_2_ in *P*. *aeruginosa* might harbor cytoplasm-carrying EVs (EOMVs and OIMVs). Similarly, reduction in bacterial viability with the sublethal level of H_2_O_2_ (1.0 mmol/L) in our experiments could stimulate the production of ExPEC cytoplasm-carrying EVs.

Evidence has accumulated that the production of EVs that are usually recognized as OMVs, depends on the bacterial growth conditions, such as the medium composition, oxygen availability, iron, and presence of antibiotics [93,94]. However, most reports do not distinguish whether the growth conditions affect the generation of OMVs or cytoplasm-carrying vesicles (EOMVs and OIMVs). In this study, several new findings showed that a reduction in bacterial viability stimulated the production of ExPEC EVs, especially EOMVs (Fig 15B). The imbalance in cell division caused by exposure to H_2_O_2_, the deletion of the *ftsK* gene, and defects in t^6^A synthesis activated the RecA/LexA-dependent SOS response, inducing the expression of endolysins, and thus increasing the proportion of cytoplasm-carrying EVs in the total ExPEC EVs. Our findings show that FtsK and the t6A synthesis proteins (YrdC, YgjD, YeaZ, and YjeE) are novel molecular targets for the promotion of EV formation. Some researchers have proposed that a t^6^A synthesis-related protein can be a target in the development of small molecule drugs [95]. However, we consider that even if it were effective, the disturbance of t^6^A synthesis would increase the release of bacterial EVs. Moreover, many antibiotics reduce bacterial viability by causing DNA damage and disturbing the dynamic balance of cell envelope synthesis, which could increase the production of ExPEC cytoplasm-carrying EVs through the SOS response. The DNA damage induced by incubation with ciprofloxacin activated RecA/LexA-dependent SOS response, increasing the production of ExPEC cytoplasm-carrying EVs. Our findings suggest that chloramphenicol also induces the SOS response, but to a lesser extent. The *β*-lactam antibiotics and colistin also activated the expression of endolysins through the RecA/LexA-dependent SOS response pathway, thus increasing the production of cytoplasm-carrying EVs. They may also promote the production of OMVs by targeting the cell wall at the same time. ExPEC EVs had a cytotoxic effect on THP-1 and HD11 macrophages. Ribosomal proteins have functions beyond the ribosome and act as regulatory proteins in the management of disease [34,96]. However, no classical virulence factor was enriched in ExPEC EVs. It remains to be explored how the key effector proteins or exogenous sRNAs carried by ExPEC EVs manipulate the expression of cellular immunity-related signaling pathways. In future research, it would be important to unravel to reveal the interaction receptor proteins of key effector proteins or host cell mRNAs that are directly targeted and regulated by exogenous sRNAs.

Bacterial EVs are observed in clinical samples of blood plasma and cerebrospinal fluid from patients with severe bacterial extra-intestinal infections [97,98]. ExPECs may release EVs into particular body fluids and urine during their infection of the bloodstream or urinary tract. We speculate that the biological heterogeneity and molecular cargoes of EVs produced *in vivo* by infectious ExPECs might differ from those of EVs derived from routinely cultured bacteria. A greater number of cytoplasm-carrying EVs is generated when ExPECs confront antibacterial pressures in the body. It has been reported that exposure to sublethal concentrations of antimicrobial agents *in vitro* accelerates the evolution of bacterial resistance, which leads, in turn, to the formation of bacterial multidrug resistance [99]. Bacteria within the host also develop multidrug resistance. *Salmonella* Typhimurium can spontaneously transmit resistance genes to bacteria colonizing the host intestine [100]. Sublethal levels of antibiotics facilitate bacterial adhesion to and colonization of host epithelial cells [101]. It seems that our study could explain the roles of EVs in the formation and spread of drug resistance during bacterial extra-intestinal infections. Our findings show that the treatment of multidrug-resistant ExPEC with antibiotics enhances the production of EVs (Fig 15B). The DNA content of ExPEC EVs was consistent with the proportion of cytoplasm-carrying EVs. EOMVs and OIMVs can carry large plasmids, accelerating the horizontal transfer of drug resistance and virulence factors. For these reasons, the antibiotic treatment of multidrug-resistant bacterial infections could be potentially harmful to the host. The misuse or unreasonable use of antibiotics could enhance the cytotoxicity of bacterial EVs to host cells.

Although we could accurately calculate the proportion of cytoplasmic vesicles with GFP-based detection, we also confirmed the presence of OIMVs in ExPEC (CBE59, FY26 and CFT073) EVs with TEM. However, given the limitations of electron microscope observation, we could not accurately determine the total proportion of OIMVs in the ExPEC EVs or the proportion of EOMVs. We used an EMC-200KeV Cryo-electron Microscope to observe the EVs, but Cryo-TEM could not individually classify the vesicles as double-membrane or single-layered vesicles one by one. In future research, it will be important to clarify the proportion of OIMVs in total ExPEC EVs, especially in endolysin-overexpressing strains, with Glacios Cryo-electron Microscope. Moreover, when the cytoplasm-carrying vesicles were detected with fluorescent microscopy analysis, the apparently large particles were observed. This phenomenon suggested that a small part of the labeled EVs seemed to aggregate to form larger particles. Due to a significantly smaller number of the larger particles in image observation, the aggregation of EVs could not affect the proportion estimation of cytoplasm-carrying vesicles in the total ExPEC EVs.

In conclusion, this work represents the first high-throughput proteomic study to identify the contents of cytoplasmic proteins in ExPEC EVs. Our findings show that ExPEC strains produce three types of EVs, including OMVs and cytoplasm-carrying EVs (OIMVs and EOMVs). Endolysin-triggered cell lysis is an important way that *E*. *coli* produces EVs. The total content of cytoplasm-carrying EVs in ExPEC EVs changes dynamically and is mainly determined by the stress response. Our research provides novel insights into the formation mechanisms of ExPEC cytoplasm-carrying EVs, including a change in cell homeostasis and DNA damage, which weaken bacterial viability. A comprehensive understanding of the formation mechanism of ExPEC EVs is extremely important for the prevention and treatment of ExPEC infections. Overall, our study provides further insights to facilitate the management of multi-drug resistant bacteria. Understanding of formation mechanisms of ExPEC EVs is critical to defining bacterial extra-intestinal infections for public health purposes.

## Materials and methods

### Ethics statement

All animal research protocols were performed according to the Experimental Animal Management Measures of Jiangsu Province, and were approved by the Ethical Committee for Animal Experiments of Nanjing Agricultural University (SYXK[SU]2011–0036), Nanjing, China.

### Strains and plasmids

The plasmids, bacterial strains, and PCR primers used in this study are listed in S4 and S5 Tables. To construct the plasmid containing the sulfonamide resistance gene, *sul1*, the gene was amplified and digested with *Nhe*Ι. The fragment was then ligated into plasmid pSTV28 (TaKaRa, Shiga, Japan) using the ClonExpress II One Step Cloning Kit (Vazyme, Nanjing, China) to generate the plasmid pSTV28–*sul1*. To construct the green fluorescent protein (GFP)-expressing plasmid, a fragment containing the complete GFP-encoding gene, the *ptac* promoter, and the *rrnB* terminator sequence was generated by gene synthesis (Sangon, Shanghai, China). The GFP fragment was digested with *Bsu*36Ι (*Eco*R81Ι) and cloned into the plasmid pSTV28–*sul1* to generate the final plasmid pSTV28–GFP–*sul1*. The appropriate strains were transformed with the constructed plasmid by electroporation. To construct a plasmid expressing the LexA fusion protein, the *lexA* gene was cloned into expression plasmid pET-28a. *Escherichia coli* BL21(DE3) cells (Vazyme) were transformed with the recombinant plasmid. The LexA protein was purified with Ni-chelating chromatography (GE Healthcare, Pittsburgh, PA, USA).

The mutants were constructed in WT strain FY26 using the λ-Red homologous recombination system, as previously described [102]. Mutants containing double or triple deletions of *epel1*, *epel2*.*1*, and *epel2*.*2* were constructed in FY26 with the scarless deletion method [54]. The genes containing the predicted promoter fragments were amplified from FY26 genomic DNA, and the PCR products were cloned into the plasmid pSTV28–*sul1* to construct the complementary plasmids. The complemented strains were constructed by transforming the mutants with the complementary plasmids by electroporation. The *epel1*, *epel2*.*1*, and *epel2*.*2* complementary plasmids were introduced individually into the triple-deleted mutant FY26Δ*epel1/2*.*1*/*2*.*2*.

### Cell culture

THP-1 cells (a human monocytic cell line) were cultured in RPMI 1640 medium (Gibco) supplemented with 10% fetal bovine serum (FBS; Gibco) at 37°C in a humidified 5% CO_2_ atmosphere. Phorbol 12-myristate 13-acetate (PMA; 50 ng/mL; Sigma-Aldrich) was used to induce the macrophage-like differentiation of the THP-1 cells, and the cells were then cultured for 24 h [103]. HD11 cells (a chicken macrophage cell line) were cultured in Dulbecco’s modified Eagle’s medium (DMEM; Gibco) supplement with 10% FBS, in a humidified 5% CO_2_ atmosphere at 37°C.

Murine BMDMs were obtained from the femoral and tibial bones of 6–8-week-old mice, and cultured in RPMI 1640 medium (Gibco) supplemented with 15% FBS, (Gibco), 100 U/mL penicillin–streptomycin, 25 mM HEPES, and 50 ng/mL macrophage colony-stimulating factor (MCSF) for 6 days (37°C, 5% CO_2_).

### Growth curve

In the growth experiments, strains FY26, CFT073, and CBE59 were cultured in Luria–Bertani (LB) medium overnight at 37°C. The cultured bacteria were centrifuged to remove the culture supernatant. After the bacterial cells were washed twice with phosphate-buffered saline (PBS), their concentrations were adjusted to an OD_600_ of 1.0, and 500 μL of the adjusted bacteria were used to inoculate 50 mL of LB medium. Bacterial growth was monitored continuously by measuring the OD_600_ of the culture at different times with a spectrophotometer (Philes, Nanjing, China). The growth experiment was performed at least three times with three replicates of each sample.

### Minimum inhibitory concentration (MIC) assays of antibiotics and H_2_O_2_

To determine the sublethal concentrations of various antibiotics and hydrogen peroxide (H_2_O_2_) for FY26 and FY26Δ*recA*, an MIC assay was performed as previously described [104]. Briefly, bacteria were diluted to 1 × 10^8^ colony-forming units (CFU) per mL, and 30% H_2_O_2_ or various antibiotics were serially diluted 10-fold in LB medium. Aliquots (100 μL) of the H_2_O_2_ and antibiotic dilutions were added separately to the wells of a 96-well plate containing 100 μL of bacteria (final volume of 200 μL). The plates were incubated in 37°C for 24 h, and the OD_600_ of each well was measured with a Spark Cyto microplate reader (Tecan, Mannedorf, Switzerland). The MICs of the antibiotics ampicillin, ceftazidime, levofloxacin, and chloramphenicol for ExPEC strain ST95-32 were determined as described above. The MIC assay was performed at least three times on each sample.

### Isolation and purification of extracellular vesicles (EVs)

Bacterial EVs were isolated and purified as previously described, with slight modifications [35,40,105]. In brief, the bacteria were cultured in LB medium (1 L) for 12 h at 37°C with shaking at 180 rpm, and the supernatant from the culture was isolated by centrifugation at 10,000 × g for 10 min. The supernatant was then filtered twice through a 0.22 μm sterile filter to remove any remaining bacteria. The crude EVs were collected by ultracentrifugation (200,000 × g, 2 h, 4°C) with a 50.2 Ti rotor (Beckman-Coulter), and then resuspended in 1 mL of TE buffer. OptiPrep (Sigma-Aldrich) gradient centrifugation was used to purify the EVs. The crude EVs were suspended in 45% OptiPrep medium and added to the bottom of an OptiPrep gradient ranging from 25% to 40% (final % of OptiPrep, v/v), and ultracentrifuged (200,000 × g, 16 h, 4°C) with an sw41 Ti rotor (Beckman-Coulter). After centrifugation, 10 fractions were collected from the top to the bottom of the gradient, and the EVs visualized with transmission electron microscopy (TEM; Hitachi H7650, Japan). The fractions enriched in EVs were combined and ultracentrifuged at 200,000 ×g for 2 h at 4°C with a 70.1 Ti rotor (Beckman-Coulter) to isolate the purified EVs. The purified EVs were resuspended in 1.0 mL of TE buffer, and the protein concentration of the EVs was estimated with the BCA Protein Assay Kit (Thermo Scientific). The samples were stored at −80°C.

### Characterization of ExPEC EVs

TEM and cryo-TEM were used to visualize the ExPEC EVs. For TEM, the ExPEC strains (FY26, CBE59, and CFT073) were cultured on agar plates until logarithmic phase. The bacteria and EVs were diluted with ultrapure water and transferred to copper TEM grids, and the samples were negatively stained with phosphotungstic acid (2%, w/v). The samples were observed with a Hitachi H-7650 transmission electron microscope (Hitachi, Japan). For cryo-TEM, the ExPEC EVs were diluted with ultrapure water and transferred to a glow-discharged (10 mA for 120 s) carbon-coated grid (Quantifoil Cu R 2/2). The samples were blotted with filter paper in an FEI Vitrobot sample vitrification unit at 4°C and 100% humidity, and then quickly dropped into liquid ethane cooled by liquid nitrogen. The samples were observed with an electron microscope (Talos F200X FEI) operated at 200 kV at the temperature of liquid nitrogen. The morphologies and diameters of the ExPEC EVs were analyzed with the ImageJ software [106].

The particle size and concentration of ExPEC EVs were determined with NTA and DLS, respectively. For NTA, the EVs were diluted in cold PBS, and NTA was performed with a ZetaView S/N 18–373 analyzer (Particle Metrix, Germany) and the corresponding software (ZetaView 8.04.02). The average reads were calculated and plotted as numbers of particles per mL against the particle size [107]. For DLS, the EVs were diluted in cold PBS, and DLS was performed with a Nicomp nano Z3000 Zeta Potential Analyzer (Particle Sizing System, USA) at 25°C. The diameters of the EVs were determined and analyzed with the ZPW388 V2.13 software. All tests were repeated three times.

### Label-free quantitative proteomic analysis

Whole-bacterial pellets were washed once with PBS, and then treated with tosyl-l-lysyl-chloromethane hydrochloride (5 mM) and protease inhibitor cocktail (1/100 dilution; Sigma-Aldrich). After the cells were lysed with 2% Triton X-114, they were precipitated with 15% trichloroacetic acid (TCA) to form pellets. The whole-bacterial pellets were dissolved in 2.5% SDS at 100°C for 10 min. The dissolved samples were sonicated for 5 min and centrifuged at 13,000 × g for 10 min to eliminate any insoluble particles. The WCLs were treated with a second round of sonication, and the supernatant was transferred to a new tube to quantify the protein concentration with a BCA protein assay kit.

The peptides were then acidified with an equal volume of 1% formic acid and desalted with a C18 cartridge. The shotgun proteomic analyses were performed with an EASY-nLC 1200 UHPLC system (Thermo Fisher Scientific) and a Q Exactive HF-X mass spectrometer (Thermo Fisher Scientific). Briefly, the total peptides (2.0 μg) were separated with a two-column chromatography setup consisting of a home-made C18 nano-trap column and a home-made analytical column. Sixty-minute linear gradients were run by altering buffer B (0.1% formic acid in 80% acetonitrile) at 600 nL/min. The Q-Exactive HF-X mass spectrometer was used in positive polarity mode, with a complete MS scan range of 350–1,500 m/z and a resolution of 60,000 (maximum ion injection time, 20 ms; and automatic gain control [AGC] at 3 × 10^6^). A higher energy collisional dissociation (HCD) fragment analysis was performed (15,000 resolution; maximum ion injection time, 45 ms; and AGC at 1 × 10^5^).

### Mass spectrometry data and bioinformatics analysis

Proteome Discoverer 2.2 (PD 2.2, Thermo Fisher Scientific) was used to identify the proteins, and searches were performed against the EV proteomes of ExPEC strains FY26, CBE59, and CFT073 and the proteome of the FY26 WCL. These proteomic data have been submitted to the ProteomeXchange Consortium (http://proteomecentral.proteomexchange.org) under accession number PXD020207 via the iProX partner repository. Searches were set to include the modifications: fixed cysteine carbamidomethylation, variable methionine oxidation, and protein N-terminal acetylation. The maximum trypsin cleavage allowed two missed cleavages. The false discovery rate (FDR) for protein and peptide identification was < 1.0% and at least two unique peptides were identified. For label-free quantitation, we used precursor quantification based on intensity. We used the Mann–Whitney test to analyze the quantitative protein results statistically. To screen for differentially expressed proteins, the significance ratio was defined as *P* < 0.05.

The subcellular localization (SCL) of the identified proteins was predicted with different SCL prediction tools for bacterial proteins: CELLO 2.5 [108] and PSORTb version 3.0.2 [109]. The InterProScan 5 program was used for the Gene Ontology (GO) analysis [110]. The pathways and families of the identified proteins were analyzed with the Kyoto Encyclopedia of Genes and Genomes (KEGG) and Clusters of Orthologous Groups of proteins (COG) databases. The results of the PCA used for the cluster analysis of samples were visualized with ggplot2 and ggfortify [107]. Probably interacting proteins were predicted with the STRING-db server [109]. The enrichment analysis of KEGG, IPR, and GO was performed with the enrichment pipeline [111].

### Extraction and quantification of EV-associated DNA

Bacterial EVs were treated with 100 ng/mL DNase (TakaRa, 5 units/mg) to remove any DNA located outside the EVs and with 100 μg/mL proteinase K (PK; Sigma-Aldrich, 30 units/mg) to digest any extravesicular proteins. The DNase was then inactivated [112]. The PK/DNase-treated and untreated EV samples were then lysed with 0.125% Triton-X-100 at 37°C. The DNA was purified with phenol–chloroform extraction and precipitated with 100% ethanol and 0.3 M Na acetate (pH 5.0). The DNA concentrations were measured with a Spark Cyto microplate reader (Tecan), and the total DNA in the EVs was detected with 5% nondenaturing polyacrylamide gel electrophoresis. The results were visualized with the Tanon 1600 Gel Imaging System.

### Genomic sequencing of ExPEC EVs

For Illumina sequencing, at least 1 μg of FY26 EV genomic DNA was used to construct a sequencing library. A paired-end library with an insert size of ~400 bp was constructed according to the manufacturer’s instructions (Illumina). The draft genomic sequence for the FY26 EVs was acquired by sequencing 150-bp paired-end reads on an Illumina NovaSeq 6000 instrument. The functions of the FY26 EV gene-encoded proteins were predicted with the COG database. Finally, the draft genomic sequence of the FY26 EVs was compared with the genomic sequence of strain FY26.

### ExPEC prophage genome sequence analysis

The putative prophage genes in the ExPEC genome were predicted with PHAST [53]. The partial sequences of the ExPEC prophages encoding endolysins and holins were aligned.

### Detection of ExPEC proteins with western blotting

Antibodies directed against the following antigens were prepared and purified by Shanghai Willget Biotechnology Co., Ltd: outer-membrane proteins (OmpA, lipoprotein Lpp, and Pal), inner-membrane proteins (AdhE and AtpD), cytoplasmic proteins (Crp, acetate kinase AckA, phosphoglycerate kinase Pgk, and pyruvate kinase Pyk), moonlighting cytoplasmic protein (Gapdh), and cytoplasmic ribosomal proteins (RP-L2, RP-L15, RP-S3, RP-S5). Peptides based on the amino acid sequences of the membrane and cytoplasmic proteins were designed and synthesized. The antibodies were obtained by immunizing rabbits with the peptides, and were purified on antigen affinity columns. And the specificity of these antibodies was verified by western blotting. The peptide sequences are shown in S6 Table. The anti-endolysin antibodies (directed against Epel1 and the Epel2.1/Epel2.2 variants) were prepared and purified as described above, and the peptide sequences are shown in S6 Table.

Bacterial EVs and EV-free extracellular medium were prepared as previously described, with slight modifications [113]. Briefly, the EVs produced by strains FY26, CFT073, and CBE59 cultured in 1 L of LB medium were isolated with ultracentrifugation and DGU, and resuspended in 1.0 mL of 20 mM Tris-HCl (pH 8.0). The supernatant lacking EVs was collected after ultracentrifugation and concentrated 500-fold with Vivaspin 20 concentrators (3 kDa cutoff). To detect membrane and cytoplasmic proteins, EV preparations (5 μg of protein/lane), EV-free extracellular medium (5 μg of protein/lane), and WCL samples (5 μg of protein/lane) were analyzed with western blotting and antibodies directed against the proteins described above. Briefly, the EVs, EV-free extracellular medium, and bacterial WCLs were subjected to SDS-PAGE and then electrically transferred to polyvinylidene difluoride (PVDF) membranes (Millipore). After the membranes were blocked with 5% nonfat milk in PBS containing Tween 20 (PBST) for 1 h at room temperature, they were incubated overnight at 4°C with the primary antibodies described above. After the membranes were washed with PBST, they were incubated with horseradish peroxidase (HRP)-conjugated anti-rabbit IgG antibody (Abcam) for 1 h at room temperature. After the PVDF membranes were washes three times with PBST, the bands on the membranes were detected with High-sig ECL Western Blotting Substrate (Tanon, China) and the Tanon 5200 Chemiluminescent Imaging System (Tanon). The expression levels of the membrane and cytoplasmic proteins in the EVs and EV-free extracellular media were determined with western blotting. The protein expression of endolysins Epel1 and Epel2 in the ExPEC WCLs and EVs was also determined with western blotting.

The ExPEC EVs were fractionated and purified with OptiPrep DGU, and the protein concentration in each fraction (F1–F10) was estimated with a Pierce BCA Protein Assay Kit (Thermo Scientific). SDS-PAGE was used to visualize the protein profiles of the EVs in the 10 gradient fractions. The gel was loaded with 15 μL of each fraction (F1–F10). The control was 5 μg of total protein from each WCL sample. The gradient fractions (F1–F10, 15 μL/lane) were analyzed by immunoblotting with the corresponding antibodies. Dissociation assays and PK assays were performed as previously described [28,113], and EVs were lysed with 0.1 M EDTA. In the PK assay, purified ExPEC EVs (20 μg), either intact or lysed with 0.1 M EDTA (2 h, 37°C), were treated with 100 μg/mL PK to digest any extravesicular protein, as described previously [114]. The samples (10 μL) were analyzed with immunoblotting with the corresponding antibodies. Moreover, purified ExPEC EVs were analyzed by immunoblotting with anti-GFP antibodies (Sangon, D110008-0025).

### Immunofluorescence microscopy

To generate polyclonal mouse anti-EV sera, mice were immunized with the EVs of the three ExPEC strains, as previously described [35,115,116]. Briefly, the mice were subcutaneously immunized with EVs mixed with an equal volume of Montanide ISA 206 (Seppic, France), and the anti-EV serum was obtained 1 week after the second immunization. The mouse anti-EV primary antibodies were directly labeled with the fluorescent dye TRITC (Xi’an Qiyue Biotechnology Co., Ltd). The EVs were isolated and purified from the ExPEC strains that contained the GFP-expressing plasmid. The ExPEC strains were cultured in 1 L of LB medium supplemented with or without antibiotics. To stain the ExPEC EVs, the TRITC-labeled primary anti-EV antibodies were incubated with the ExPEC EVs overnight at 4°C. The EVs were washed twice in 100-kDa ultrafiltration units (Millipore, USA) to remove any free antibody. After staining, the EVs were plated on a glass slide and left to drying. A drop of 50% glycerol was added on the glass slide. Then, glass slide was covered using a cover slide. Finally, the labeled EVs were visualized with confocal microscopy (Nikon A1, Japan).

To visualize the intracellular ExPEC EVs in macrophages after phagocytosis, immunofluorescence assays were performed, as previously described [28,117], with slight modifications. Briefly, HD11 cells were incubated with EVs (10 μg) for 1 h. The cell supernatant was discarded, and the cells were washed with PBS and then incubated in DMEM for 7 h. The infected cells were washed with PBS at 8 h, fixed with 4% paraformaldehyde, and incubated with 0.1% Triton X-100 in PBS for 3 min to permeabilize them. The cells were blocked with 5% bovine serum albumin (BSA) in PBS and incubated with mouse polyclonal anti-EV antibody, and then with fluorescein isothiocyanate (FITC)-conjugated goat anti-mouse IgG antibody (EarthOx, USA) at 37°C for 1 h. The cells were then incubated with Phalloidin-iFluor 647 Conjugate (AAT Bioquest, USA) and 4′,6-diamidino-2-phenylindole (DAPI) at 37°C for 30 min. After the samples were washed three times with PBS, they were observed with confocal microscopy (Nikon A1).

### RNA isolation and quantification

To analyze the transcription levels of the endolysin genes, the total RNA of the ExPEC strains was extracted with a bacterial RNA kit (Omega Bio-Tek, China). The RNA was reverse transcribed to cDNA with a PrimeScript RT reagent Kit (TaKaRa), according to the instruction manual.

qPCR was performed with SYBR Green PCR Master Mix [35], and the primers for qPCR are described in S5 Table. The qPCR data from three independent reactions were analyzed with the ΔΔC_T_ method. Data are presented as the fold changes in transcript levels (normalized to the housekeeping gene *dnaE*) relative to a reference sample [118]. This assay was performed at least three times.

### Electrophoretic mobility shift assay (EMSA)

EMSAs were conducted to determine the binding of LexA to DNA probes containing the *epel1*, *epel2*.*1*, and *epel2*.*2* promoters [59]. To generate the DNA probes, the sequences (200 bp) of the *epel1*, *epel2*.*1*, and *epel2*.*2* promoter regions containing a putative LexA-binding site and the negative control DNA fragment (*pal*-coding region) were amplified with PCR (the primers are listed in S5 Table). The PCR products were purified with a Gel Extraction Kit (Omega Bio-Tek, USA). DNA fragments containing the mutated promoters of *epel1*, *epel2*.*1*, and *epel2*.*2*, with a nucleotide deletion in each promoter region (nt −68 to −53, nt −238 and −223, and nt −219 to −204, respectively) were prepared with fusion PCR. EMSAs were performed by adding increasing amounts of LexA protein (0–3 μg) to a DNA probe (50 ng) in the binding reaction mixture. The binding mixtures were incubated for 30 min at room temperature, and then subjected to 6% native polyacrylamide gel electrophoresis in 0.5 × TBE buffer at 100 V. The gels were stained with 1 × SYBR Gold Nucleic Acid Gel Stain (Thermo Fisher) in 0.5 × TBE for 30 min and visualized with the Tanon 1600 Gel Imaging System (Tanon).

### Cytotoxicity analysis

To investigate the effects of the interaction between EVs and macrophages (HD11 and THP-1 cells), cell viability was measured with Cell Counting Kit-8 (CCK-8; KeyBionet, Nanjing, China), according to the manufacturer’s protocol. HD11 or THP-1 cells in triplicate wells were incubated with 50 μg/mL of EVs from each ExPEC strain, and 10 μL of CCK-8 solution was added at 2, 4, 8, 16, or 24 hpi. PBS without EVs was used as the negative control. The cells were incubated in the dark at 37°C for 1 h, and the absorbance at 450 nm was detected. The toxicity of the ExPEC EVs towards macrophages (%) was calculated as: (sample well − blank well) / (negative well − blank well) × 100%.

### Cytokine ELISA

According to the manufacturer’s instructions, the cytokines (IL-1β, IL-6, IL-8, IL-12β, and TNF-α) from the cell-free supernatant of THP-1 macrophages was measured using the commercial cytokine ELISA kits, including IL-1β human ELISA Kit (Abcam, KAC1211), IL-6 Human ELISA Kit (Abcam, ab100712), IL-8 Human ELISA Kit (Abcam, BMS213-2), IL-12β human ELISA Kit (Abcam, BMS2013TEN), and TNF-α human ELISA Kit (Abcam, KHC3014C) [119]. Data were obtained from four individual assays, and each assay was carried with 3 biological repetitions.

### Detection of mitochondrial membrane potential

The JC-1 Mitochondrial Membrane Potential Detection Kit was used to measure the cell mitochondrial membrane potential. BMDMs in triplicate wells were incubated with 50 μg/mL EVs from each ExPEC strain, and JC-1 dye was added at 0, 2, 4, 8, 12, or 24 hpi. PBS without EVs was used as the negative control. The cells were treated with JC-1 dye for 20 min at 37°C and then washed three times with JC-1 assay buffer. The fluorescence of the mitochondrial monomers and aggregates was determined with flow cytometry. All samples were tested in at least three individual experiments and were repeated three times.

### Detection of BMDM proteins with western blotting

EV-, PBS-, and staurosporine (STS)- treated BMDMs were harvested and lysed in RIPA buffer. The supernatants were collected after centrifugation at 10,000 xg for 20 min at 4°C, and then the samples were separated with SDS-PAGE and analyzed with immunoblotting with anti-cleaved caspase 3 antibody (CST, 9964), anti-BCL-XL antibody (CST, 2764), anti-MCL-1 antibody (CST, 5453), anti-tubulin antibody (Abmart, M30109M) and anti-COX4 antibody (Santa Cruz Biotech, sc-376731). The cytosolic and mitochondrial fractions of the BMDMs were isolated with a Mitochondrial Isolation Kit for Cultured Cells (Thermo Scientific), according to the manufacturer’s protocol. The cytosolic and mitochondrial fractions were separated with SDS-PAGE and analyzed with immunoblotting using an anti-cytochrome *c* antibody (CST, 11940).

### Statistical analysis

GraphPad Prism version 8.0 and SPSS version 16 were used for all data analyses. Data are given as means ± standard errors of the means (SEM), and were analyzed with one-way ANOVA, two-way ANOVA or student’s *t*-test. *P* values < 0.05 were considered to indicate statistical significance.

## Supporting information

S1 Fig Isolation and purification of ExPEC EVs from different ST strains. **(A)** Growth curves of three ExPEC strains. APEC strain FY26, APEC strain CBE59, and UPEC strain CFT073 were cultured in LB broth at 37°C. Bacterial growth was monitored by measuring the OD_600_. Two-way ANOVA was used to evaluate statistical significance (*P* > 0.05). The growth experiments were performed at least three times. **(B)** Protein concentrations in EV produced in different ExPEC strains. The EVs of avian pathogenic *E*. *coli* (APEC) strain FY26, APEC strain CBE59, and uropathogenic *E*. *coli* (UPEC) strain CFT073 were cultured for 4 h (early log phase of bacterial growth), 8 h (log phase), 12 h (early stationary phase), or 16 h (stationary phase), and then isolated from the cell-free supernatants by ultracentrifugation. **(C)** FY26 EVs were extracted and pelleted by ultracentrifugation. **(D)** FY26 EVs were observed with transmission electron microscopy (TEM). Scale bars: 200 nm. **(E)** EVs were purified with density gradient ultracentrifugation (DGU). Particle samples were successfully separated, and the tubes were labeled as the 10 fractions (F1–F10). **(F)** The protein concentration of each fraction (F1–F10) was measured with a BCA kit. **(G)** The size distributions and concentrations of the three purified EVs were detected with a nanoparticle tracking analysis (NTA).
(TIF)

S2 Fig Proteomic analysis of EVs derived from ExPEC strains. **(A)** ExPEC EVs from different density gradient fractions in CBE59 were analyzed with SDS-PAGE. Fractions (15 μL loaded onto the gel) are numbered according to increasing density. The images show one representative experiment. M: Protein marker; WCL: 5 μg loaded into each well of the gel. **(B)** ExPEC EVs from different density gradient fractions in CFT073 were analyzed with SDS-PAGE. **(C)** Functional classification of EV unigenes identified in the three ExPEC strains with Clusters of Orthologous Groups of proteins (COG). The EV proteins were classified into 23 COG categories. **(D)** Functional classification of EV proteins identified in three ExPEC strains was analyzed with the Kyoto Encyclopedia of Genes and Genomes (KEGG). **(E)** The abundances of the 300 most-abundant proteins isolated from the CFT073 EV fractions were compared with the total protein abundance in the FY26 EVs. Subcellular localization is shown as follows: outer-membrane proteins (light blue), periplasmic proteins (purple), inner-membrane proteins (red), cytoplasmic proteins (deep blue), moonlighting proteins (green), and ribosomal subunit proteins (orange). Proteins enriched in the FY26 EVs are shown below and to the right of the dashed line, and proteins depleted in the EVs are shown above and to the left of the line. The data for this figure can be found in S1 Table. **(F)** The abundance of the 300 most-abundant proteins isolated from the CBE59 EV fractions was compared with their abundances in the FY26 EVs. **(G)** The abundances of the 300 most-abundant proteins isolated from the CBE59 EVs fractions were compared with their abundances in the CFT073 EVs.
(TIF)

S3 Fig The specificity of the antibodies corresponding to the membrane or cytoplasmic proteins in *E*. *coli* was verified by western blotting. The mutant strains for these genes (*ompA*, *lpp*, *pal*, *adhE*, *atpD*, *crp*, *pgk*, *pyk*, *ackA*, L15, *epel1*, and *epel2*.*1*/*epel2*.*2*) were constructed in WT strain FY26.The membrane or cytoplasmic proteins in WT FY26 or mutant strains (except GAPDH, L2, S3, and S5) was determined with western blotting using these antibodies prepared in this study. As expected, the bands of these membrane or cytoplasmic proteins could be detected in WT FY26, and not in the mutants.
(TIF)

S4 Fig To detect the protein cargoes within ExPEC EVs. **(A)** Expression of membrane and cytoplasmic proteins in DGU-purified EVs (F1–F10) was determined with western blotting. **(a)** CBE59 and **(b)** CFT073. Nonfractionated EVs were used as the positive controls, and whole-cell lysates (WCLs) were used as the loading controls. Total protein (1 μg) was loaded into the OmpA and Gapdh lanes, and 5 μg of total protein was loaded into the other lanes. **(B)** Dissociation assays confirmed that the protein cargoes were tightly associated with the EVs of the ExPEC strains. **(a)** CBE59 and **(b)** CFT073. OptiPrep-purified EVs were treated with HEPES buffer containing the indicated chemical agents or with HEPES buffer only. The pellets (P; containing EVs) and extracellular media (S; containing proteins released from EVs) were collected by ultracentrifugation, and the samples were analyzed with western blotting. Total protein (1 μg) was loaded into the OmpA and Gapdh lanes, and 5 μg of total protein was loaded into the other lanes.
(TIF)

S5 Fig The size distribution and concentration of purified EVs produced by FY26 and FY26Δ*Pal* were determined. **(A)** The size distributions and concentrations of purified EVs produced by FY26 were determined with a nanoparticle tracking analysis (NTA). Data shown are the means ± SEM of three independent experiments. **(B)** The size distributions and concentrations of purified EVs produced by FY26Δ*Pal* were determined with a nanoparticle tracking analysis (NTA). **(C)** Protein concentrations in EV produced by FY26 and FY26Δ*pal* measured with a BCA kit.
(TIF)

S6 Fig The effects of endolysin genes deletion on the production of ExPEC EVs. **(A)** Genomic alignment of ExPEC (FY26, CBE59, and CFT073) prophages containing putative endolysin genes. Arrows denote the locations, relative sizes, and different functional categories of the genes. Scale bars: 5,000 bp. **(B)** Transcription levels of endolysin genes in ExPEC strains in different growth phases (4, 6, 8, 10, and 12 h) were determined with RT–qPCR. **(a)** CBE59 and **(b)** CFT073.–h, putative holin genes; -e, putative endolysin genes. Data shown are the means ± SEM of three independent experiments relative to the housekeeping gene *dnaE*. Statistical significance was evaluated with one-way ANOVA (**P < 0.01). **(C)** Protein levels of endolysins (Epel1 and Epel2 variants) in whole-cell lysates of ExPEC strains were determined with western blotting. **(D)** The concentrations of purified EVs produced by several mutants were determined with a nanoparticle tracking analysis (NTA). **(E)** Protein levels of Epel2 variants in complemented strains were determined with western blotting. **(F)** The concentrations of purified EVs produced by FY26C*epel1*, FY26C*epel2*.*1*, and FY26C*epel2*.*2* were determined with NTA. **(G)** Protein concentrations in EVs produced by FY26C*epel1*, FY26C*epel2*.*1*, and FY26C*epel2*.*2* were measured with a BCA kit. **(H)** Total protein per 1 L of bacterial supernatant from wild-type (WT) FY26 and complemented strains (FY26C*epel1*, FY26C*epel2*.*1*, and FY26C*epel2*.*2*) was measured with BCA. **(I)** Levels of membrane and cytoplasmic proteins EV-free extracellular medium of WT FY26 and complemented strains (FY26C*epel1*, FY26C*epel2*.*1*, and FY26C*epel2*.*2*) were determined with western blotting.
(TIF)

S7 Fig Putative prophage genes of ExPEC strains. **(A)** FY26; **(B)** CBE59; **(C)** CFT073.
(TIF)

S8 Fig Sequence comparison of endolysin genes from FY26, CBE59, and CFT073. (TIF)

S9 Fig RecA/LexA-dependent SOS response controls the expression of ExPEC endolysin genes. **(A)** Size distribution and concentration of purified EVs produced by FY26Δ*lexA* and FY26Δ*recA* were determined with a nanoparticle tracking analysis (NTA). **(B)** Protein concentrations in EVs produced by FY26Δ*lexA* were measured with a BCA kit. **(C)** Transcription levels of *epel1*, *epel2*.*1*, and *epel2*.*2* in FY26 and FY26Δ*lexA* in different growth phases (4, 6, 8, 10, and 12 h) were determined with RT–qPCR. Data are shown as means ± SEM of three independent experiments relative to the housekeeping gene *dnaE*. Statistical significance was evaluated with two-way ANOVA (**P < 0.01). **(D)** Protein levels of endolysins Epel1 and the Epel2 variants in whole-cell lysates (WCLs) and the EVs of FY26Δ*lexA* were determined with western blotting. **(E)** Purification of LexA fusion proteins. Protein from the soluble fraction (lane 1) and insoluble fraction of the cell lysate (lane 2), and the purified fusion protein (lane 3) were detected with SDS-PAGE with Coomassie Brilliant Blue staining. M: protein marker. **(F)** Protein concentrations in EVs produced by wild-type (WT) FY26 and mutant FY26Δ*recA* were measured with a BCA kit. The strains were exposed to sublethal concentrations of H_2_O_2_ or cultured under routine conditions. **(G)** Transcription levels of *epel1*, *epel2*.*1*, and *epel2*.*2* in FY26 and FY26Δ*recA* in different growth phases (4, 6, 8, 10, and 12 h) were determined with RT–qPCR. Data are shown as means ± SEM of three independent experiments relative to the housekeeping gene *dnaE*. Statistical significance was evaluated with two-way ANOVA (**P < 0.01). **(H)** Size distribution and concentration of purified EVs produced by FY26, FY26Δ*recA* and FY26Δ*epel1*/*2*.*1*/*2*.*2* were determined with a nanoparticle tracking analysis (NTA). The strains were exposed to sublethal concentrations of H_2_O_2_. **(I)** Protein levels of endolysins (Epel1 and Epel2 variants) in the WCLs and EVs of WT FY26 and FY26Δ*recA* were determined with western blotting. WT FY26 and mutant FY26Δ*recA* were cultured in LB medium supplemented with sublethal concentrations of H_2_O_2_. **(J)** The concentrations of purified EVs produced by mutant FY26Δ*epel1*/*2*.*1*/*2*.*2* were determined with NTA. The strains were exposed to sublethal concentrations of H_2_O_2_ or cultured under routine conditions. **(K)** Protein concentrations in EVs produced by mutant FY26Δ*epel1*/*2*.*1*/*2*.*2* were measured with a BCA kit.
(TIF)

S10 Fig The effect of *ftsK* deletion on the production of ExPEC EVs. **(A)** Size distribution and concentration of purified EVs from FY26Δ*ftsK*, FY26C*ftsK*, FY26Δ*ftsK-*pSTV28*-sul1* and FY26Δ*ftsK*/*recA* were determined with NTA. **(B)** Protein concentrations in EVs produced by FY26Δ*ftsK*, FY26C*ftsK* and FY26Δ*ftsK*/*recA* were measured with a BCA kit. **(C)** Transcription levels of *epel1*, *epel2*.*1*, and *epel2*.*2* in FY26, FY26Δ*ftsK*, FY26C*ftsK* and FY26Δ*ftsK*/*recA* in different growth phases (4, 6, 8, 10, and 12 h) were determined with RT–qPCR. Data are shown as means ± SEM of three independent experiments relative to the housekeeping gene *dnaE*. Statistical significance was evaluated with two-way ANOVA (**P < 0.01). **(D)** Protein levels of endolysins Epel1 and Epel2 variants in whole-cell lysates (WCLs) of FY26Δ*ftsK* and FY26Δ*ftsK*/*recA* were determined with western blotting.
(TIF)

S11 Fig The effect of t6A deletion on the production of ExPEC EVs. **(A)** Size distribution and concentration of purified EVs from FY26Δt6A, FY26Ct6A, FY26Δt6A*-*pSTV28*-sul1* and FY26Δt6A/*recA* were determined with NTA. **(B)** Protein concentrations in EVs produced by FY26Δt6A, FY26Ct6A and FY26Δt6A/*recA* were measured with a BCA kit. **(C)** Transcription levels of *epel1*, *epel2*.*1*, and *epel2*.*2* in FY26, FY26Δt6A, FY26Ct6A and FY26Δt6A/*recA* in different growth phases (4, 6, 8, 10, and 12 h) were determined with RT–qPCR. Data are shown as means ± SEM of three independent experiments relative to the housekeeping gene *dnaE*. Statistical significance was evaluated with two-way ANOVA (**P < 0.01). **(D)** Protein levels of endolysins Epel1 and Epel2 variants in whole-cell lysates (WCLs) of FY26Δt6A and FY26Δt6A/*recA* were determined with western blotting.
(TIF)

S12 Fig The distribution and concentration of purified EVs in ExPEC EVs. **(A)** Size distributions and concentrations of purified EVs in FY26 cultured with sublethal concentrations of antibiotics were determined with a nanoparticle tracking analysis (NTA). FY26 strain was treated with sublethal doses of seven antibiotics. **(B)** Size distributions and concentrations of purified EVs in FY26Δ*recA* cultured with sublethal concentrations of antibiotics were determined with a nanoparticle tracking analysis (NTA). **(C)** Size distributions and concentrations of purified EVs in multidrug-resistant ExPEC strain ST95-32 cultured with relatively high concentrations of antibiotics.
(TIF)

S13 Fig The effect of antibiotics on the production of ExPEC EVs. **(A)** Protein concentrations of purified EVs in FY26 cultured with sublethal concentrations of ciprofloxacin were measured with a BCA kit. **(B)** Transcription levels of *epel1*, *epel2*.*1*, and *epel2*.*2* in FY26 and FY26Δ*recA* in various growth phases (4, 6, 8, 10, and 12 h) were determined with RT–qPCR. The strains were exposed to sublethal concentrations of antibiotics or cultured under routine conditions. Statistical significance was evaluated with two-way ANOVA (**P < 0.01). **(C)** Protein levels of endolysins Epel1 and the Epel2 variants in FY26 strain cultured with sublethal concentrations of antibiotics were determined with western blotting. **(D)** The concentrations of purified EVs in FY26Δ*recA* cultured with sublethal concentrations of antibiotics were determined with NTA. **(E)** Immunofluorescent staining of EVs produced by FY26Δ*recA* treated with antibiotics. **(F)** GFP and OmpA proteins in EVs were determined with western blotting. **(G)** The concentrations of purified EVs produced by mutant FY26Δ*epel1*/*2*.*1*/*2*.*2* were determined with NTA. The strains were exposed to sublethal concentrations of ciprofloxacin or cultured under routine conditions. **(H)** Immunofluorescent staining of the EVs produced by mutant FY26Δ*epel1*/*2*.*1*/*2*.*2*.
(TIF)

S14 Fig The effect of antibiotic on the DNA content carried by ExPEC EVs. **(A)** Total DNA in EVs was measured with microplate reader. Total DNA was extracted from equivalent numbers of EVs (7.1 × 10^11^ vesicles) from FY26 strain cultured with sublethal concentrations of antibiotics and measured with microplate reader. Data were obtained from at least three independent experiments with three replicates. Statistical significance was evaluated with one-way ANOVA (**P < 0.01). **(B-C)** Total DNA in the EVs from FY26 strain cultured with sublethal concentrations of antibiotics was visualized with nondenaturing polyacrylamide gel electrophoresis. Total DNA was isolated from equivalent numbers (7.1 × 10^11^) of PK/DNase-treated or untreated EVs from FY26 strain cultured with sublethal concentrations of antibiotics. ‘+’ indicates that samples were treated with PK and DNase I, and ‘−’ indicates that samples were not treated with PK or DNaseI. Naked DNA (pET-32a) was used as a control. **(D)** The concentrations of purified EVs in multidrug-resistant ExPEC strain ST95-32 cultured with relatively high concentrations of antibiotics (ampicillin, 100 μg/mL; ceftazidime, 50 μg/mL; levofloxacin, 50 μg/mL; chloramphenicol, 30 μg/mL) were determined with NTA.
(TIF)

S1 Table Proteins from FY26 EVs, CFT073 EVs, and CBE59 EVs identified with a label-free approach. (XLSX)

S2 Table Proteins from FY26 strain identified with a label-free approach. (XLSX)

S3 Table Endolysins, lysozymes, and holins derived from ExPEC phage. (XLSX)

S4 Table Bacterial strains and plasmids used in this study. (DOCX)

S5 Table PCR primers used in this study. (DOCX)

S6 Table Peptide sequences used in this study. (DOCX)
