# Supplementary figures and images for: Reductions in bacterial viability stimulate the production of Extra-intestinal Pathogenic Escherichia coli (ExPEC) cytoplasm-carrying Extracellular Vesicles (EVs)
Source: PLoS Pathog. 2022 Oct 19;18(10):e1010908. doi: 10.1371/journal.ppat.1010908 (PMC9621596; doi:10.1371/journal.ppat.1010908)

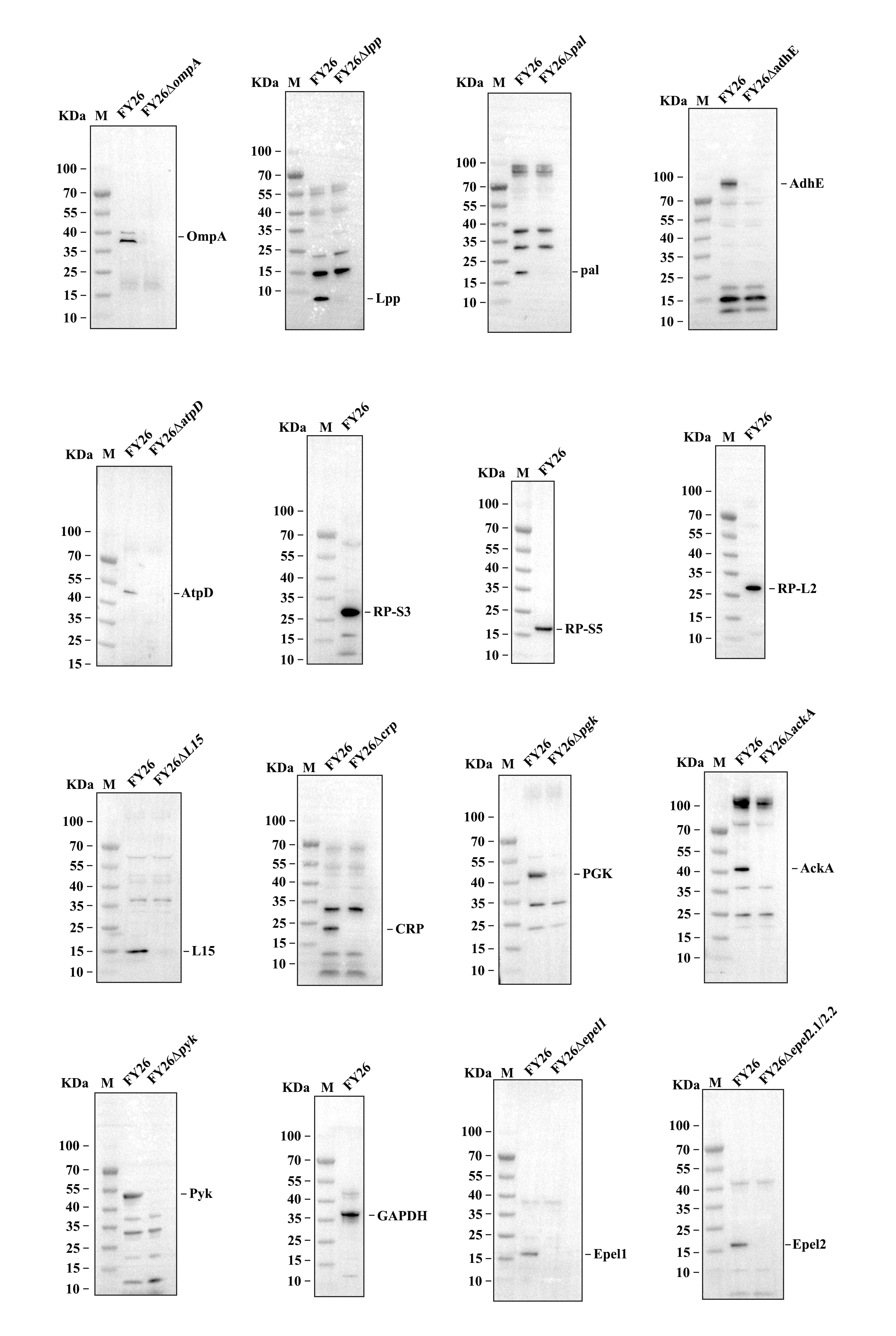

Supplement: S3 Fig — The mutant strains for these genes (ompA, lpp, pal, adhE, atpD, crp, pgk, pyk, ackA, L15, epel1, and epel2.1/epel2.2) were constructed in WT strain FY26.The membrane or cytoplasmic proteins in WT FY26 or mutant strains (except GAPDH, L2, S3, and S5) was determined with western blotting using these antibodies prepared in this study. As expected, the bands of these membrane or cytoplasmic proteins could be detected in WT FY26, and not in the mutants. (TIF) [file ppat.1010908.s003.tif]

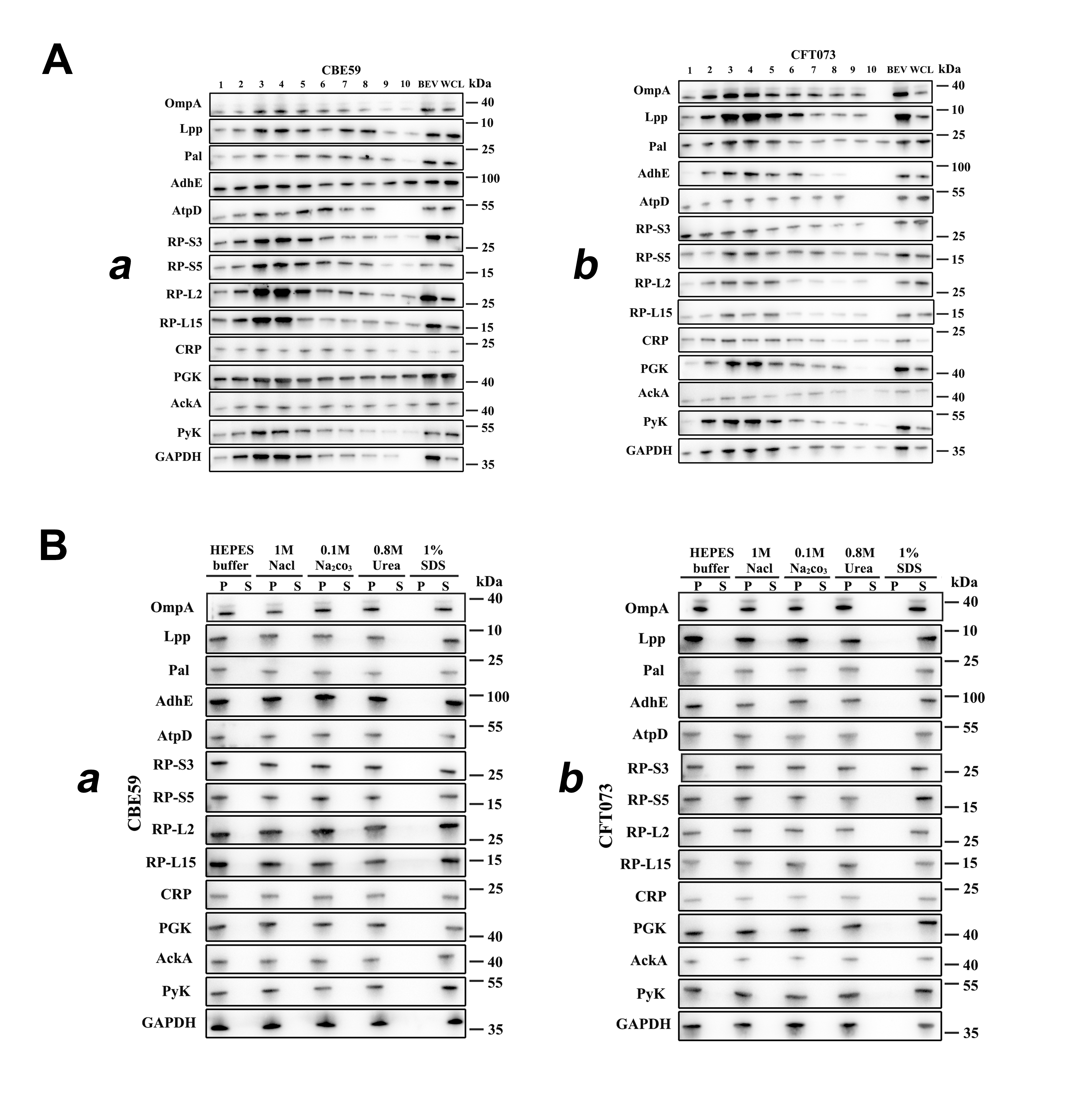

Supplement: S4 Fig — (A) Expression of membrane and cytoplasmic proteins in DGU-purified EVs (F1–F10) was determined with western blotting. (a) CBE59 and (b) CFT073. Nonfractionated EVs were used as the positive controls, and whole-cell lysates (WCLs) were used as the loading controls. Total protein (1 μg) was loaded into the OmpA and Gapdh lanes, and 5 μg of total protein was loaded into the other lanes. (B) Dissociation assays confirmed that the protein cargoes were tightly associated with the EVs of the ExPEC strains. (a) CBE59 and (b) CFT073. OptiPrep-purified EVs were treated with HEPES buffer containing the indicated chemical agents or with HEPES buffer only. The pellets (P; containing EVs) and extracellular media (S; containing proteins released from EVs) were collected by ultracentrifugation, and the samples were analyzed with western blotting. Total protein (1 μg) was loaded into the OmpA and Gapdh lanes, and 5 μg of total protein was loaded into the other lanes. (TIF) [file ppat.1010908.s004.tif]

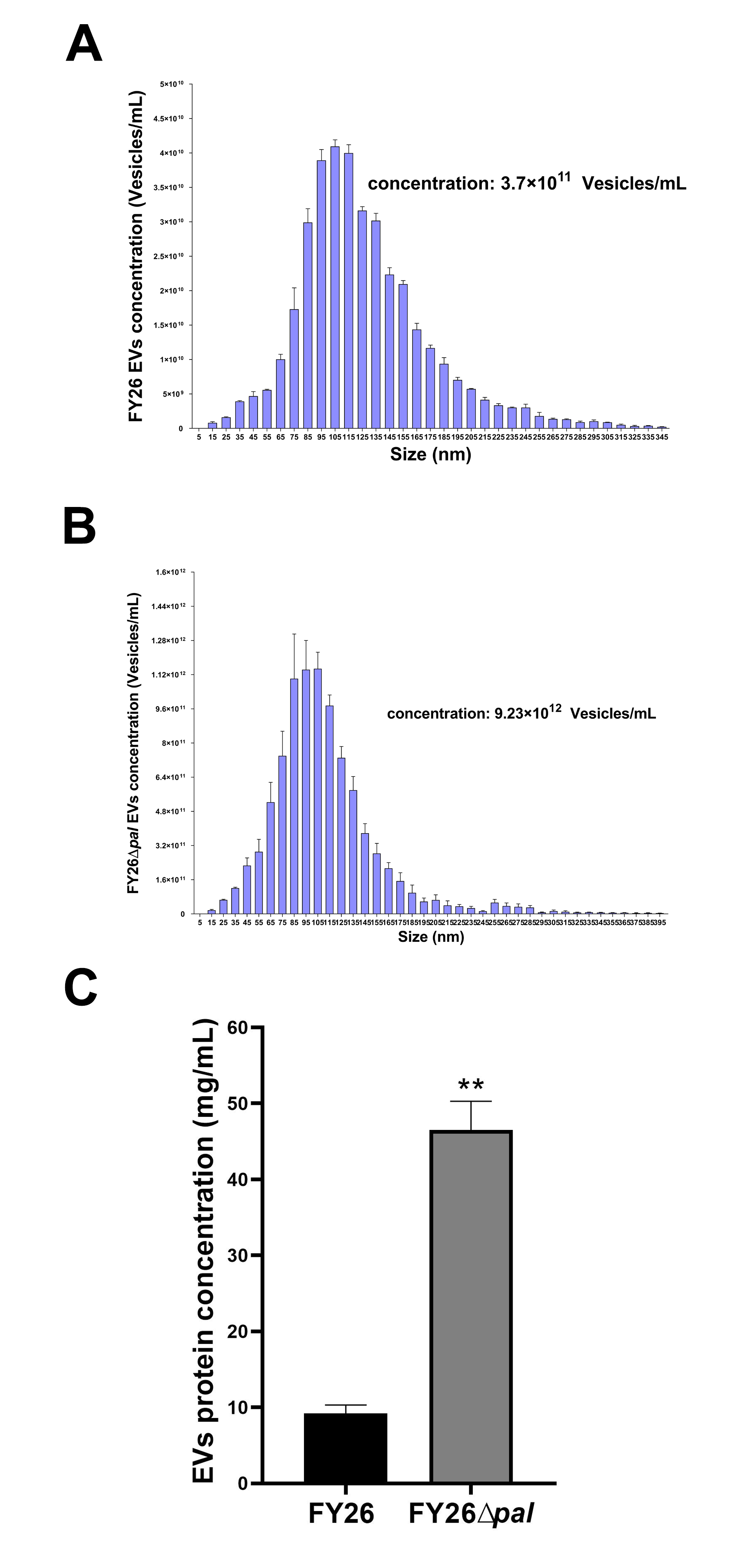

Supplement: S5 Fig — (A) The size distributions and concentrations of purified EVs produced by FY26 were determined with a nanoparticle tracking analysis (NTA). Data shown are the means ± SEM of three independent experiments. (B) The size distributions and concentrations of purified EVs produced by FY26ΔPal were determined with a nanoparticle tracking analysis (NTA). (C) Protein concentrations in EV produced by FY26 and FY26Δpal measured with a BCA kit. (TIF) [file ppat.1010908.s005.tif]

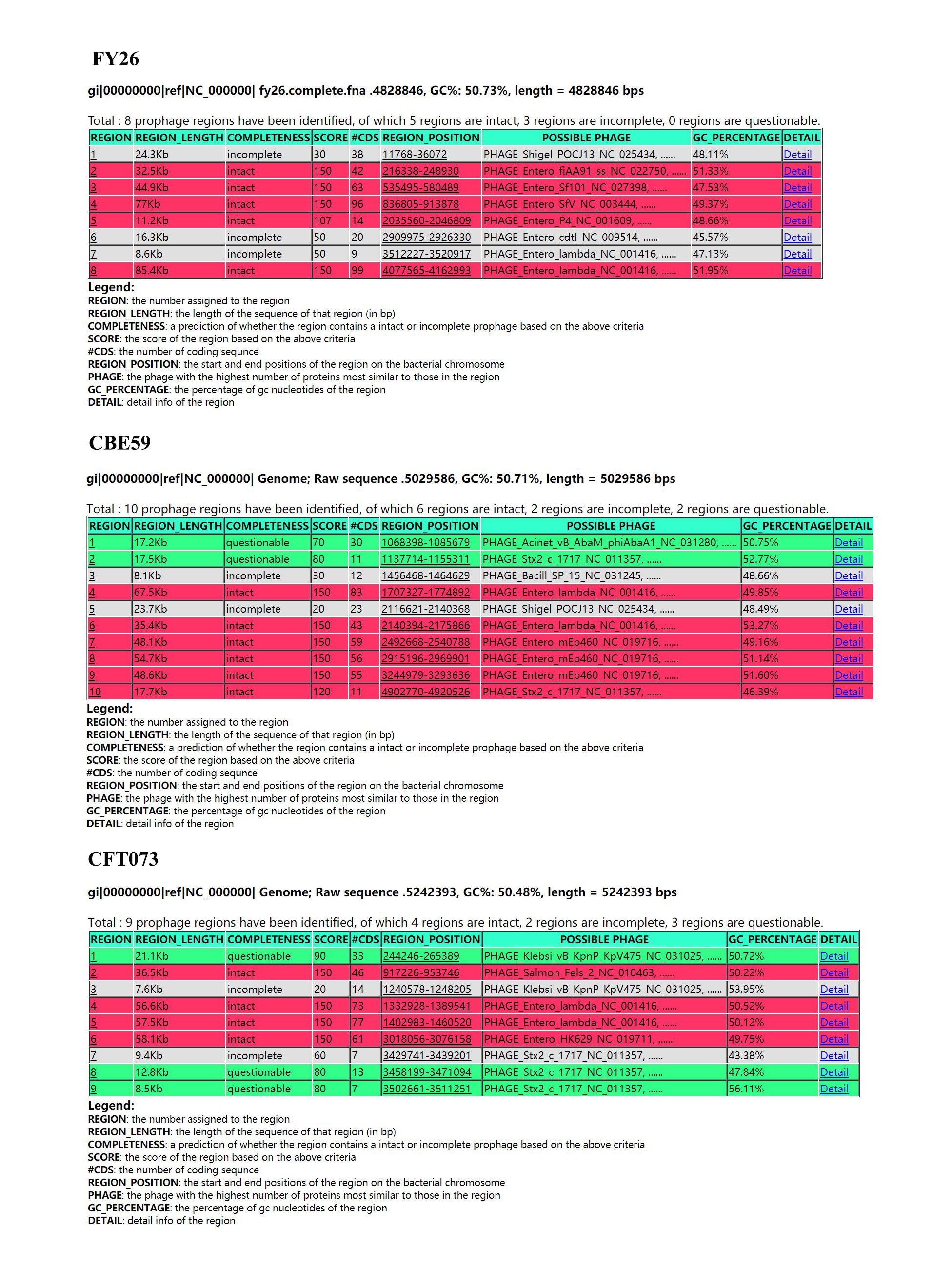

Supplement: S7 Fig — (A) FY26; (B) CBE59; (C) CFT073. (TIF) [file ppat.1010908.s007.tif]

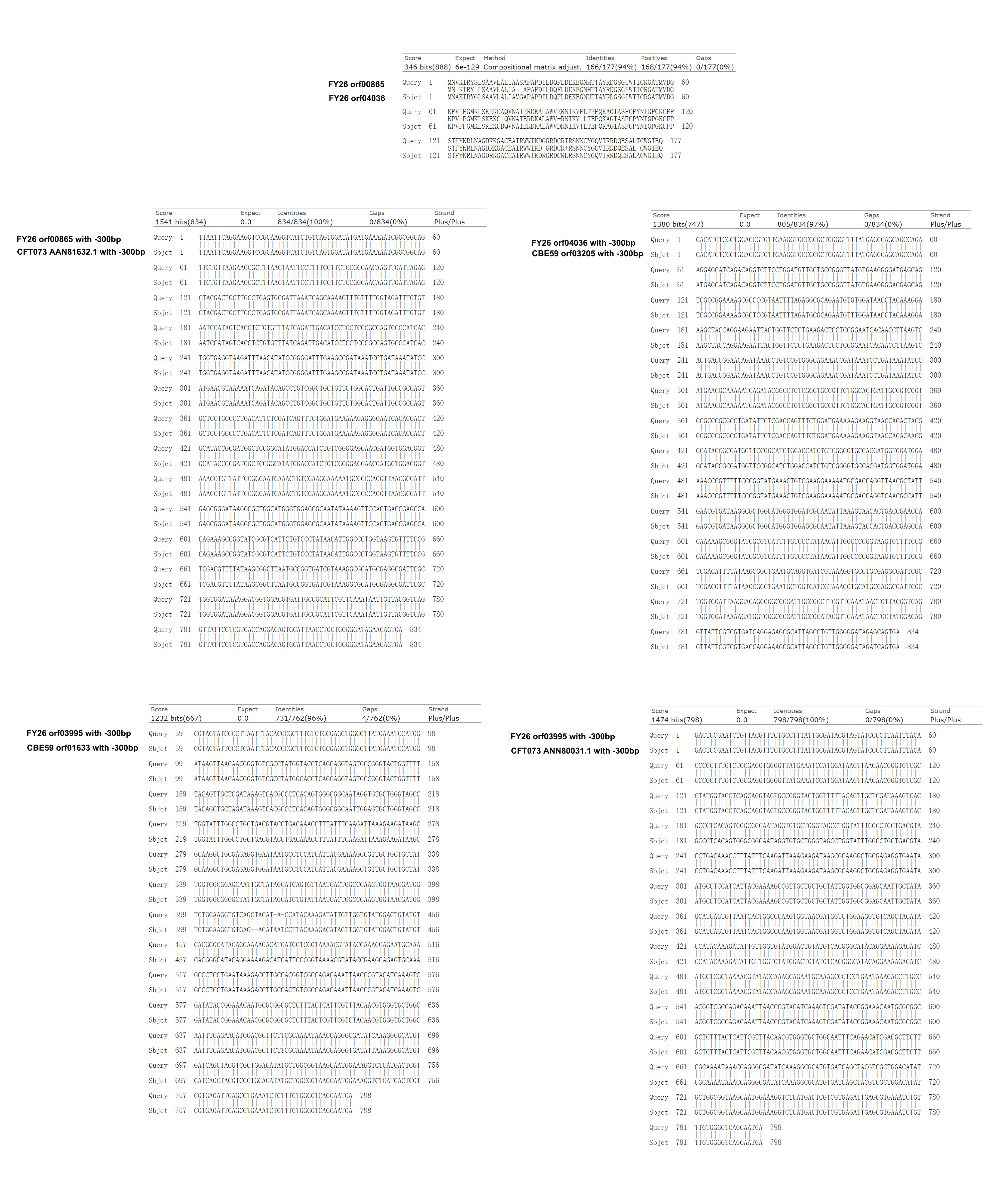

Supplement: S8 Fig — (TIF) [file ppat.1010908.s008.tif]

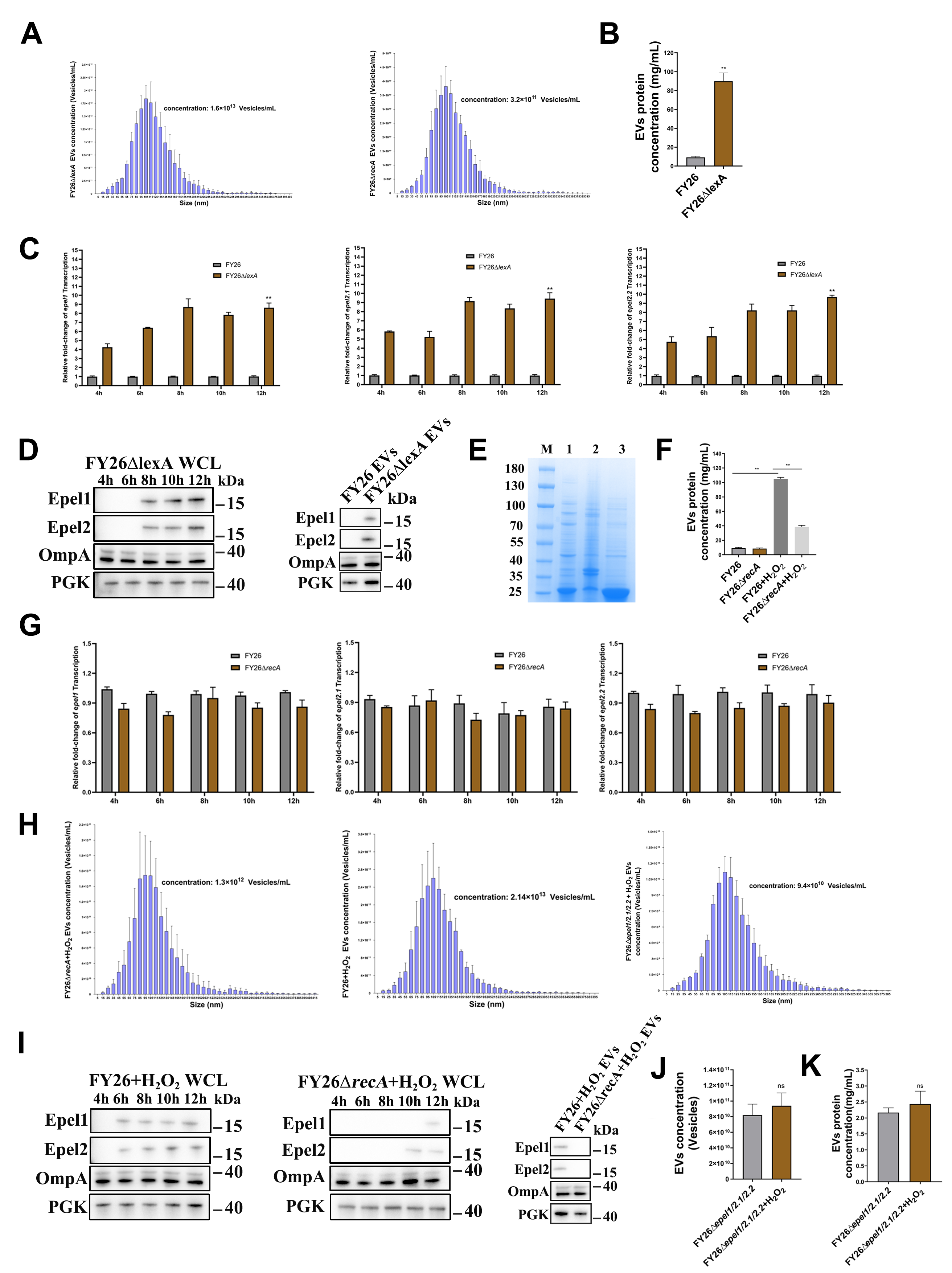

Supplement: S9 Fig — (A) Size distribution and concentration of purified EVs produced by FY26ΔlexA and FY26ΔrecA were determined with a nanoparticle tracking analysis (NTA). (B) Protein concentrations in EVs produced by FY26ΔlexA were measured with a BCA kit. (C) Transcription levels of epel1, epel2.1, and epel2.2 in FY26 and FY26ΔlexA in different growth phases (4, 6, 8, 10, and 12 h) were determined with RT–qPCR. Data are shown as means ± SEM of three independent experiments relative to the housekeeping gene dnaE. Statistical significance was evaluated with two-way ANOVA (**P < 0.01). (D) Protein levels of endolysins Epel1 and the Epel2 variants in whole-cell lysates (WCLs) and the EVs of FY26ΔlexA were determined with western blotting. (E) Purification of LexA fusion proteins. Protein from the soluble fraction (lane 1) and insoluble fraction of the cell lysate (lane 2), and the purified fusion protein (lane 3) were detected with SDS-PAGE with Coomassie Brilliant Blue staining. M: protein marker. (F) Protein concentrations in EVs produced by wild-type (WT) FY26 and mutant FY26ΔrecA were measured with a BCA kit. The strains were exposed to sublethal concentrations of H2O2 or cultured under routine conditions. (G) Transcription levels of epel1, epel2.1, and epel2.2 in FY26 and FY26ΔrecA in different growth phases (4, 6, 8, 10, and 12 h) were determined with RT–qPCR. Data are shown as means ± SEM of three independent experiments relative to the housekeeping gene dnaE. Statistical significance was evaluated with two-way ANOVA (**P < 0.01). (H) Size distribution and concentration of purified EVs produced by FY26, FY26ΔrecA and FY26Δepel1/2.1/2.2 were determined with a nanoparticle tracking analysis (NTA). The strains were exposed to sublethal concentrations of H2O2. (I) Protein levels of endolysins (Epel1 and Epel2 variants) in the WCLs and EVs of WT FY26 and FY26ΔrecA were determined with western blotting. WT FY26 and mutant FY26ΔrecA were cultured in LB medium supplemented with sublethal [file ppat.1010908.s009.tif]

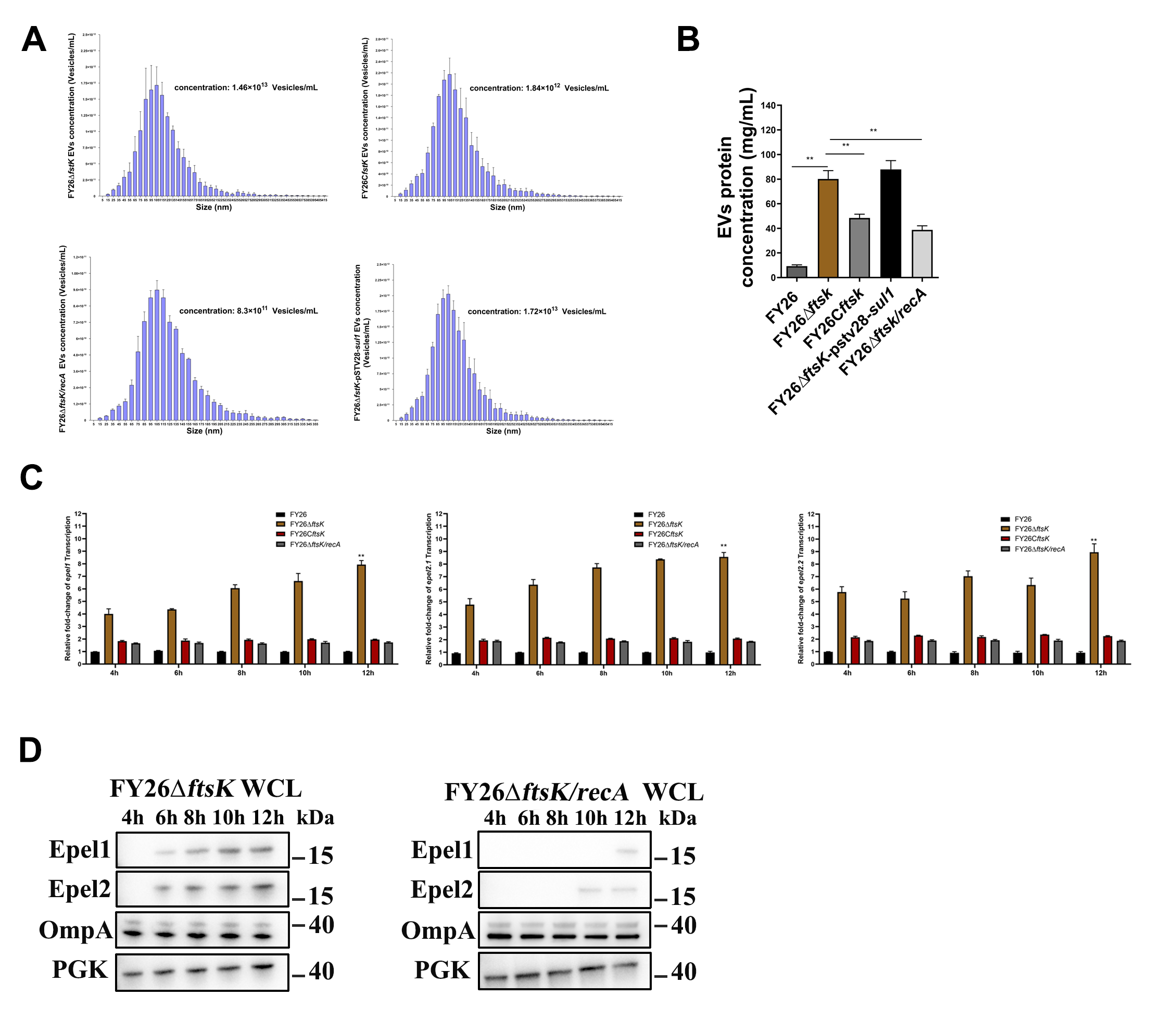

Supplement: S10 Fig — (A) Size distribution and concentration of purified EVs from FY26ΔftsK, FY26CftsK, FY26ΔftsK-pSTV28-sul1 and FY26ΔftsK/recA were determined with NTA. (B) Protein concentrations in EVs produced by FY26ΔftsK, FY26CftsK and FY26ΔftsK/recA were measured with a BCA kit. (C) Transcription levels of epel1, epel2.1, and epel2.2 in FY26, FY26ΔftsK, FY26CftsK and FY26ΔftsK/recA in different growth phases (4, 6, 8, 10, and 12 h) were determined with RT–qPCR. Data are shown as means ± SEM of three independent experiments relative to the housekeeping gene dnaE. Statistical significance was evaluated with two-way ANOVA (**P < 0.01). (D) Protein levels of endolysins Epel1 and Epel2 variants in whole-cell lysates (WCLs) of FY26ΔftsK and FY26ΔftsK/recA were determined with western blotting. (TIF) [file ppat.1010908.s010.tif]

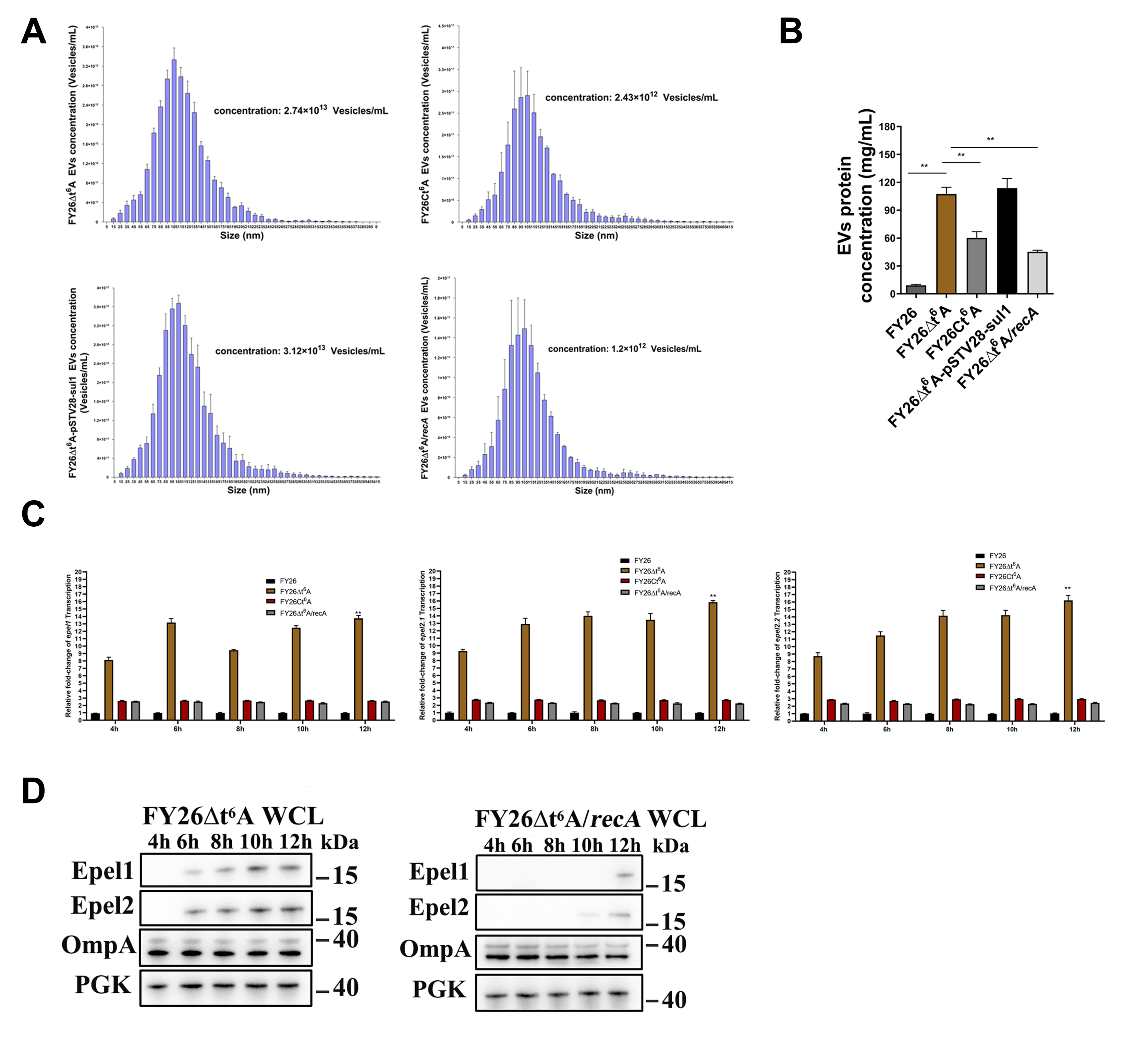

Supplement: S11 Fig — (A) Size distribution and concentration of purified EVs from FY26Δt6A, FY26Ct6A, FY26Δt6A-pSTV28-sul1 and FY26Δt6A/recA were determined with NTA. (B) Protein concentrations in EVs produced by FY26Δt6A, FY26Ct6A and FY26Δt6A/recA were measured with a BCA kit. (C) Transcription levels of epel1, epel2.1, and epel2.2 in FY26, FY26Δt6A, FY26Ct6A and FY26Δt6A/recA in different growth phases (4, 6, 8, 10, and 12 h) were determined with RT–qPCR. Data are shown as means ± SEM of three independent experiments relative to the housekeeping gene dnaE. Statistical significance was evaluated with two-way ANOVA (**P < 0.01). (D) Protein levels of endolysins Epel1 and Epel2 variants in whole-cell lysates (WCLs) of FY26Δt6A and FY26Δt6A/recA were determined with western blotting. (TIF) [file ppat.1010908.s011.tif]

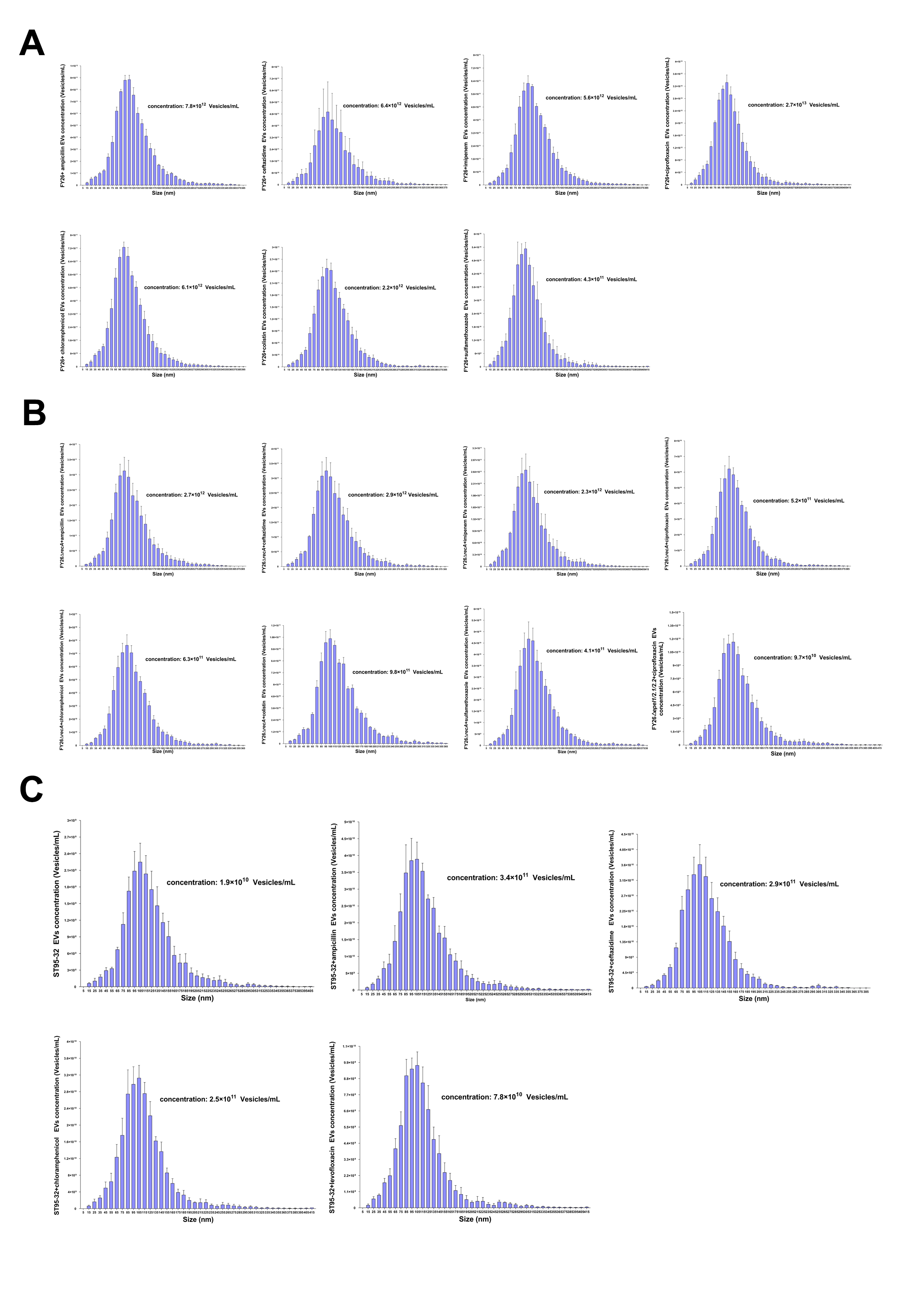

Supplement: S12 Fig — (A) Size distributions and concentrations of purified EVs in FY26 cultured with sublethal concentrations of antibiotics were determined with a nanoparticle tracking analysis (NTA). FY26 strain was treated with sublethal doses of seven antibiotics. (B) Size distributions and concentrations of purified EVs in FY26ΔrecA cultured with sublethal concentrations of antibiotics were determined with a nanoparticle tracking analysis (NTA). (C) Size distributions and concentrations of purified EVs in multidrug-resistant ExPEC strain ST95-32 cultured with relatively high concentrations of antibiotics. (TIF) [file ppat.1010908.s012.tif]

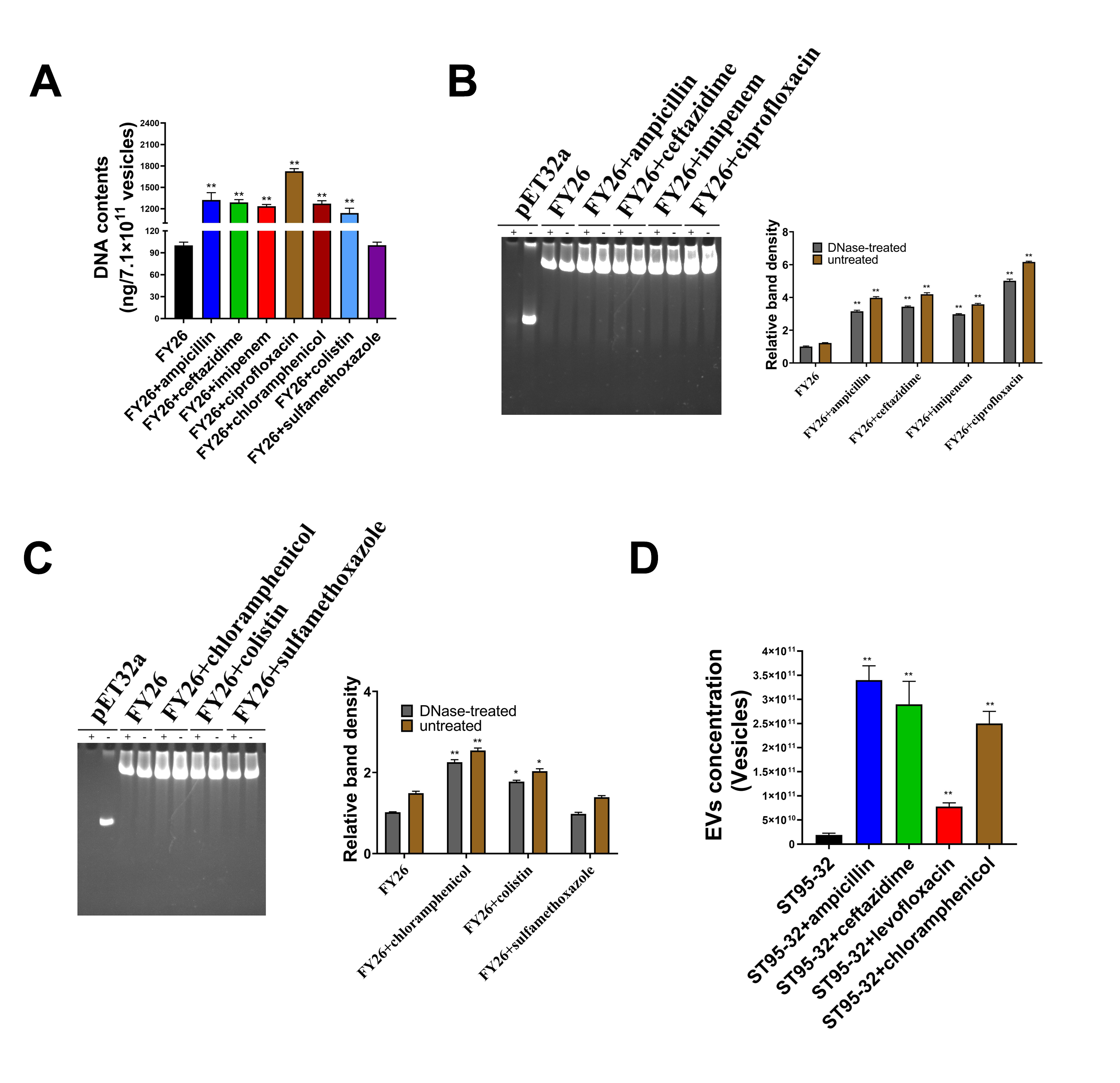

Supplement: S14 Fig — (A) Total DNA in EVs was measured with microplate reader. Total DNA was extracted from equivalent numbers of EVs (7.1 × 10^11^ vesicles) from FY26 strain cultured with sublethal concentrations of antibiotics and measured with microplate reader. Data were obtained from at least three independent experiments with three replicates. Statistical significance was evaluated with one-way ANOVA (**P < 0.01). (B-C) Total DNA in the EVs from FY26 strain cultured with sublethal concentrations of antibiotics was visualized with nondenaturing polyacrylamide gel electrophoresis. Total DNA was isolated from equivalent numbers (7.1 × 10^11^) of PK/DNase-treated or untreated EVs from FY26 strain cultured with sublethal concentrations of antibiotics. ‘+’ indicates that samples were treated with PK and DNase I, and ‘−’ indicates that samples were not treated with PK or DNaseI. Naked DNA (pET-32a) was used as a control. (D) The concentrations of purified EVs in multidrug-resistant ExPEC strain ST95-32 cultured with relatively high concentrations of antibiotics (ampicillin, 100 μg/mL; ceftazidime, 50 μg/mL; levofloxacin, 50 μg/mL; chloramphenicol, 30 μg/mL) were determined with NTA. (TIF) [file ppat.1010908.s014.tif]
