# Supplementary material for: Reductions in bacterial viability stimulate the production of Extra-intestinal Pathogenic Escherichia coli (ExPEC) cytoplasm-carrying Extracellular Vesicles (EVs)
Source: PLoS Pathog. 2022 Oct 19;18(10):e1010908. doi: 10.1371/journal.ppat.1010908 (PMC9621596; doi:10.1371/journal.ppat.1010908)
Supplement: S4 Table — (DOCX) [file ppat.1010908.s018.docx]

**S4 Table.** Bacterial strains and plasmids used in this study.

| **Bacterial strains or plasmids** | **Genotype or relevant characteristics** | **Source or Reference** |
| --- | --- | --- |
| **Bacterial strains** |  |  |
| *E. coli* DH5α | Plasmid cloning strain | Vazyme |
| *E. coli* BL21 (DE3) | Plasmid expression strain | Vazyme |
| FY26 | O2:K1; ST95; phylogroup B2 | [1] |
| CBE59 | O2:K1; ST117; phylogroup F | This study |
| CFT073 | O6:K2; ST73; phylogroup B2 | [2] |
| ST95-32 | O1:K1;ST95 | This study |
| FY26Δ*pal* | *pal* deletion in FY26 | This study |
| FY26Δ*epel1* | Scarless deletion of *epel1* in FY26 | This study |
| FY26Δ*epel2.1* | Scarless deletion of *epel2.1* in FY26 | This study |
| FY26Δ*epel2.2* | Scarless deletion of *epel2.2* in FY26 | This study |
| FY26Δ*epel1/2.1* | Parallel scarless deletion of *epel1* and *epel2.1* in FY26 | This study |
| FY26Δ*epel1/2.2* | Parallel scarless deletion of *epel1* and *epel2.2* in FY26 | This study |
| FY26Δ*epel2.1/2.2* | Parallel scarless deletion of *epel2.1* and *epel2.2* in FY26 | This study |
| FY26Δ*epel1/2.1/2.2* | Parallel scarless deletion of *epel1, epel2.1* and *epel2.2* in FY26 | This study |
| FY26C*epel1* | FY26Δ*epel1/2.1/2.2* with plasmid pSTV28-*sul1-epel1* | This study |
| FY26C*epel2.1* | FY26Δ*epel1/2.1/2.2* with plasmid pSTV28-*sul1*-*epel2.1* | This study |
| FY26C*epel2.2* | FY26Δ*epel1/2.1/2.2* with plasmid pSTV28-*sul1*-*epel2.2* | This study |
| FY26Δ*lexA* | *lexA* deletion in FY26 | This study |
| FY26Δ*recA* | *recA* deletion in FY26 | This study |
| FY26Δ*ftsK* | *ftsK* deletion in FY26 | This study |
| FY26C*ftsK* | FY26Δ*ftsK* with plasmid pSTV28-*sul1*-*ftsK* | This study |
| FY26Δ*ftsK/recA* | Parallel deletion of *ftsK* and *recA* in FY26 | This study |
| FY26Δt6A | *ygjD* deletion in FY26 | This study |
| FY26Ct6A | FY26Δ*ygjD* with plasmid pSTV28-*sul1*-*ygjD* | This study |
| FY26Δt6A/*recA* | Parallel deletion of *ygjD* and recA in FY26 | This study |
| FY26-pSTV28-GFP-Sul1 | The recombinant plasmid pSTV28-GFP-Sul1in FY26 | This study |
| CBE59-pSTV28-GFP-Sul1 | The recombinant plasmid pSTV28-GFP-Sul1in CBE78 | This study |
| CFT073-pSTV28-GFP-Sul1 | The recombinant plasmid pSTV28-GFP-Sul1in CFT073 | This study |
| FY26Δ*pal*-pSTV28-GFP-Sul1 | The recombinant plasmid pSTV28-GFP-Sul1in FY26Δ*pal* | This study |
| FY26Δ*epel1*-pSTV28-GFP-Sul1 | The recombinant plasmid pSTV28-GFP-Sul1in FY26Δ*epel1* | This study |
| FY26Δ*epel2.1*-pSTV28-GFP-Sul1 | The recombinant plasmid pSTV28-GFP-Sul1in FY26Δ*epel2.1* | This study |
| FY26Δ*epel2.2*-pSTV28-GFP-Sul1 | The recombinant plasmid pSTV28-GFP-Sul1in FY26Δ*epel2.2* | This study |
| FY26Δ*epel1/2.1*-pSTV28-GFP-Sul1 | The recombinant plasmid pSTV28-GFP-Sul1in FY26Δ*epel1/2.1* | This study |
| FY26Δ*epel1/2.2*-pSTV28-GFP-Sul1 | The recombinant plasmid pSTV28-GFP-Sul1in FY26Δ*epel1/2.2* | This study |
| FY26Δ*epel2.1/2.2*-pSTV28-GFP-Sul1 | The recombinant plasmid pSTV28-GFP-Sul1in FY26Δ*epel2.1/2.2* | This study |
| FY26Δ*epel1/2.1/2.2*-pSTV28-GFP-Sul1 | The recombinant plasmid pSTV28-GFP-Sul1in FY26Δ*epel1/2.1/2.2* | This study |
| FY26Δ*lexA*-pSTV28-GFP-Sul1 | The recombinant plasmid pSTV28-GFP-Sul1in FY26Δ*lexA* | This study |
| FY26Δ*recA*-pSTV28-GFP-Sul1 | The recombinant plasmid pSTV28-GFP-Sul1in FY26Δ*recA* | This study |
| FY26Δ*ftsK*-pSTV28-GFP-Sul1 | The recombinant plasmid pSTV28-GFP-Sul1in FY26Δ*ftsK* | This study |
| FY26Δ*ftsK/recA*-pSTV28-GFP-Sul1 | The recombinant plasmid pSTV28-GFP-Sul1in FY26Δ*ftsK/recA* | This study |
| FY26Δt6A-pSTV28-GFP-Sul1 | The recombinant plasmid pSTV28-GFP-Sul1in FY26Δ*ygjD* | This study |
| FY26Δt6A/*recA*-pSTV28-GFP-Sul1 | The recombinant plasmid pSTV28-GFP-Sul1in FY26Δ*ygjD/recA* | This study |
|  |  |  |
| ***Plasmids*** |  |  |
| pSTV28 | Cm^r^, medium-copy plasmid | Takara |
| pSTV28-*sul1* | pSTV28-*sul1* carrying *epel1* coding region and its putative promoter | This study |
| pSTV28-*sul1*-*epel1* | pSTV28-*sul1* carrying *epel1* coding region and its putative promoter | This study |
| pSTV28-*sul1*-*epel2.1* | pSTV28-*sul1* carrying *epel2.1* coding region and its putative promoter | This study |
| pSTV28-*sul1*-*epel2.2* | pSTV28-*sul1* carrying *epel2.2* coding region and its putative promoter | This study |
| pSTV28-*sul1*-*ftsK* | pSTV28-*sul1* carrying *ftsK* coding region and its putative promoter | This study |
| pSTV28-*sul1*-t6A | pSTV28-*sul1* carrying *ygjD* coding region and its putative promoter | This study |
| pSTV28-GFP-Sul1 | pSTV28 carrying *GFP* and *sul1* coding regions and its putative promoter | This study |
| pSTV28-GFP-Sul1-*epel1* | pSTV28-GFP-Sul1 carrying *epel1* coding region and its putative promoter | This study |
| pSTV28-GFP-Sul1-*epel2.1* | pSTV28-GFP-Sul1 carrying *epel2.1* coding region and its putative promoter | This study |
| pSTV28-GFP-Sul1-*epel2.2* | pSTV28-GFP-Sul1 carrying *epel2.2* coding region and its putative promoter | This study |
| pSTV28-GFP-Sul1-*ftsK* | pSTV28-GFP-Sul1 carrying *ftsK* coding region and its putative promoter | This study |
| pSTV28-GFP-Sul1-t6A | pSTV28-GFP-Sul1 carrying *ygjD* coding region and its putative promoter | This study |
| pET-28a(+) | expression plasmid, Kan^r^ | Novagen |
| pET-28a-*lexA* | pET-28a carrying *lexA* gene | This study |
| pKD4 | Red template plasmid, Kan^r^ | [3] |
| pKD46 | λ-Red recombinase expression, Amp^r^ | [3] |
| pCP20 | encodes FLP recombinase gene, Amp^r^ and Cm^r^ | [3] |
| pKD3 | Red template plasmid, Cm^r^ | [3] |
| pWRG99 | pKD46 with I-SceI endonuclease, temperature-sensitive, Amp^r^, | [4] |

1. Zhuge X, Sun Y, Xue F, Tang F, Ren J, Li D, et al. A Novel PhoP/PhoQ Regulation Pathway Modulates the Survival of Extraintestinal Pathogenic Escherichia coli in Macrophages. Front Immunol. 2018;9:788. doi: 10.3389/fimmu.2018.00788. PubMed PMID: 29719540; PubMed Central PMCID: PMC5913352.

2. Welch RA, Burland V, Plunkett G, Redford P, Roesch P, Rasko D, et al. Extensive mosaic structure revealed by the complete genome sequence of uropathogenic Escherichia coli. P Natl Acad Sci USA. 2002;99(26):17020-4. doi: 10.1073/pnas.252529799. PubMed PMID: WOS:000180101600092.

3. Datsenko KA, Wanner BL. One-step inactivation of chromosomal genes in Escherichia coli K-12 using PCR products. P Natl Acad Sci USA. 2000;97(12):6640-5. doi: DOI 10.1073/pnas.120163297. PubMed PMID: WOS:000087526300074.

4. Blank K, Hensel M, Gerlach RG. Rapid and highly efficient method for scarless mutagenesis within the Salmonella enterica chromosome. Plos One. 2011;6(1):e15763. Epub 2011/01/26. doi: 10.1371/journal.pone.0015763. PubMed PMID: 21264289; PubMed Central PMCID: PMCPMC3021506.
