# Supplementary material for: Reductions in bacterial viability stimulate the production of Extra-intestinal Pathogenic Escherichia coli (ExPEC) cytoplasm-carrying Extracellular Vesicles (EVs)
Source: PLoS Pathog. 2022 Oct 19;18(10):e1010908. doi: 10.1371/journal.ppat.1010908 (PMC9621596; doi:10.1371/journal.ppat.1010908)
Supplement: S5 Table — (DOCX) [file ppat.1010908.s019.docx]

**S5 Table.**PCR primers used in this study

| **Primers** | **Oligonucleotide sequence (5'-3')** | Source or Reference |
| --- | --- | --- |
| **General PCR for cloning** |  |  |
| pSTV28-*epel1*-F | catgattacgaattcgagctcATATTAATGGCACTTATTTATAAGTGCTCT | This study |
| pSTV28-*epel1*-R | acgacggccagtgccaagcttGGGCTTTGTAGGTAATGGCGT | This study |
| pSTV28-*epel2.1*-F | catgattacgaattcgagctcATTAATTCAGGAAGGTCCGCAA | This study |
| pSTV28-*epel2.1*-R | acgacggccagtgccaagcttATTAATCTACATCTGATATTTTTTATCTCTTAAAG | This study |
| pSTV28-*epel2.2*-F | catgattacgaattcgagctcTTCGCTCCCCTTATAACTACGC | This study |
| pSTV28-*epel2.2*-R | acgacggccagtgccaagcttTCACTGCTCTATCCCCCAACA | This study |
| pSTV28-*ftsK*-F | tacgaattcgagctcggtaccTAGACCCAGGCATTGCGC | This study |
| pSTV28-*ftsK*-R | cttgcatgcctgcaggtcgacGCGTCTTATCCGGCCTACG | This study |
| pSTV28-t6A-F | tacgaattcgagctcggtaccTTTACTGCAATTGCTGCTGCTT | This study |
| pSTV28-t6A-R | cttgcatgcctgcaggtcgacTTACCGGCTTACTGTGGATGC | This study |
| pET-28a-*lexA*-F | gtgccgcgcggcagccatatgATGAAAGCGTTAACGGCCAG | This study |
| pET-28a-*lexA*-R | ctcgagtgcggccgcaagcttTTACAGCCAGTCGCCGTTG | This study |
| pSTV28-Sul1-F | ccgccggacatcagcgctagcATGGTGACGGTGTTCGGCA | This study |
| pSTV28-Sul1-R | gccagtatacactccgctagcCTAGGCATGATCTAACCCTCGG | This study |
| pSTV28-GFP-F | gcctgagcaaactggcctcaggCATGATTACGAATTCGAGCTCCC | This study |
| pSTV28-GFP-R | tgtgcttctcaaatgcctgaggCAGGTCGACTCTAGAGGATCCCC | This study |
| **For Scarless deletion** |  |  |
| k1-F | GTGTAGGCTGGAGCTGCTTC | [1] |
| k1-R | TTCTCTAGACTATATTACCCTGTTATCCCTAGCGTAACTAAGTATAGGAACTTCGGCGC | [1] |
| Scadel-*epel1*-F | CTTTATTTCAAGATTAAAGAAGATAAGCGCAAGGCTGCGAGAGGTGAATActagactatattaccctgttatcccta | This study |
| Scadel-*epel1*-R | GCAGACGATGATGCAGATAACCAGAGCGGAGATAATCGCGGTTACTCTGTcgccttacgccccgccctgc | This study |
| Scadel-*epel2.1*-F | AGATTTAACATATCCGGGGATTTGAAGCCGATAAATCCTGATAAATATCCctagactatattaccctgttatcccta | This study |
| Scadel-*epel2.1*-R | ACCTACGACAAATCCTACCAACACGAGCAAAATCACCGTGAATATCTGAT cgccttacgccccgccctgc | This study |
| Scadel-*epel2.2*-F | ACAGATAAACCTGTCCGTGGGCAGAAACCGATAAATCCTGATAAATATCCctagactatattaccctgttatcccta | This study |
| Scadel-*epel2.2*-R | GCAGACGATGATGCAGATAACCAGAGCATAAATAATCGCGGCAACTCTGCcgccttacgccccgccctgc | This study |
| Scadel-*epel1*/*2.1*-F | AGATTTAACATATCCGGGGATTTGAAGCCGATAAATCCTGATAAATATCCctagactatattaccctgttatcccta | This study |
| Scadel-*epel1*/*2.1*-R | ACCTACGACAAATCCTACCAACACGAGCAAAATCACCGTGAATATCTGATcgccttacgccccgccctgc | This study |
| Scadel-*epel1*/*2.2*-F | ACAGATAAACCTGTCCGTGGGCAGAAACCGATAAATCCTGATAAATATCCctagactatattaccctgttatcccta | This study |
| Scadel-*epel1*/*2.2*-R | GCAGACGATGATGCAGATAACCAGAGCATAAATAATCGCGGCAACTCTGCcgccttacgccccgccctgc | This study |
| Scadel-*epel2.1*/*2.2*-F | ACAGATAAACCTGTCCGTGGGCAGAAACCGATAAATCCTGATAAATATCCctagactatattaccctgttatcccta | This study |
| Scadel-*epel2.1*/*2.2*-R | GCAGACGATGATGCAGATAACCAGAGCATAAATAATCGCGGCAACTCTGCcgccttacgccccgccctgc | This study |
| Scadel-*epel1*/*2.1*/*2.2*-F | ACAGATAAACCTGTCCGTGGGCAGAAACCGATAAATCCTGATAAATATCCctagactatattaccctgttatcccta | This study |
| Scadel-*epel1*/*2.1*/*2.2*-R | GCAGACGATGATGCAGATAACCAGAGCATAAATAATCGCGGCAACTCTGCcgccttacgccccgccctgc | This study |
| Fus-*epel1*-F1 | TTTGTCTGCGAGGTGGG | This study |
| Fus-*epel1*-R1 | TCGCGGTTACTCTGTTATTCACCTCTCGCA | This study |
| Fus-*epel1*-F2 | TGCGAGAGGTGAATAACAGAGTAACCGCGA | This study |
| Fus-*epel1*-R2 | AGGCGGTGGTGGCTTCA | This study |
| Fus-*epel2.1*-F1 | CAGGAAGGTCCGCAAGG | This study |
| Fus-*epel2.1*-R1 | GTGAATATCTGATGGATATTTATCAGG | This study |
| Fus-*epel2.1*-F2 | CCTGATAAATATCCATCAGATATTCAC | This study |
| Fus-*epel2.1*-R2 | TGCGTCAAGGCGTATGG | This study |
| Fus-*epel2.2*-F1 | GAGGCAGCAGCCAGAAG | This study |
| Fus-*epel2.2*-R1 | CGGCAACTCTGCGGATATTTATCAGG | This study |
| Fus-*epel2.2*-F2 | CCTGATAAATATCC GCAGAGTTGCCG | This study |
| Fus-*epel2.2*-R2 | CAACCGACGACGACCAG | This study |
| **For Deletion** |  | This study |
| Del-*pal*-F | GTCGCCGTATCTGTGATAATAATTAATTGAATAGTAAAGGAATCATTGAAgtgtaggctggagctgcttc | This study |
| Del-*pal*-R | AACGACAGACTCAATAGTTGATGTCTGAAGTTACTGCTCATGCAATTCTCcatatgaatatcctccttag | This study |
| Del-*lexA*-F | TATACTCACAGCATAACTGTATATACACCCAGGGGGCGGAgtgtaggctggagctgcttc | This study |
| Del-*lexA*-R | AAACCGCGACGCCAGGCGGCATCGCGGTCTCAGAGATATGcatatgaatatcctccttag | This study |
| Del-*recA*-F | TATTGACTATCCGGTATTACCCGGCATGACAGGAGTAAAAgtgtaggctggagctgcttc | This study |
| Del-*recA*-R | AAGGGCCGCAGATGCGACCCTTGTGTATCAAACAAGATGAcatatgaatatcctccttag | This study |
| Del-*ftsK*-F | TGTCCGTTTTAGCATCGGGCAGGAAAAGCCTGTAACCTGGAGAGCCTTTCgtgtaggctggagctgcttc | This study |
| Del-*ftsK*-R | GAATACCGGATGCGACGCTATCGCGTCTTATCCGGCCTACGTTGCATTAAcatatgaatatcctccttag | This study |
| Del-*ftsK/recA*-F | TATTGACTATCCGGTATTACCCGGCATGACAGGAGTAAAAgtgtaggctggagctgcttc | This study |
| Del-*ftsK/recA*-R | AAGGGCCGCAGATGCGACCCTTGTGTATCAAACAAGATGAcatatgaatatcctccttag | This study |
| Del-t6A-F | CTTTTCCACGCTACAGCGTATTGTTAACCCGGAAGTGACTGATAAAAACCgtgtaggctggagctgcttc | This study |
| Del-t6A-R | GCTACCAACCAATTTCTGATGCGATACATCATCCCGCCACCTTTCAAAGGcatatgaatatcctccttag | This study |
| Del-t6A*/recA*-F | TATTGACTATCCGGTATTACCCGGCATGACAGGAGTAAAAgtgtaggctggagctgcttc | This study |
| Del-t6A*/recA*-R | AAGGGCCGCAGATGCGACCCTTGTGTATCAAACAAGATGAcatatgaatatcctccttag | This study |
| **For Mutation verification** |  | This study |
| Check-*epel1*-F | CAACGGGTGTCGCCTAT | This study |
| Check-*epel1*-R | TCAGCTTTCGCATCAGC | This study |
| Check-*epel2.1*-F | CTGCTTGCCTGAGTGCG | This study |
| Check-*epel2.1*-R | TGCGTCAAGGCGTATGG | This study |
| Check-*epel2.2*-F | GAGGCAGCAGCCAGAAG | This study |
| Check-*epel2.2*-R | GCGAAATGTTTACCACGATA | This study |
| Check-*epel1*/*2.1*-F | CTGCTTGCCTGAGTGCG | This study |
| Check-*epel1*/*2.1*-R | TGCGTCAAGGCGTATGG | This study |
| Check-*epel1*/*2.2*-F | GAGGCAGCAGCCAGAAG | This study |
| Check-*epel1*/*2.2*-R | GCGAAATGTTTACCACGATA | This study |
| Check-*epel2.1*/*2.2*-F | GAGGCAGCAGCCAGAAG | This study |
| Check-*epel2.1*/*2.2*-R | GCGAAATGTTTACCACGATA | This study |
| Check-*epel1*/*2.1*/*2.2*-F | GAGGCAGCAGCCAGAAG | This study |
| Check-*epel1*/*2.1*/*2.2*-R | GCGAAATGTTTACCACGATA | This study |
| Check-*pal*-F | CGTCAGGTGGCTTCATTC | This study |
| Check-*pal*-R | TGTTGCTGGAGTTGGGTT | This study |
| Check-*lexA*-F | GAAGGCGTAGCGGTATTG | This study |
| Check-*lexA*-R | ATGGGTAAGGCGAGATGC | This study |
| Check-*recA*-F | GCTTCCCTCTGGCTAATC | This study |
| Check-*recA*-R | GCAACTACCTGATGTCCCT | This study |
| Check-*ftsK*-F | GACAGGAGTAGGGAAGGAATA | This study |
| Check-*ftsK*-R | CCAGTTGAATAAGTTTGGACG | This study |
| Check-*ftsK/recA*-F | GCTTCCCTCTGGCTAATC | This study |
| Check-*ftsK/recA*-R | GCAACTACCTGATGTCCCT | This study |
| Check-t6A-F | GCACGGCCTTACAACTACAA | This study |
| Check-t6A-R | CCACCGCTCTATCAATCACA | This study |
| Check-t6A*/recA*-F | GCTTCCCTCTGGCTAATC | This study |
| Check-t6A*/recA*-R | GCAACTACCTGATGTCCCT | This study |
| **For EMSA** |  |  |
| P*_epel1_*-F | TCAGCAGGTAGTGCCGGGTACTGGT | This study |
| P*_epel1_*-R | AGCAACGGCTTTTCGTAATGATGGA | This study |
| MutP*_epel1_*-F1 | TCAGCAGGTAGTGCCGGGTACTGGT | This study |
| MutP*_epel1_*-R1 | TGAAATAAAGGTTTAACAAATAGGTCTTCAGGCCAAATAC | This study |
| MutP*_epel1_*-F2 | GTATTTGGCCTGAAGACCTATTTGTTAAACCTTTATTTCA | This study |
| MutP*_epel1_*-R2 | AGCAACGGCTTTTCGTAATGATGGA | This study |
| P*_epel2.1_*-F | TGAGGAGGGTAAATCTC | This study |
| P*_epel2.1_*-R | GCTGATTTAATCGCACT | This study |
| MutP*_epel2.1_*-F1 | TGAGGAGGGTAAATCTC | This study |
| MutP*_epel2.1_*-R1 | GGAATTAGTTAGGGCAATTCTTATTAAAACTGCCGCCG | This study |
| MutP*_epel2.1_*-F2 | CGGCGGCAGTTTTAATAAGAATTGCCCTAACTAATTCC | This study |
| MutP*_epel2.1_*-R2 | GCTGATTTAATCGCACT | This study |
| P*_epel2.2_*-F | TGACCTGGACATCTCGC | This study |
| P*_epel2.2_*-R | TTCCTGGTAGCTTTCCT | This study |
| MutP*_epel2.2_*-F1 | TGACCTGGACATCTCGC | This study |
| MutP*_epel2.2_*-R1 | CTTCACATAACCGCGCGAATTCATCCTTGAAGACCTGTCTG | This study |
| MutP*_epel2.2_*-F2 | CAGACAGGTCTTCAAGGATGAATTCGCGCGGTTATGTGAAG | This study |
| MutP*_epel2.2_*-R2 | TTCCTGGTAGCTTTCCT | This study |
| Con*-pal*-F | CTCTTCTCAGGGGATGG | This study |
| Con*-pal*-R | AACAGGCAGAGCAATCA | This study |
| **For RT-PCR** |  |  |
| orf00224-qPCR-F | CTATCAGCAGCTTTACCTGTTCT | This study |
| orf00224-qPCR-R | GATCAACTGAATGGCGAGTCT | This study |
| orf00225-qPCR-F | GTGTTGTTCTGGTGCAGTTTC | This study |
| orf00225-qPCR-R | CGCTGAATGGCAATCTCAATC | Novagen |
| orf00543-qPCR-F | GGCATTGCAGCAGATTAAAGAG | This study |
| orf00543-qPCR-R | GGCAGTGAAGCCCAGATATT | This study |
| orf00544-qPCR-F | GTACCTTCGCGGCAGATATAA | This study |
| orf00544-qPCR-R | GTCGAGAAGGTCACGAATGAA | This study |
| orf00843-qPCR-F | GGTGGGATGCATACCGTAAA | This study |
| orf00843-qPCR-R | GTAAAGCACCACGCTCTTTAATC | This study |
| orf00844-qPCR-F | CGGTGTTATTGGTGGCCTAA | This study |
| orf00844-qPCR-R | AACCCATCGCACCACTAATC | This study |
| orf00865(*epel2.1*)-qPCR-F | ATTGGCCCTGGTAAGTGTTT | This study |
| orf00865(*epel2.1*)-qPCR-R | CACGTCCACCGTCCTTTATC | This study |
| orf00867-qPCR-F | AACAACAGGTATCGCCTATGG | This study |
| orf00867-qPCR-R | CCACTGTGACGGAGTTACTTTAT | This study |
| orf03994-qPCR-F | ATGGTACCTCAGCAGGTAGT | This study |
| orf03994-qPCR-R | GTACGTCAGCAGGCCAAATA | This study |
| orf03995(*epel1*)-qPCR-F | TTGTTGGTGTATGGACTGTATGT | This study |
| orf03995(*epel1*)-qPCR-R | ACCGTGGCAAGGTCTTTATT | This study |
| orf04034-qPCR-F | CAGTGCTGGCTACTGGTTT | This study |
| orf04034-qPCR-R | GATATGTCAGGAAGCCCAGAAC | This study |
| orf04036(*epel2.2*)-qPCR-F | GGTAACCACACTACGGCATAC | This study |
| orf04036(*epel2.2*)-qPCR-R | CGACAGTTTCATACCGGGAAA | This study |
| orf01632-qPCR-F | GCTGGGTAGCCTGGTATTTG | This study |
| orf01632-qPCR-R | CAGCCTTGCGCTTATCTTCT | This study |
| orf01633-qPCR-F | GTTGCTGCTGCTATTGGTGG | This study |
| orf01633-qPCR-R | CACACCTTCCAGACCATCGT | This study |
| orf02067-qPCR-F | GGCTACTGGTTCCTGCAGTT | This study |
| orf02067-qPCR-R | TACGTCAGCAGCCCAAACAG | This study |
| orf02069-qPCR-F | TCCGTACAACATTGGTCCCG | This study |
| orf02069-qPCR-R | TGCCACCGTCCTTAATCCAC | This study |
| orf02449-qPCR-F | TGGTGGATTAAGGATGGCGG | This study |
| orf02449-qPCR-R | GGTTAATGCGCTCTCCTGGT | This study |
| orf02451-qPCR-F | TCACAGTGGGCAGCAATAG | This study |
| orf02451-qPCR-R | GCCTTACGCCTGTCTTCTTTA | This study |
| orf02898-qPCR-F | CCGCGATGGTTCCGGTATAT | This study |
| orf02898-qPCR-R | CGACAGCTTCATTCCCGGTA | This study |
| orf02900-qPCR-F | ACAGTGGCTTGATCAGGTCAG | This study |
| orf02900-qPCR-R | AAGTCAGGAAGCCCAGAACC | This study |
| orf03205-qPCR-F | GCGCCTGATATTCTCGACCA | This study |
| orf03205-qPCR-R | CCCGACAGATGGTCCAGATG | This study |
| orf03207-qPCR-F | CAATAGGTGTGCTGGGTAGTC | This study |
| orf03207-qPCR-R | GCCTTACGCTTGTCTTCTTTAATC | This study |
| AAN79429.1-qPCR-F | CGAATCAGCGCCTTTATCACG | This study |
| AAN79429.1-qPCR-R | AGCGTTGAGGCATCCTTGAT | This study |
| AAN79430.1-qPCR-F | GTTAAACGCTGCCTTGTCGG | This study |
| AAN79430.1-qPCR-R | ACAGCCTTCGTAATCGGCAA | This study |
| AAN79902.1-qPCR-F | AGTTGCTGGACAGGGTTTC | This study |
| AAN79902.1-qPCR-R | TACGACGGTCCTCTCTGATT | This study |
| AAN79905.1-qPCR-F | GCGCCTGATATTCTCGACCA | This study |
| AAN79905.1-qPCR-R | CGGGCTTACCATCCACCATT | This study |
| AAN80030.1-qPCR-F | GTCGCCTATGGTACCTCAGC | This study |
| AAN80030.1-qPCR-R | TCAGCAGGCCAAATACCAGG | This study |
| AAN80031.1-qPCR-F | GCGGCGCTCTTTACTCATTC | This study |
| AAN80031.1-qPCR-R | CATGCGCCTTTGATATCGCC | This study |
| AAN81632.1-qPCR-F | ATTGGCCCTGGTAAGTGTTT | This study |
| AAN81632.1-qPCR-R | CACGTCCACCGTCCTTTATC | This study |
| AAN81634.1-qPCR-F | TGTCAGATACGTCAGCAGGC | This study |
| AAN81634.1-qPCR-R | TGGGCATTGCAGTTACTCGA | This study |
| *dnaE*-qPCR-F | ATGTCGGAGGCGTAAGGCT | [2] |
| *dnaE* -qPCR-R | TCCAGGGCGTCAGTAAACAA | [2] |

**P_tac_-GFP-terninator sequence for DNA synthesis**

CCGGAATTCTCGGCTCGTATAATGTGTGGAATTGTGAGCGGATAACAATTTCACACAGGAAACAGCCAGTCCGTTTAGGTGTTTTCACGAGCACTTCACCAACAAGGACCATAGCATATGGTGAGCAAGGGCGAGGAGCTGTTCACCGGGGTGGTGCCCATCCTGGTCGAGCTGGACGGCGACGTAAACGGCCACAAGTTCAGCGTGTCCGGCGAGGGCGAGGGCGATGCCACCTACGGCAAGCTGACCCTGAAGTTCATCTGCACCACCGGCAAGCTGCCCGTGCCCTGGCCCACCCTCGTGACCACCCTGACCTACGGCGTGCAGTGCTTCAGCCGCTACCCCGACCACATGAAGCAGCACGACTTCTTCAAGTCCGCCATGCCCGAAGGCTACGTCCAGGAGCGCACCATCTTCTTCAAGGACGACGGCAACTACAAGACCCGCGCCGAGGTGAAGTTCGAGGGCGACACCCTGGTGAACCGCATCGAGCTGAAGGGCATCGACTTCAAGGAGGACGGCAACATCCTGGGGCACAAGCTGGAGTACAACTACAACAGCCACAACGTCTATATCATGGCCGACAAGCAGAAGAACGGCATCAAGGTGAACTTCAAGATCCGCCACAACATCGAGGACGGCAGCGTGCAGCTCGCCGACCACTACCAGCAGAACACCCCCATCGGCGACGGCCCCGTGCTGCTGCCCGACAACCACTACCTGAGCACCCAGTCCGCCCTGAGCAAAGACCCCAACGAGAAGCGCGATCACATGGTCCTGCTGGAGTTCGTGACCGCCGCCGGGATCACTCTCGGCATGGACGAGCTGTACAAGTAACGGCAGTAGCGCGGTGGTCCCACCTGACCCCATGCCGAACTCAGAAGCTTGGG

**References**

1. Chen MM, Zhang L, Xin SP, Yao HC, Lu CP, Zhang W. Inducible Prophage Mutant of Escherichia coli Can Lyse New Host and the Key Sites of Receptor Recognition Identification. Front Microbiol. 2017;8. doi: ARTN 147

10.3389/fmicb.2017.00147. PubMed PMID: WOS:000393157200001.

2. Wang SH, Bao YL, Meng QM, Xia YJ, Zhao YC, Wang Y, et al. IbeR Facilitates Stress-Resistance, Invasion and Pathogenicity of Avian Pathogenic Escherichia coli. Plos One. 2015;10(3). doi: ARTN e0119698

10.1371/journal.pone.0119698. PubMed PMID: WOS:000351277500094.
