# Supplementary material for: Reductions in bacterial viability stimulate the production of Extra-intestinal Pathogenic Escherichia coli (ExPEC) cytoplasm-carrying Extracellular Vesicles (EVs)
Source: PLoS Pathog. 2022 Oct 19;18(10):e1010908. doi: 10.1371/journal.ppat.1010908 (PMC9621596; doi:10.1371/journal.ppat.1010908)
Supplement: S6 Table — (DOCX) [file ppat.1010908.s020.docx]

**S6 Table.** The peptides sequence used in this study.

| proteins | peptides sequence |
| --- | --- |
| OmpA | GFATVAQAAPKDNT |
|  | FTFNKATLKPEGQ |
| Lpp | TKLVLGAVILGSTL |
|  | AAKDDAARANQRL |
| Pal | LNKVLKGLMIALPV |
|  | QGKGVSADQISIV |
| AdhE | KHLINKKTVAKRAE |
|  | TGANPRYPLISELK |
| AtpD | KIVQVIGAVVDVEF |
|  | QLDPLVVGQEHYDT |
| CRP | TDPTLEWFLSHCH |
|  | KQPDAMTHPDGMQ |
| AckA | RIKWKMDGNKQE |
|  | TSDCRYVEDNYATK |
| PGK | GRPTEGEYNEEFS |
|  | TLAAIDLFGIADKI |
| Pyk | AVTYEGFTTDLSVG |
|  | TNEKTAHQLVLSKG |
| GAPDH | AAQKRSDIEIVAIN |
|  | TQKTVDGPSHKDW |
| RP-L2 | GRRHVVKVVNPELH |
|  | QTKGKKTRSNKRTDK |
| RP-L15 | KAGKRLGRGIGSGL |
|  | LAGEVTTPVTVRGL |
| RP-S3 | TKEFADNLDSDFKV |
|  | AAQPKKQQRKGRK |
| RP-S5 | KLIAVNRVSKTVK |
|  | VAAKRGKSVEEIL |
| Epel1 | RKAVAAAIGGGAIA |
|  | KQWKGLMTRREIE |
| Epel2 | TAYRDGSGIWTICR |
|  | STFYKRLNAGDRK |
